# The Dutch ‘Focus on Strength’ intervention study protocol: programme design and production, implementation and evaluation plan

**DOI:** 10.1186/s12889-016-3150-6

**Published:** 2016-06-10

**Authors:** G. A. Ten Hoor, G. Kok, G. M. Rutten, R. A. C. Ruiter, S. P. J. Kremers, A. M. J. W. Schols, G. Plasqui

**Affiliations:** Department of Human Biology, Nutrition and Translational Research in Metabolism, Maastricht University Medical Centre+, P.O. Box 616, 6200 MD Maastricht, The Netherlands; Department of Work and Social Psychology, Maastricht University, P.O. Box 616, 6200 MD Maastricht, The Netherlands; Department of Health Promotion, Nutrition and Translational Research in Metabolism, Maastricht University Medical Centre+, P.O. Box 616, 6200 MD Maastricht, The Netherlands; Department of Respiratory Medicine, Nutrition and Translational Research in Metabolism, Maastricht University Medical Centre+, P.O. Box 616, 6200 MD Maastricht, The Netherlands

**Keywords:** Obesity, Strength exercise, Social comparison, Motivational interviewing, School-based, Randomized controlled trial, Intervention mapping

## Abstract

**Background:**

Overweight youngsters are better in absolute strength exercises than their normal-weight counterparts; a physiological phenomenon with promising psychological impact. In this paper we describe the study protocol of the Dutch, school-based program ‘Focus on Strength’ that aims to improve body composition of 11–13 year old students, and with that to ultimately improve their quality of life.

**Methods:**

The development of this intervention is based on the Intervention Mapping (IM) protocol, which starts from a needs assessment, uses theory and empirical research to develop a detailed intervention plan, and anticipates program implementation and evaluation. This novel intervention targets first year students in preparatory secondary vocational education (11–13 years of age). Teachers are the program implementers. One part of the intervention involves a 30 % increase of strength exercises in the physical education lessons. The other part is based on Motivational Interviewing, promoting autonomous motivation of students to become more physically active outside school. Performance and change objectives are described for both teachers and students. The effectiveness of the intervention will be tested in a Randomized Controlled Trial in 9 Dutch high schools.

**Discussion:**

Intervention Mapping is a useful framework for program planning a school-based program to improve body composition and motivation to exercise in 11–13 year old adolescents by a “Focus on Strength”.

**Trial registration:**

NTR5676, registered 8 February 2016 (retrospectively registered).

**Electronic supplementary material:**

The online version of this article (doi:10.1186/s12889-016-3150-6) contains supplementary material, which is available to authorized users.

## Background

Obesity is a growing health problem globally [38, 47]. It is an established risk factor for chronic metabolic and cardiovascular diseases [22, 55]. In overweight and obese children and adolescents, not only metabolic health, but also psychological wellbeing is at risk [16, 22, 52]. Besides overeating and genetic susceptibility, an insufficient level of physical activity is one of the main contributors to childhood overweight and obesity [25], and the target of many obesity reduction programmes [26]. However, most of these interventions are not successful (see e.g. meta-analyses by Guerra, Nobre, Silveira, & Taddei, [17]; Guerra, Nobre, da Silveira, & Taddei, [18]; Harris, Kuramotoda, Schulzer, & Retallack, [19]; [33]).

In this paper we describe the study protocol of the new, Dutch, school-based ‘Focus on Strength’ intervention. Recent evidence indicates plausible effects of the role of strength exercises in combating the negative health effects of childhood obesity (see e.g. [29]; summarized in [48, 49]). Overweight youngsters do not only have a higher fat mass, but also a higher fat-free (muscle) mass compared with their normal-weight counterparts ([56]; Ten Hoor, unpublished data). With that, they are also stronger and better in exercises wherein the focus is on absolute strength, making them – under the right circumstances – more motivated to engage in strength exercise and ultimately maintain a physically active lifestyle. In the past, it has been suggested that strength exercises are harmful for youngsters, particularly during growth (i.e. growth plate injuries or stunted growth). However, more recent data indicate that this is a persistent misperception devoid of any evidence [3, 5, 6, 12, 13, 29]. As long as strength exercises are performed under qualified supervision, they can even prevent injuries and cause a rapid rehabilitation from injuries [29, 44]. Lately, the short-term and long-term benefits of youth resistance exercises have become more and more evident (for an elaborate overview, see [29]). Although resistance exercises do not reduce weight or BMI per se [21], they can induce a shift in body composition by increasing one’s fat-free mass [9, 43], strength, motor skills, and energy balance on the long term [29, 39, 54].

In the Focus on Strength programme, we do not focus on what youngsters have to do, but we try to (intrinsically) motivate youngsters to engage in physical activity health behaviour that they like to do, thus promoting behaviour change maintenance [48, 49]. We aim to minimize obesity stigma by focusing on the general 11–13 year old high school population, and not only on overweight or obese youngsters. Also, we do not aim to focus on weight loss, or weight adjusted for height (BMI) improvements, but on improvements in body composition. Compared to body mass or BMI, body composition (ratio fat free mass: fat mass) is a better predictor of health, also for young people [11].

**Fig. 1:**
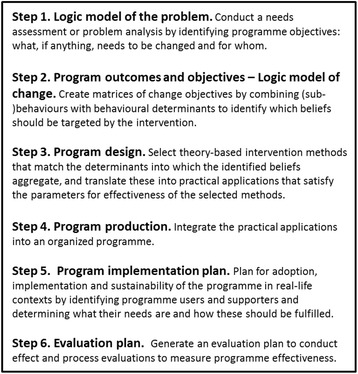
Intervention Mapping steps

The development of the Dutch, school-based ‘Focus on Strength’ intervention is based on the Intervention Mapping (IM) protocol [4, 24]. IM describes the iterative path from problem identification to problem solving or mitigation. The six steps of IM comprise several tasks each of which integrates theory and evidence. The completion of the tasks within a step creates a product that guides the subsequent step. The completion of all of the steps serves as a blueprint for designing, implementing and evaluating an intervention based on theoretical, empirical and practical information. The six steps of the IM process are displayed in Fig. 1.

The key words in IM are planning, research, and theory. IM provides a vocabulary for programme planning, procedures for planning activities, and technical assistance with identifying theory-based determinants, and matching them with appropriate methods for change at different ecological levels including target group, stakeholders, environmental agents, and programme implementers.

## Methods and results

This intervention focuses on first year students in preparatory secondary vocational education (11–13 years of age) as target population, and their teachers as implementers. Therefore, in this school-based approach, there is both a students’ programme and a teachers’ programme. For step 1 to 4 of the IM protocol, the two pathways are described in parallel.

A school-based approach may induce stigmatization in particular when an intervention only would include a focus on overweight and obese youngsters. Therefore, in accordance with the merits of social comparison theory [28, 45, 53], we chose to develop a programme in which overweight and normal weight youngsters exercise together. Because our programme includes the general 11–13 year old high school population, and not only on overweight and obese youngsters, we strongly reduce the risk of stigmatization [41]. Although the entire programme development focuses on all youngsters, it is expected to be more beneficial for overweight and obese youngsters in terms of programme outcomes. Given that overweight youngsters are better in strength exercises than normal weight youngsters, overweight youngsters may find out that they perform better in the domain of strength exercises (contrary to the domain of aerobic exercises) which, in time, is hypothesized to improve their feelings of competence and relatedness and their self-worth [45].

Some authors have suggested that stimulating social comparison may have detrimental effects on autonomous motivation [1] while others suggest that social comparison is part of typical classroom settings and that perceptions of competence and relatedness are predictive of autonomous motivation [49]. Positive social experiences with strength exercises may, in time, increase intrinsic motivation for exercise in overweight youngsters. Moreover, having youngsters compete as teams in multi-component exercises, combining aerobic and strength tasks, might encourage interpersonal appreciation of various skills, e.g. speed vs strength.

### Step 1 and Step 2: Needs assessment and programme objectives

In the first step of IM, the health problem, the related behaviour and the associated determinants for the at-risk population need to be clarified. In this needs assessment, a description of a specific health problem, its impact on quality of life and behavioural and environmental causes and determinants are formulated (as indicated in the Background). In the second IM step, behavioural, performance and change objectives are formulated. Here, the foundation for the intervention will be provided by specifying who and what will change when the intervention will be executed.

#### Programme objectives

**Fig. 2:**
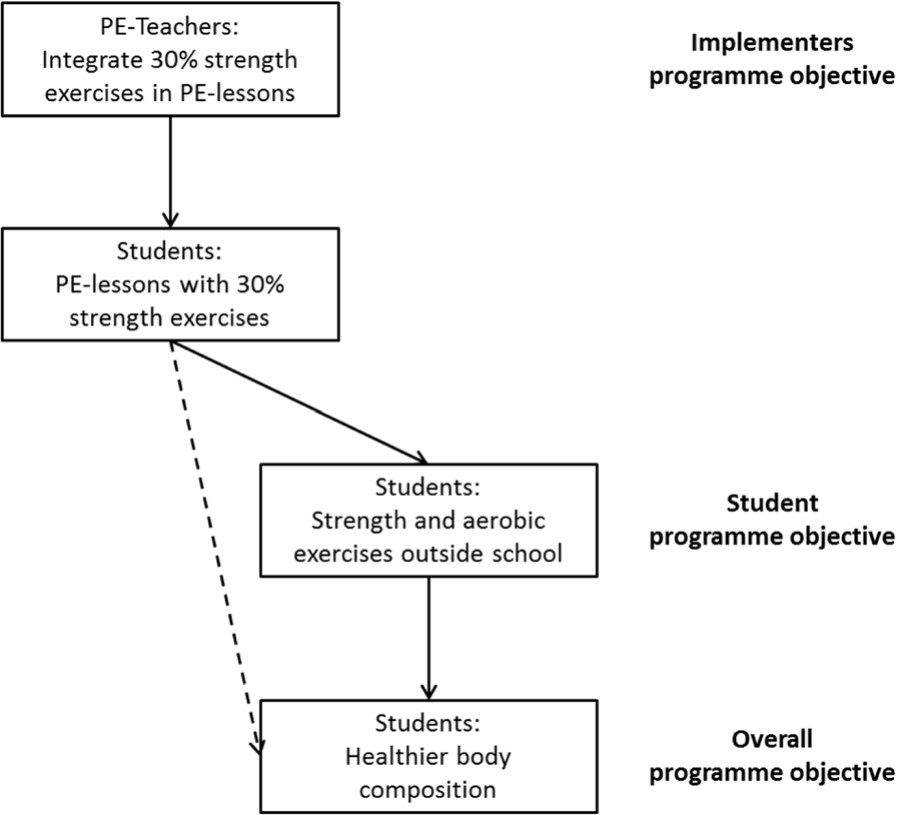
Programme objectives

The overall objective of this intervention is to improve body composition of 11–13 year old students, and with that to ultimately improve their quality of life. Participation of all stakeholders is guaranteed through regular meetings with the school management, and teachers, and a survey among students. The most important implementers are the teachers. To improve body composition, the health promoting behavioural programme objective for the physical education (PE) teachers is to promote strength exercises in their students. This requires a sharper focus on strength exercises in physical education lessons, resulting in the students spending at least 30 % of the PE lessons on strength exercises (an average of approximately 15 min per lesson). The choice for 30 % was the outcome of meetings with PE teachers about the feasibility of integrating strength exercises in the standard curriculum. The behavioural programme objective for the students is that they, in addition to the PE lessons, become more physically active outside school (i.e. at least 1 h/day of physical activity per day, and preferably more, according to internationally accepted recommendations) (See Fig. 2).

#### Performance objectives for teachers and students

Performance objectives are the description of the specific preparatory and sub-behaviours that the students and teachers have to perform to achieve the desired change. For adding more strength exercises in PE classes, the performance objectives that were chosen in collaboration with the PE teachers are: plan, prepare and adapt strength exercises for their lessons; locate appropriate (safe) equipment, and if not or not sufficiently available, get (extra) support from the school’s management; adapt and continue strength exercises through the school year (see Table 1).

**Table 1:** Matrix of change objectives for the PE teacher

Program objective: 30 % more strength exercises in PE class.	Determinants			
Performance objectives	Knowledge	Skills & Self-Efficacy	Attitude	Perceived norms
PO1. Plan strength exercises in PE classes	K1.1. State the advantages (biologically and psychologically) of strength exercises in obese youngsters. K1.2. State that body composition improvements are more important than weight loss. K1.3. State that strength exercises improve body composition. K1.4. State that strength exercises are good for all students.	SSE1.1. Demonstrate the ability to add strength exercises to the PE classes. SSE1.2. Express confidence to add 30 % more strength exercises to all gym classes.	A1.1 Express that adding strength exercises has many more advantages than disadvantages. A1.2. Belief that the school is co-responsible for student’s health, and that strength exercises contribute.	PN1.1. Explain that other PE teachers also plan strength exercises in PE classes.
PO2. Prepare strength exercises, (and use the workbook for inspiration).	K.2.1. List a sufficient number of strength exercises that can be used throughout the year, and which are appropriate for 11–13 year old students K2.2. State what is needed to maintain safety.	SSE2.1. Prepare lessons for PE classes SSE2.2. Express confidence that they can create strength exercises that can be used throughout the year, and which are appropriate for 11–13 year old students	A2.1. Belief that it’s important to plan strength exercises ahead over the year.	
PO3. Adapt strength exercises based on experience.	K3.1. Indicate the differences among students (e.g. gender and physical development).	SSE3.1. Express confidence that they can give strength exercises appropriate for 11–13 year old students.	A3.1. State that well-adjusted, tailored strength exercises are advantageous for the students.	PN2.1. Recognize strength exercises as important aspects of PE class.
PO4. Locate appropriate (safe) equipment	K4.1. Explain what available equipment at the school is appropriate and safe for strength exercises.		A4.1. State importance of having sufficient, appropriate and safe equipment.	
PO5. Acquire additional materials through school management.	K5.1. Explain what new materials are needed		A5.1. Belief that the school is co-responsible for student’s health, and that strength exercises contribute. A5.2. Belief that strength exercises can be positively discussed with the principle.	PN5.1 Talk about other school managements that acquire additional materials for strength exercises.
PO6. Adapt and Continue the strength exercises through the school year.	K6.1. List possible alternatives for strength exercises that might be more appropriate.	SSE6.1. Feel confident to deal with possible barriers.	P6.1. Belief that long term benefits can be achieved by continuation of the strength exercises throughout the school year.	

To improve their level of physical activity, students have to go through a process of self-regulation related to many aspects of physical activity [31]. They first have to monitor their current physical activity situation, relate that to physical activity norms, and decide to increase own physical activities. Then, the students make action plans, experience different kinds of physical activities, and discover what kind of physical activity they like. Then they identify and eliminate possible barriers. Finally, students have to continue their physical activity behaviour over time (see Table 2).

**Table 2:** Matrix of change objectives for the student

Program objective: Be physically active for at least 1 h/day after school	Determinants			
Performance objectives	Knowledge	Skills & Self-Efficacy	Attitude	Perceived Norms
PO1. Monitor own physical activity behavior.	K1.1 Rate own physical activity (1–10),			PN1.1 Recognize that peers are physically active after school
PO2. Evaluate own physical activity behavior.	K2.1 Explain why physical activity was not rated 2 points lower.(followup on K1.1)			PN2.1. Talk to peers about their physical activity behaviour.
PO3. Decide to increase own physical activity	K3.1 State 2 reasons why one should be physically active K3.2. Formulate physical activity goals	SSE1. Write down weekly physical activities	A3.1.Lists advantages of physical activity and disadvantages of physical inactivity.	PN1. State that 1 h/day physical activity is the generally accepted norm.
PO4. Make action plans to be physically active	K4.1.1. Lists places where one can be physically active	Expresses own physical activity qualities	Express positive attitudes toward action plans to be physically active	
PO4.1 Choose sports or physical activity	K4.1. List own positive qualities for physical activity.	SSE4.1.1. Recognize that different athletes have different qualities.	A4.1.1. Value own qualities as good/positive. A4.1.2. Express enjoyment in exercise of choice	PN4.1.1. Recognize that peers value their skills.
PO4.2 Get support from parents and peers.	K4.2.1 Name friends who want to join in physical activity K4.2.2. Explain how parents and peers can help to be physically active.			
PO4.3 Identify and eliminate barriers to start.	K4.3.1. Identify and eliminate barriers to start.			PN4.1. Recognize how peers deal with barriers to start
PO5. Identify and eliminate barriers for continuation of physical activity.	PO5.1 Describe possible barriers and solutions for continuation of physical activity			PN5.1. Recognize how peers deal with barriers for commencement and continuation
PO6. Recycle to monitoring.				

#### Change objectives for teachers and students

Change objectives describe what needs to change, related to the determinants, for the person to execute the performance objectives. Change objectives combine determinants and performance objectives and are the basis for choosing theory- and evidence-based change methods and other program content. To change the behaviour with the performance objectives in mind, the most important and changeable determinants of the behaviour should be taken into account. In this programme, these determinants are based on theory (Theory of Planned Behaviour/Reasoned Action Approach; [15]; Self Determination Theory, [10]; Social Comparison Theory, [53]) and on meetings with teachers as well as (unpublished) survey data from students. In Tables 1 and 2 the change objectives for students and PE teachers are presented.

For PE teachers, the current situation in many high schools is that they have little support for the execution of strength exercises.

From interviews with PE teachers, the most important change objectives derived were that they are aware of the positive influences of strength exercises, that they have a positive over-all attitude about strength exercises, and that they have a high perceived support from their environment, i.e. the school management, and other PE teachers. Furthermore, it is important that they have high self-efficacy and perceived skills to create the possibilities to execute strength exercises in their lessons.

Change objectives for the students are that they know the advantages and disadvantages of physical activity and inactivity, and understand that different physical activities require different qualities (e.g. a 100 kg judoka is not a good 100 m distance runner and vice versa). They develop a more positive attitude (e.g. fun), perceived norm and self-efficacy towards their own physical activity behaviour outside school. Finally, students report a higher intention, are more aware of the possibilities for exercising outside school, and more autonomously motivated to engage in physical exercise.

### Step 3 and Step 4: Programme plan and design

To achieve the overall programme objective of a healthier body composition in students, the PE teachers are the intermediates that implement the strength program. However, the implementation of strength exercises in PE lessons is not sufficient to achieve the programme objective for the students to enhance their out of school PA level. To help the students through the process of self-regulation related to physical activity, a motivational program was developed for this study. This motivational program was based on Motivational Interviewing [34, 36]. Motivational Interviewing is:

*“A collaborative, goal-oriented style of communication with particular attention to the language of change. It is designed to strengthen personal motivation for and commitment to a specific goal by eliciting and exploring the person’s own reasons for change within an atmosphere of acceptance and compassion”* ([34], pp. 29).

**Fig. 3:**
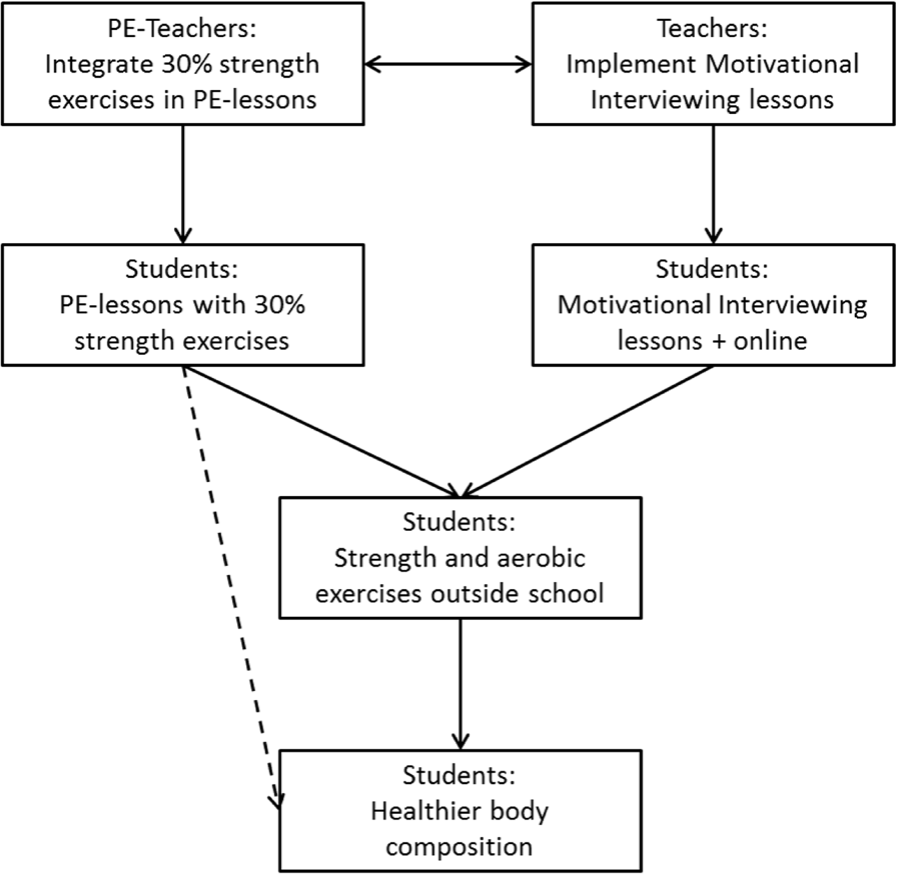
Overall design of the focus on strength programme

In Fig. 3 the overall design of the intervention is presented. The PE teachers integrate strength exercises in their PE lessons; overweight and obese children experience higher levels of competence and relatedness; students learn that strength exercises can be fun; a motivational intervention including autonomy support intends to enhance feelings of autonomy. Consequently, this combination of adjusted PE lessons and a motivational intervention would result in the enhanced feelings of autonomy, competence an relatedness that are required to increase the autonomous motivation for PA that helps students to continue engaging in out of school sports activities.

**Fig. 4:**
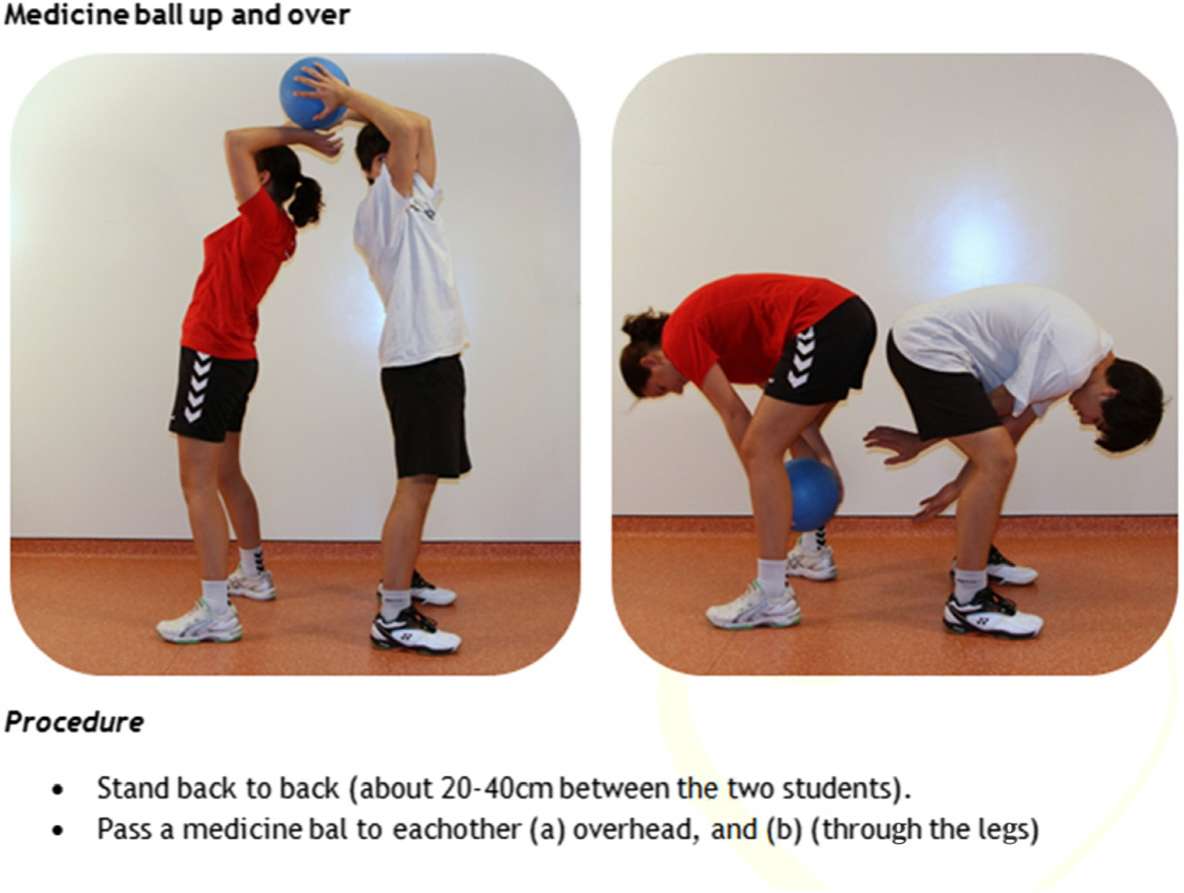
Example of the teacher’s book with strength exercises

The methods used to prepare the PE teachers for the intervention are facilitation and participatory problem solving (see [4], pp. 378 and 391). The teachers are instructed about the program, participate in workshops to improve their motivational speaking skills, and are provided with materials to make them able to include strength exercises in their lessons. Furthermore, teachers receive a book with strength exercises and games as inspirational material. This inspirational material is based on literature, ideas from experts in the field, and from the PE teachers themselves (see Fig. 4 for an example, and Additional file 1 for the complete Dutch book).

To motivate students to be more physically active after school, and to improve the determinants of their physical activity behaviour, the basic principles of Motivational Interviewing are applied. All students receive a workbook and once a month lessons to increase their motivation to be physically active outside school. The motivational intervention challenges students to make their own decisions and choices, herewith appealing to their feeling of autonomy. Together with the feelings of competence and relatedness the students experience during the PE lessons, the complete programme, therefore, aims at improving all three of the basic psychological needs required for autonomous motivation [42]. The motivational lessons are facilitated by a trained mentor or PE teacher. In the first 5 months, a monthly extra online motivational lesson is given, in which students are provided with the opportunity to establish a shielded personal environment in which they do not feel judged by their fellow classmates. See Table 3 and Additional file 2 for the content of each motivational lesson.

**Table 3:** Content per motivational lesson

Lesson	Class/Online	Topic	Motivational interviewing
1	C	- Own physical activity behaviour - (anonymous) comparison to group norms	In this lesson, students become aware of their own physical activity behaviour. Based on the anonymous physical activity group mean, students can compare and evaluate their own physical activity behaviour
2	O	- Perceived level of own physical activity - Prepare lesson 3.	Students are asked to give a grade to their own physical activity behaviour (1–10). After this, they are asked why they did not score 2 points lower. The idea here is that students come up with things they *are* doing.
3	C	- Advantages and disadvantages of physical activity and inactivity	The students discuss all advantages and disadvantages of physical activity and inactivity to create ambivalence.
4	O	- Physical activity and sedentary norms - Prepare lesson 4.	Students are made aware of the current physical activity norms (at 60 min of physical activity per day) and sedentary guidelines (less than 2 h of sedentary behaviour per day).
5	C	- Awareness of different qualities of different athletes.	Different athletes are compared by means of Youtube videos. During this lesson, students are made aware that different physical activities require different qualities (e.g. a 100 kg judoka is not a good 100 m distance runner and vice versa).
6	O	- What physical activity suits me?	See also Additional file 2. this is a table/exercise adapted from the book ‘Bewegen, Sport en Maatschappij’ (physical activity, sports and society) [8] by Boon, Pecht, Rijper & Stegeman.
7	C	- Action planning	First the students are asked how confident they are to start or commence a physical activity. In the action plan the student describes the what, when, where, and how (what can they do themselves, who do they need, where can they find help) of their physical activity plan.
8	O	- Synthesis of lesson 1-7	Students write a short essay about what they want to do, what they want to achieve, and why.
9	C	- Commitment to the action plan	Students discuss how they will try to achieve their goals, and help each other when necessary.
10	O	- Improvement of action plan	
11	C	*Catch up month*	
12	C	- Own physical activity behaviour - (anonymous) comparison to group norms - Action	Repetition of lesson 1. In this lesson, the students also have to come up with an idea of what physical activity behaviour they want to start in the coming 2 months.
13	C	*Catch up monthn* *Action month*	Students are reminded of lesson 12 and their action plan
14	C	- Experiences and actions	Students discuss (perceived) barriers and solutions to overcome these barriers.
15	C	- Implementation intentions	If-then statements are made to help students to overcome (perceived) barriers.

### Step 5: Implementation plan

In IM step 5 program developers set objectives for program adoption, implementation and maintenance and link these objectives to theoretical methods and practical applications for promoting adoption and implementation. In other words: who will do what, and how will this be facilitated.

The school management is an important stakeholder for this intervention. Commitment from the school management is essential to optimize communication with parents and within the school, and with that to optimise the support from the teachers. Moreover, involvement of the school management is important in order to make this program part of the regular curriculum of the school. Therefore, regular meetings with school managements guaranteed proper participation from the schools and improvement the study.

PE teachers organize and prepare strength exercises for their lessons using the workbook, locate and use appropriate (safe) equipment, and if not or not sufficiently available, get (extra) support from the schools management. Finally, they adapt and continue strength exercises throughout the school year, implementing the programme with high completeness and fidelity. Project team members visit the schools regularly to provide assistance in case of difficulties, keep the PE teachers motivated and confirm support from the school management.

The performance objectives for the teachers who implement the motivational intervention are: organize, prepare and implement the motivational lessons. To promote implementation with high completeness and fidelity, these teachers participate in one or two training sessions, given by an expert Motivational Interviewing trainer. They are further supported with a written manual, they can ask for help and, again, project team members visit the schools regularly to provide assistance in case of difficulties, reinforce motivation and assure support from the school management.

### Step 6: Evaluation plan

#### Procedure

**Fig. 5:**
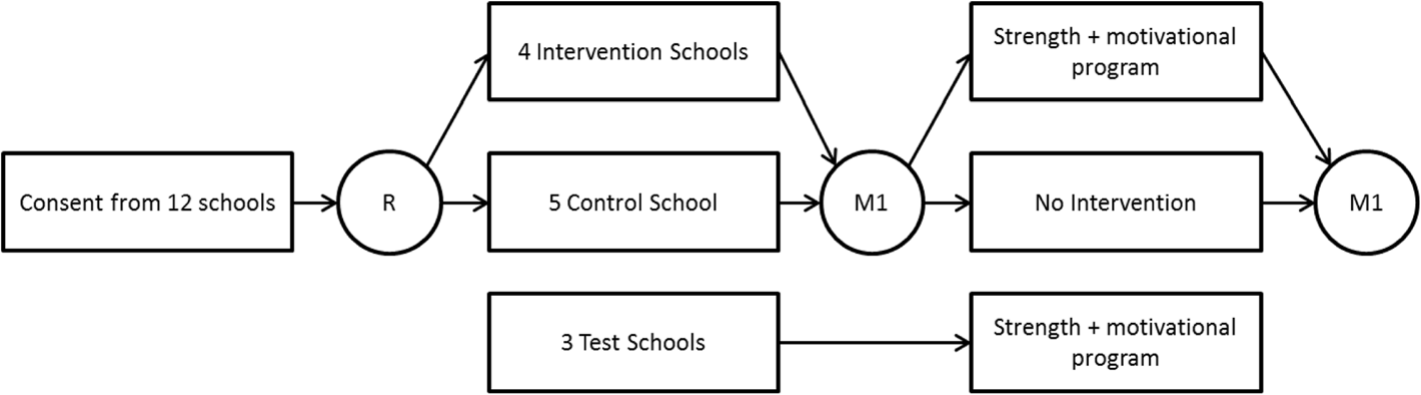
Study design

In 12 high schools, all first grade students of preparatory secondary vocational education participate in a Randomized Controlled Trial (RCT). Schools are recruited via meetings with school managements. Three schools are used for pilot testing the program and its components. The other 9 schools are randomized in an intervention group (4 schools) and a control group (5 schools). In this 12 month RCT, it is the objective that at least 15–30 min of all physical education lessons in the intervention schools contain strength exercises (see also Fig. 5). This intervention, study methods and consent procedure were approved by the Ethical Review Committee of the Faculty of Psychology and Neuroscience, Maastricht University, the Netherlands [ERCPN, dd. 06-09-2015: ECP-04-09-2012; ECP-05-09-2012; ERCPN-04-09-2012A; ERCPN-05-09-2012A1].

After consent from the schools, the parents and their children are informed about the intervention and its measures. At all times, both parents and students are allowed to refuse participation in the measures. After the measures, all students in the intervention group participate in the PE lessons and motivational group lessons as this becomes part of the curriculum. The control group continues with their usual curriculum, without extra emphasis on strength exercises in their PE classes or on physical activity motivation. No motivational lessons are given in the control group. After 12 months, participating students will be included in a post measurement, using the same measurement procedure.

#### Measures

##### Gender and date of birth

Gender and date of birth are provided by the schools’ students administration.

##### Anthropometrics

Anthropometrics are measured using standard procedures (National Health and Nutrition Examination Survey, III, 1988–94. National Center for Health Statistics, [37]). Both height (using the SECA 213 stadiometer) and weight (using the SECA 877 scale) are measured without shoes or heavy clothes to the nearest 1 mm and 0.1 kg respectively. Body Mass Index (BMI) is calculated as weight/height squared (kg/m2) and Z-scores from age- and sex specific reference values. The right arm, right wrist, waist and hip circumferences are recorded to the nearest 0.1 cm using a measuring tape.

##### Body composition

Two methods are used for measuring fat mass and fat-free mass. For the first method, total body water is measured with Deuterium Dilution using a slightly modified Maastricht Protocol [57]. After a baseline urine sample, the students drink 75 mL deuterium enriched water, increasing the deuterium body concentration with 100–150 parts per million. At the end of the school day (a minimum of 4.5 h later), a second urine sample is collected. To calculate total body water, the two urine samples (baseline and enriched) are analysed using isotope ratio mass spectrometry. From total body water, fat-free mass is calculated using age-specific hydration fractions of fat-free mass [30].

For the second measure, skinfold measurements are taken to the nearest 0.1 mm. The thickness of skin folds is measured at four different sites, over the m. biceps brachii, the m. triceps brachii, sub-scapular, and supra-iliacal. From the sum of the four folds, body composition is estimated.

##### Muscle strength

A calibrated and validated [50] *back leg chest dynamometer* (BLCD; Baseline, New York, USA) measures isometric muscle strength, recorded in kilograms (kg) of force. When an external force is applied to a handle, which is attached to an adjustable chain, a steel spring compresses and a pointer moves. For the test, the length of the chain is adjusted to the participants’ height by asking the subject to stand on the base of the BLCD with extended knees. Subsequently, the handle is positioned at the height of the intra-articular space of the knee joint. For the test, participants have to stand on the base, with flexed knees (ca. 30°) and hips while the lower back has to maintain an appropriate lordotic curve. Subjects are asked to lift with a continuous vertical motion by extending the knees, hips, and lower back while holding the handle. After demonstration and a familiarization trial, three trials of which each trial last for circa 3 s is performed, with rest periods of 30 s between trials. Maximal strength attained over the three trials is used for further analysis.

Dominant and non-dominant handgrip strength is measured using the Jamar hydraulic hand dynamometer [14]. Isometric HGS is measured according to the American Society of Hand Therapists. In short, the participants sit in a chair without arm rests. The shoulder remains at 0° flexion, abduction and rotation, the elbow is flexed at 90° and wrist is positioned between 0° and 30° dorsiflexion and between 0° and 15° of ulnar deviation. First, a demonstration and a familiarization trial are given for each arm. Then, the participants are instructed to continuously squeeze for 3–5 s for three trials, with a 30 s rest period between trials. The maximum value of the three trials is used for further analysis. Testing order (dominant/non-dominant) is balanced. The dominant hand is determined by asking the participants with which hand they write.

##### Aerobic capacity

To measure aerobic capacity, all students perform the shuttle run test [27]. Students run back and forth over a distance of 18 m (officially the distance for a shuttle run test is 20 m, but because not all schools have a 20 m gym court, this test is taken over 18 m – therefore, comparisons within this study are valid, but the results cannot be compared with other studies). The running speed is determined by the interval between two sound signals (‘beeps’). Every minute, the speed increases by shortening the interval between two beeps. When a student fails to reach the 18 m-line at the sound signal two times in a row, the test stops for this individual.

##### Physical activity behaviour

Physical activity in daily life is measured by accelerometer (see e.g. [51]). The Actigraph GT3x (Actigraph, Pensacola, FL, USA) triaxial accelerometer is a small device and measures acceleration in three directions (vertical, antero-posterior, and mediolateral). The first grade students were asked to wear the device for five consecutive days, except during sleep and water activities (e.g. taking a shower or swimming). The device is worn on their lower back by using an elastic band. The accelerometer provides activity counts as a composite vector magnitude of the combined three axes as well as time spent in sedentary, light, moderate and vigorous intensity physical activity.

##### Social cognitive determinants

The questionnaire measuring social cognitive determinants consists of three parts. In Table 4 the theory-based concepts are presented with example questions and answering categories. The first part of the questionnaire measures concepts of self-determination theory [10]. Enjoyment is measured with the Physical Activity Enjoyment Scale (PACES), adapted for children [23, 35]. The self-determination concepts are measured with the BrePAC [7], an adaptation for children of the Behavioral Regulation in Exercise Questionnaire (BREQ; [32]) and the Sport Motivation Scale (SMS; [40]). Concepts measured are: enjoyment, intrinsic motivation, identified regulation, introjected regulation, regulation avoidance, and external regulation. Items are rated on a 4-point agree/disagree scale plus a ‘don’t know’-option.

**Table 4:** Questionnaire concepts and example items

Determinant	Items	Example question	Answers and rating
Self-determination: Behavioural Regulation of Physical Activity in Children (BRePAC and PACE)			
Enjoyment (PACES)	16	When I exercise, …I like doing it	Agree/disagree + [don’t know] 5 point scale
intrinsic motivation	4	Why do you participate in exercises? …because it is part of me.	Agree/disagree + [don’t know] 5 point scale
identified regulation	4	Why do you participate in exercises? …because I think it's important.	Agree/disagree + [don’t know] 5 point scale
introjected regulation	5	Why do you participate in exercises? …to show others that I am good at it.	Agree/disagree + [don’t know] 5 point scale
regulation avoidance (a-motivation)	4	Why do you participate in exercises? …so my teacher won't get angry with me.	Agree/disagree + [don’t know] 5 point scale
external regulation	5	Why do you participate in exercises? …because others tell me to do that.	Agree/disagree + [don’t know] 5 point scale
Social comparison concepts			
Performance	2	How good is your performance on strength exercises?	Very bad/Very good, 7-point scale
Social comparison	2	When doing aerobic exercises, I like to compare myself with some who is … than me.	Much worse/much better, 7 point scale
Relative performance	6	My performance on strength exercises is … than most other students.	Much worse/much better, 7 point scale
Preference	3	The next time I would … prefer to do a combination of aerobic & strength exercises.	Very strongly/not at all, 7 point scale
Partner choice	2	Imagine you compete with a partner against two other students. The game has a strength component and an aerobic component. Each of you choses one component. With whom would you prefer to form a team?	a. With someone good in aerobics and not in strength b. With someone good in strength and not in aerobics c. With someone good in both strength and aerobics
Reasoned action concepts			
Attitude	12	Me doing sports is…	Not at all pleasant/very pleasant, 7 point scale
Subjective norm	9	My parents expect me to do strength exercises.	Totally agree/totally disagree, 7 point scale
Descriptive norm	6	Many of my friends do aerobic exercises	Totally agree/totally disagree, 7 point scale
Self-Efficacy	12	If I wanted to, I am confident that I can do sports. Whether I do strength exercises is up to me. If I want to do aerobic exercises, I am confident that I can even when my parents don’t support me.	Totally agree/totally disagree, 7 point scale
Intention	9	The next 3 months, I will do strength exercises regularly	Totally agree/totally disagree, 7 point scale

The second part of the questionnaire measures concepts of social comparison theory [46], especially related to social comparison on two dimensions [28, 49, 53]. The two dimensions for all concepts are strength versus aerobic exercises. Concepts measured are: own performance (good/bad, pleasant/unpleasant, important/unimportant), social comparison (with someone better/worse than me), relative performance (better/worse than most other students, than students with higher weight, than students with lower weight), preference (very much/not at all for strength and aerobic exercises and a combination), choice of partner in an imaginary team performance (someone good in strength/in aerobic/both, someone with more weight/less weight/same weight), see Table 4. Items are rated on 7-point scales or multiple choice answers.

The third part of the questionnaire measures concepts from the Reasoned Action Approach (the successor of the Theory of Planned Behaviour; [15]) and Social Cognitive Theory [2, 20]. The focus in all questions is on doing sports, aerobic exercises, and strength exercises. Concepts measured are attitude (pleasant/unpleasant, important/unimportant, healthy/unhealthy, interesting/boring), injunctive social norm (friends, parents, people who are important to me), descriptive social norm (friends, most students my age), perceived behavioural control and self-efficacy (confident that I can, is under my control, confident even when my parents/friends do not support me), intention (plan to, expect to, will), see Table 4. All items are rated on 7-point scales.

#### Process evaluation

Several steps will be taken to evaluate the implementation process of the intervention. First PE classes are observed on a regular basis to see how the PE teachers implement the strength exercises in their classes. Evaluation questionnaires are used to monitor activities of the PE teachers, the teachers that execute the motivational lessons, and the students. Using an online tool, the progress for the online lessons can be monitored.

#### Power calculation and statistical analyses

Sample size calculations were performed based on the body composition (ratio fat free mass/fat mass) improvements after 12 months for the intervention schools compared to the control schools. With α = 0.05, power = 0.90, and a small to medium effect size (d = .35), 214 participants per group were needed. To test the effectiveness of the intervention, multilevel analyses will be conducted with SPSS. Three levels (student, class, and school) are identified to adjust for clustering of observations within a class or school. Taking the clustering in to account, that we randomized per school, and a drop-out rate of 10-15 %, we aim for a sample size of 600–700 participants.

## Discussion

Intervention Mapping proved to be a useful framework for program planning this school-based program to improve body composition and motivation to exercise in 11–13 year old students by a “Focus on Strength”. Based on a combination of physiological and psychological insights, teachers will integrate strength exercises in their PE lessons and will provide motivational lessons. It is hypothesized that overweight students will find out they are better in strength exercises and all students will get more autonomously motivated to be more physically active outside school. The Intervention Mapping process helped planners to identify who and what should change and to select appropriate behaviour change methods, practical applications and a feasible programme that could be implemented by trained teachers.

In this paper we described the study protocol of the Dutch, school-based program ‘Focus on Strength’ that aims to improve body composition of 11–13 year old students through additional strength exercises, and with that to ultimately improve their quality of life. In this intervention, we focus on youngster’s motivation, we aim to minimize obesity stigma, and we do not aim to focus on weight loss, but on improvements in body composition by encouraging strength training. To make overweight and obese youngsters healthier, stronger, happier, more confident (and feeling better in general), strength exercises may be a fruitful way to go in approaches to promote physical activity among children and adolescents.

## Abbreviations

BLCD, Back-leg-chest dynamometer; BMI, Body mass index; BREQ, Behavioral Regulation in Exercise Questionnaire; HGS, Hand grip strength; IM, Intervention mapping; NCD, Non communicable diseases; NUTRIM, School for Nutrition, Toxicology and Metabolism catalyzes translational research into metabolic and chronic inflammatory disorders; PA, Physical activity; PACES, Physical activity enjoyment scale; PE, Physical Education; PO, Performance objective; RCT, Randomized controlled trial; SMS, Sport motivation scale; ZonMw, Netherlands Organization for Health Research and Development.

## Additional files

Additional file 1 Inspirational Materials for PE teacher. (PDF 4455 kb) Additional file 2 Content of the motivational lessons (Dutch). (PDF 1002 kb)
