# Supplementary material for: The Dutch ‘Focus on Strength’ intervention study protocol: programme design and production, implementation and evaluation plan
Source: BMC Public Health. 2016 Jun 10;16:496. doi: 10.1186/s12889-016-3150-6 (PMC4902907; doi:10.1186/s12889-016-3150-6)

Inspiratieboek

DE KRACHT VAN KRACHT

Kracht als onderdeel van de lessen Lichamelijke Opvoeding.

Versie 1

Januari 2015

Voorwoord

Vanuit de Universiteit Maastricht ontwikkelen we een programma dat zich richt op het promoten van fysieke activiteit van jongeren. Het is een programma voor middelbare scholen, met enerzijds in een deel van de lessen lichamelijke opvoeding aandacht voor krachtinspanningen, anderzijds in een deel van de mentorlessen aandacht voor de motivatie om te bewegen. Voor u ligt het inspiratieboek "DE KRACHT VAN KRACHT - Krachtoefeningen als onderdeel van de lessen Lichamelijke Opvoeding."

In dit inspiratieboek willen we u ideeën geven van krachtoefeningen die u zou kunnen toepassen tijdens uw lessen lichamelijke opvoeding. In eerste instantie geven we u wat achtergrond informatie over het programma, vervolgens richtlijnen waaraan de gymlessen in deze interventie aan moeten voldoen en wat "do's en dont's" als het gaat om krachtinspanningen bij jongeren. Tot slot vindt u in deze handleiding een reeks krachtoefeningen en lesvullende spel- en circuitvormen. Hierin zullen en willen we ook uw ideeën verwerken.

Tot slot is dit inspiratieboek slechts een leidraad die moet leiden tot inspiratie en niet moet worden gezien als een 'dichtgetimmerd programma'. Samen met uw expertise en ervaring hopen we dit boek te kunnen laten groeien.

Gill ten Hoor
Namens het Kracht van Kracht project-team

Inhoudsopgave

Voorwoord	I-2
Achtergrond van het programma	I-6
Waaraan moeten de lessen voldoen?	I-8
“Do’s” en “Don’ts”	I-10
**1. Voorbeelden van lesprogramma’s**	**II**
Circuit- Team performance	
Spellen met Medicine ballen	
Spel: Ravijnen	
Spel: Fat Mat	
Trapezoïde 360◦	
Putje Trek	
**2. Voorbeelden van krachtoefeningen**	**III**
Krachtoefeningen met medicine bal	IV
Krachtoefeningen met gewichten	V
Krachtoefeningen met elastische band	VI
Krachtoefeningen met apparaten	VII
**Notities**	

Achtergrond van het programma

Overgewicht is een groot en groeiend probleem. Hoewel het probleem eenvoudig op te lossen lijkt (minder eten en meer bewegen), is het in de praktijk niet zo simpel. In de eerste plaats moet iemand niet alleen gezonder willen eten en meer willen bewegen, maar moet men ook het gevoel hebben dat ze dat kunnen. Ook wanneer het ‘willen en ‘kunnen’ geen probleem is, kan het zijn dat iemand gewoonweg niet de kennis heeft over wat nu precies goed is en wat niet.

Veel beweegprogramma’s en activiteiten die overgewicht willen voorkomen richten zich vooral op conditie en niet op kracht. Jongeren met overgewicht hebben vaak een minder goede conditie dan jongeren zonder overgewicht, waardoor ze niet gemotiveerd zijn om eraan mee te doen. Wij willen juist op kracht gerichte beweegactiviteiten aanbieden tijdens de gymles waar jongeren met overgewicht goed in zijn, die ze leuk vinden, en die effectief zijn. In het onderzoek combineren we nieuwe inzichten uit de biologie en de psychologie tot één beweegprogramma dat bestaat uit zowel een kracht-gerelateerd als motivationeel deel. Voor jongeren met overgewicht heeft een op kracht gebaseerd programma zowel biologische als psychologische voordelen. Deze jongeren hebben meer lichaamsmassa en daarmee ook meer spiermassa. Hierdoor zijn ze beter in kracht- dan in conditie-gerelateerde inspanningen én sterker dan hun dunnere leeftijdsgenootjes. Deze jongeren zullen beseffen dat ze hier beter in zijn, waardoor ze het leuker vinden en daardoor meer gemotiveerd raken. Door krachttraining zal de verhouding tussen spier en vet verbeteren waardoor ook de stofwisseling verbetert en lange termijn gezondheidseffecten haalbaar zijn.

In dit programma zullen we echter niet de nadruk leggen op overgewicht. Dit programma is voor alle jongeren. Bovenstaande gezondheidsvoordelen gelden ook voor jongeren zonder overgewicht!

Waaraan moeten de lessen voldoen?

Om dit programma te laten slagen (dat wil zeggen: om ervoor te zorgen dat jongeren een betere lichaamssamenstelling krijgen, gezonder worden en over het algemeen meer gaan bewegen) is het belangrijk dat alle lessen minimaal voldoen aan de volgende eisen:

1. Krachtcomponenten. In minimaal één les lichamelijke opvoeding per week moeten krachtinspanningen een grote rol spelen.

2. De motivatie om te bewegen moet worden verhoogd bij alle leerlingen. In alle lessen LO, + de motivationele groepslessen (1x/maand klassikaal, 1x/maand online) moet geprobeerd worden om motiverende gesprekstechnieken te gebruiken waar mogelijk en om alle leerlingen actief bij de les te betrekken.

De krachtcomponenten moeten voldoen aan een aantal voorwaarden:

1. **Veilig.**
2. **Leuk.** Het doen van een kracht-circuit is goed, maar voor sommige leerlingen erg saai. Zorg voor voldoende afwisseling in oefeningen en wissel circuit-lessen af met spel-lessen.
3. **Actief.** Belangrijk dat gedurende de hele gymles zoveel mogelijk leerlingen actief zijn (i.p.v. dat leerlingen op elkaar moeten wachten)
4. **Voldoende intensiteit.** Leerlingen mogen moe worden!
5. **Extra aandacht voor (absolute) kracht.** In dit programma maken we onderscheid tussen absolute en relatieve kracht. Bij absolute kracht werkt de leerling met gewichten of weerstanden. Bij relatieve kracht werkt de leerling met het eigen lichaamsgewicht. In dit programma is het goed om vooral te focussen op absolute kracht. Jongeren met overgewicht zijn in absolute zin sterker dan jongeren zonder overgewicht, maar hebben (bijvoorbeeld) meer moeite met het doen van push ups.

Om de motivatie te verhogen moeten de volgende aspecten in het achterhoofd worden gehouden:

6. **Er mag een competitie-element in zitten.**
7. **Persoonlijke feedback.** In plaats van leerlingen een cijfer te geven op basis van hun prestatie ten opzichte van anderen, is het goed om regelmatig leerlingen ook te wijzen op hun individuele groei.

8. **Zorg ervoor dat de leerlingen elkaars prestaties waarderen. Geef foute/negatieve attributie geen kans.** Wanneer een leerling met overgewicht een prestatie levert, is het belangrijk dat andere leerlingen de prestatie waarderen, en bijvoorbeeld niet zeggen “jij hebt alleen maar gepresteerd omdat je dik bent“

9. **Benut de sociale vergelijking!** Bijvoorbeeld: een team moet worden beoordeeld op het niveau van de beste, en niet op het niveau van de slechtste (dus: als team moet strategisch worden gekeken wie op welk onderdeel wordt ingezet). Zie ook circuit-vormen verder in dit boek.

“Do’s” en “Don’ts”

Op dit moment staan krachtinspanningen bij jongeren in de groei onterecht nog in een kwaad daglicht. De huidige literatuur beschrijft echter dat krachtinspanningen bij jongeren geen schadelijke effecten heeft, maar juist positieve invloed heeft op de gezondheid, en in het bijzonder effectief is bij jongeren met overgewicht. Wel is het erg belangrijk om een aantal aspecten in het achterhoofd te houden om ervoor te zorgen dat leerlingen veilig de krachtinspanningen uitvoeren.

De gouden regels:

.Zorg altijd voor een **goede warming up**.

.Zorg er altijd voor dat leerlingen eerst de **juiste techniek** hebben. Bij het uitvoeren van oefeningen. Daarna kun je pas het gewicht verhogen. Het is makkelijker om met een laag gewicht te beginnen en eerst de juiste (en veilige) techniek aan te leren, dan een foute techniek af te leren.

.Ga **nooit maximaal**. Wanneer een leerling *minder* dan 10 herhalingen goed kan uitvoeren, is het gewicht te zwaar en moet het gewicht verminderd worden.

.Voer oefeningen altijd uit in **minimaal tweetallen.** Hiermee kunnen leerlingen elkaar in de gaten houden en elkaar helpen wanneer nodig. Wanneer leerlingen afwisselend de oefeningen uitvoeren, wordt ook voldoende **rust (minimaal 1 minuut)** gecreëerd tussen de oefeningen.

.Varieer. Zorg ervoor dat verschillende spiergroepen worden gebruikt.

.Eindig de les altijd met een **goede cooling down.**

.Ga per oefening voor maximaal **3x 15 herhalingen**

1

Voorbeelden van lesprogramma's

Circuit – Team performance

Het doel van dit circuit is het winnen van een team challenge. Teams worden willekeurig samengesteld en moeten een circuit afleggen. Het parcours bestaat uit (bijvoorbeeld) 5 onderdelen, deze bestaan voornamelijk uit kracht oefening, maar ook snelheid en conditie oefeningen zijn er in verwerkt. Een aantal voorbeeld circuits zijn hieronder samengesteld. Circuits kunnen natuurlijk ook samengesteld worden uit andere oefeningen of spelelementen.

Voorafgaand het circuit zal er een korte warming up gegeven worden.

Voorbeeld circuit 1

Onderdeel 1: Weerstandselastiek gebonden aan een klimrek, die vervolgens met behulp van de armen zover mogelijk naar de grond toe getrokken moet worden (max. aantal herhalingen in 3 minuten).

Onderdeel 2: Medicine ball 5 keer gooien en hierbij een zo lang mogelijke afstand afleggen. Met behulp van pionnen de afstand bepalen(bv. Pion 1 geeft 1 meter aan, pion 2 1,5 meter etc.).

Onderdeel 3: In hoepels die zigzaggend op de grond geplaatst zijn springen heen en weer zo vaak mogelijk.

Onderdeel 4: Gewichten van 4 kg zo vaak mogelijk heffen in 3 minuten.

Onderdeel 5: Wrist roller van 2 kg zo vaak mogelijk omhoog rollen in 3 minuten.

Voorbeeld circuit 2

Onderdeel 1: Weerstandselastiek gebonden aan de poot van een bank/stoel en deze met behulp van de voet zover mogelijk strekken.

Onderdeel 2: XCO oefening, met gebogen knieën en heupen naar achteren XCO horizontaal vasthouden en dit boven het hoofd bewegen. Hierbij moet de beweging van het grint te horen zijn. Dit moet zo vaak mogelijk gedaan worden.

Onderdeel 3: 100x touwtje springen in zo kort mogelijke tijd

Onderdeel 4: Medicine ball met gestrekte armen horizontaal houden en dit tot aan boven het hoofd met gestrekte armen zo vaak mogelijk tillen in 3 minuten

Onderdeel 5: Vijf gevulde frisdrankflessen (met water) omver schieten met een zachte trefbal.

Voorbeeld circuit 3

Onderdeel 1: Een doos gevuld met bijv. gewichten (iets waardoor het zwaarder wordt) Zover mogelijk over een bepaalde afstand trekken. Afstand wordt met behulp van pionnen bepaald.

Onderdeel 2: Wrist roller van 2kg zo vaak mogelijk omhoog rollen in 3 minuten.

Onderdeel 3: twee leerlingen tegenover elkaar op balance pads. Met een medicinebal zo Vaak mogelijk overgooien in 3 minuten.

Onderdeel 4: Medicine ball zover mogelijk gooien(max. 5 worpen).
Onderdeel 5: Zigzaggend in hoepels springen naar een bak met ballen, een bal pakken en Weer zigzaggend terug om de bal in een lege bak te leggen. Zoveel mogelijk ballen binnen 3 minuten.

Wanneer alle onderdelen door alle teams uitgevoerd zijn, kiest ieder team een speler uit om het op één onderdeel op te nemen tegen een speler van een ander team. De verschillende onderdelen mogen niet door dezelfde leerling uitgevoerd worden. Hierdoor worden ze gestimuleerd om tijdens het uitvoeren van de onderdelen elkaar te beoordelen. Alleen wanneer je als team het beste presteert, kun je de team challenge winnen.

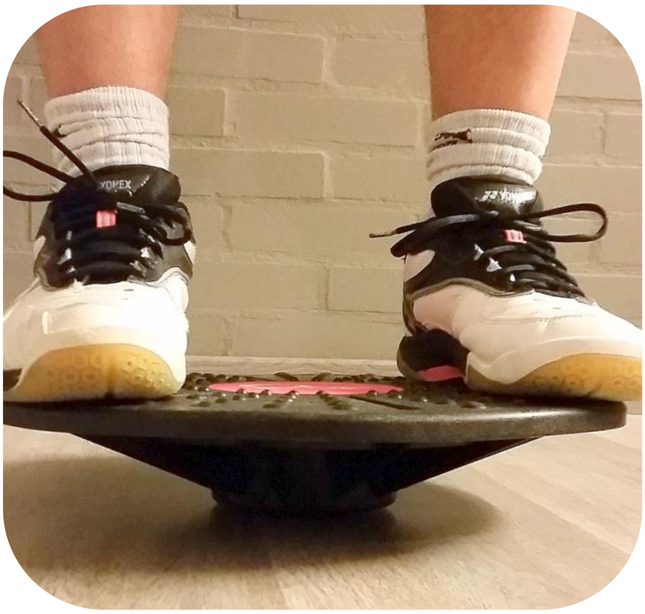

Balance pad

Spellen met Medicineballen

1. Werk in teams van 4-5 leerlingen. Laat telkens één leerling met de medicinebal voor zich (met gestrekte armen) een woord spellen door de medicinebal door de lucht te bewegen. Andere team moet het woord raden

2. Sta met de armen gestrekt voor je lichaam terwijl je een medicinebal vasthoudt. Doe je ogen dicht en laat de medicinebal vallen. Probeer de medicinebal zo dicht mogelijk bij de grond op te vangen

3. Sta met een medicinebal tegen je borst gedrukt. Gooi de bal omhoog, en probeer 180 graden te draaien waarna je de bal weer opvangt. Lukt dit, probeer dan zowel rechtsom, als linksom te draaien. Lukt beide, probeer dan 360 graden te draaien (linksom/rechtsom).

4. Sta met alle leerlingen in een cirkel (ongeveer 1.5m van elkaar af). Aan de ene kant van de cirkel begin je met een bal (de 'kat'), en aan de andere kant van de cirkel begin je met een tweede bal (de 'muis'). De ballen worden bij een startsignaal met de klok mee zo snel mogelijk rond gegooid. De leerlingen moeten proberen ervoor te zorgen dat de kat de muis niet vangt. Variaties op dit spel: voeg een derde bal toe (de 'hond'), of wissel van richting. Je kunt de leerlingen in plaats van de bal naar elkaar te laten gooien (op 1.5m afstand van elkaar), ook de ballen laten doorgeven om de core-strength te verbeteren.

Spel: Ravijnen

Voor het spel ravijnen moet een parcours worden opgebouwd met bijna alle materialen aanwezig in de gymzaal. Een groep leerlingen leg je vervolgens het parcours uit.

Leerlingen moeten zich van onderdeel naar onderdeel verplaatsen als groep, zonder de grond te raken. Ze krijgen hiervoor een rekstok en een touw.

Mogelijke manieren om krachtelementen te verwerken:

1. leerlingen werken in een groep. Één van de leerlingen zit op een bank, en de groep moet deze leerling met bank en al verplaatsen.

2. Voeg het dragen van medicine ballen toe aan het spel. (bijvoorbeeld: voeg de regel toe: de leerling met de medicinebal mag niet lopen. Dit betekend dat de bal continue moet worden doorgegeven om vooruit te kunnen komen).

Spel: Fat Mat

Fat-mat, is met behulp van 1 of 2 grote landingsmatten, verschillende opdrachten laten uitvoeren in groepen.

Bijvoorbeeld:

2 groepen:

. Beide groepen moeten mat optillen waarbij 1 leerling op de mat zit. Groepen moeten in een wedstrijd tegen elkaar een parcour afleggen

. Beide groepen moeten de mat aan de overkant zien te krijgen. Wie het eerste aan de overkant is wint.

. Twee groepen staan allebei aan één kant van de rechtopstaande mat. Als groep moet je proberen de mat aan de andere kant van de gymzaal te krijgen. De andere groep probeert het zelfde in tegenovergestelde richting.

[dit spel kan op vele manieren worden uitgebreid. Nieuwe en uitgebreidere voorbeelden zullen nog verschijnen]

Trapezoïde 360°

Met deze oefening tilt een groep (ca. 8 personen) een trapezoïde op. 1 leerling stapt op de trapezoïde. Vervolgens draait de groep de trapezoïde 360 graden, waarbij de leerling op de trapezoïde moet proberen te blijven. Wanneer de trapezoïde volledig gedraaid is, mag de volgende leerling.

Putje Trek

Bij deze oefening staan circa 10 leerlingen in een kring en houden elkaars handen vast. In het midden van de kring ligt een mat (een denkbeeldige put). Het doel van het spel is om de ander op de mat te krijgen door middel van duwen en trekken. Diegene die op de mat komt is af en verlaat de kring. Uiteindelijk zal er een leerling overblijven die niet op de mat is gekomen.

Soortgelijke oefening: laat leerlingen één tegen één tegenover elkaar staan. Leerlingen houden hun armen omhoog met vlakke hand naar voren, tegen de vlakke hand van de leerling tegenover hen aan. Het doel is om de leerling die tegenover je staat op de grond te krijgen. Let wel: het enige contactoppervlak tussen de twee leerlingen zijn de vlakke handen!

2

Voorbeelden van krachtoefeningen

Krachtoefeningen met medicine bal

Seated Medicine Ball Toss

Procedure

- Zit op de vloer en hou de medicine bal vast (voor tieners maximaal een bal van 3kg).
- Hou de medicine bal met beide handen tegen de borst.
- Gooi de bal zo ver mogelijk.

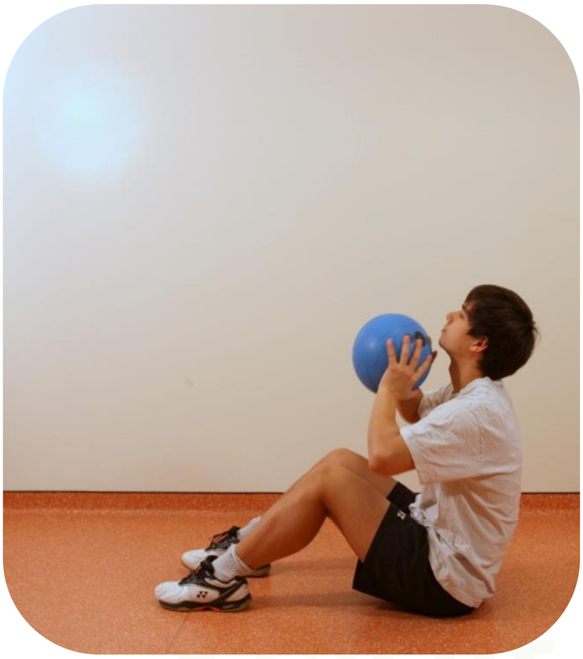

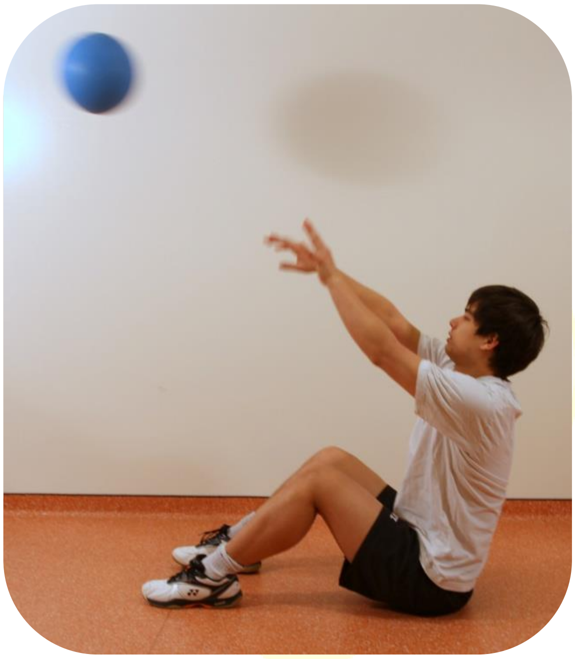

Around The World

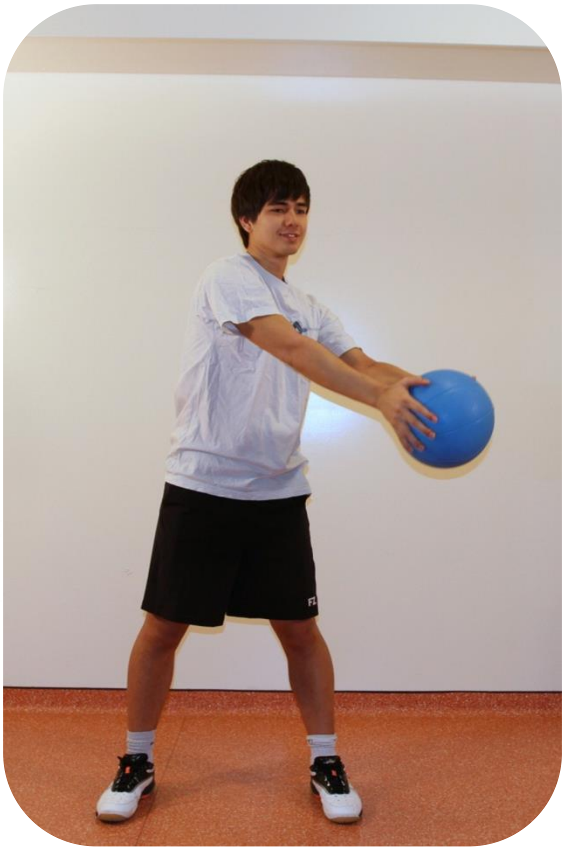

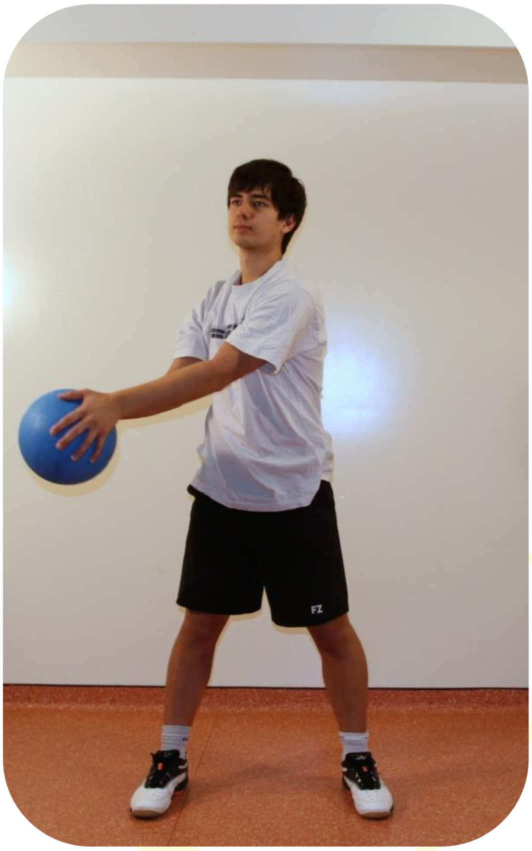

Procedure

- Houd de bal met gestrekte armen vast. Houd de bal boven je hoofd. Je voeten staan ter breedte van je schouders.
- Beweeg de bal in een grote cirkel helemaal rond. Houd je armen hierbij gestrekt.
- Herhaal de beweging en wissel daarna van richting.

Wood Chopper

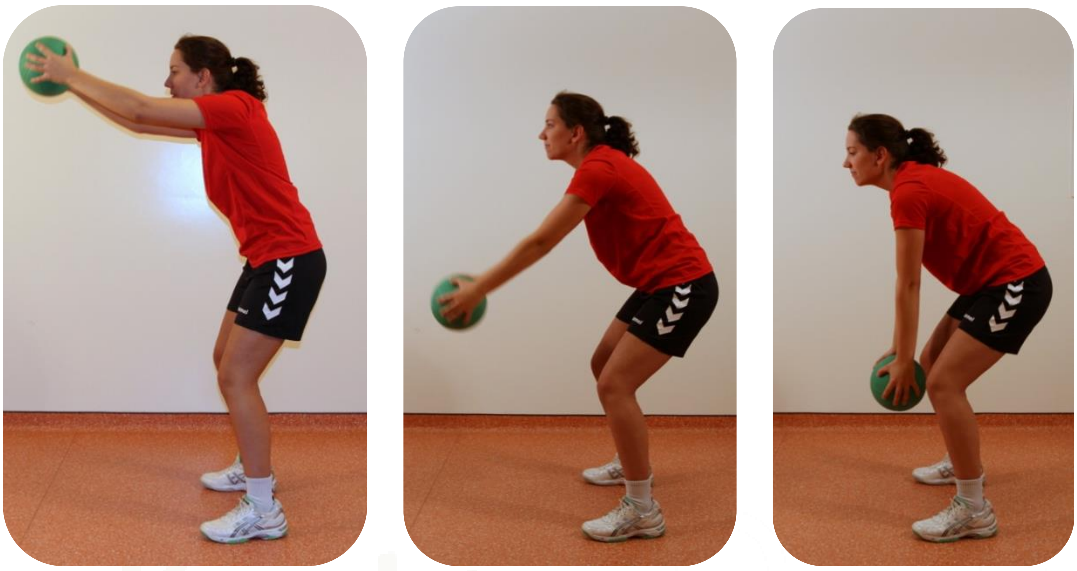

Procedure

- Houd de bal met gestrekte armen vast. Houd de bal boven je hoofd. Je voeten staan ter breedte van je schouders. Je knieën zijn licht gebogen.
- Buig je knieën en heupen verder terwijl je de bal naar beneden tussen je benen zwaait (alsof je hout hakt).
- Herhaal de beweging.

Russian Twist

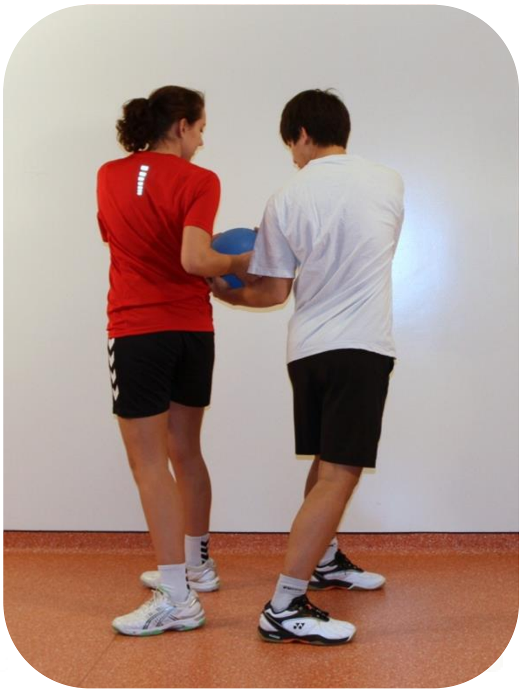

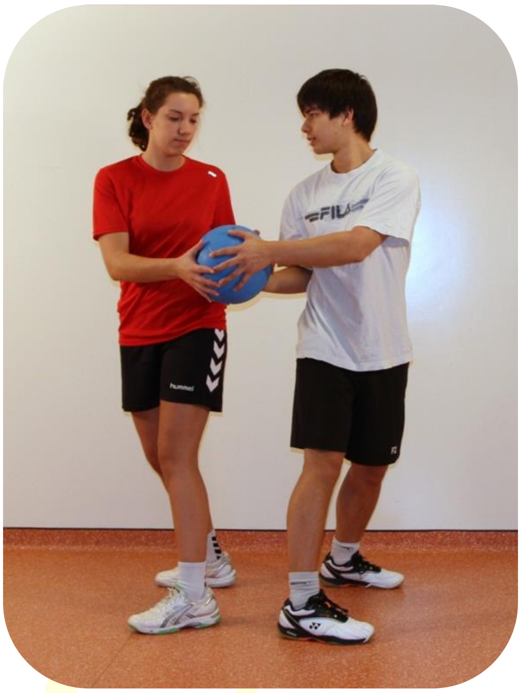

Procedure

- Houd de bal met gestrekte armen vast. Houd de bal voor je lichaam. Je voeten staan ter breedte van je schouders.
- Draai de bal naar links door met je hele bovenlichaam te draaien. Je onderlichaam blijft staan. Je heupen draaien mee. Je voeten kunnen een klein beetje meebewegen zodat je verder kunt draaien. Doe hierna hetzelfde naar rechts.

Knee Lift

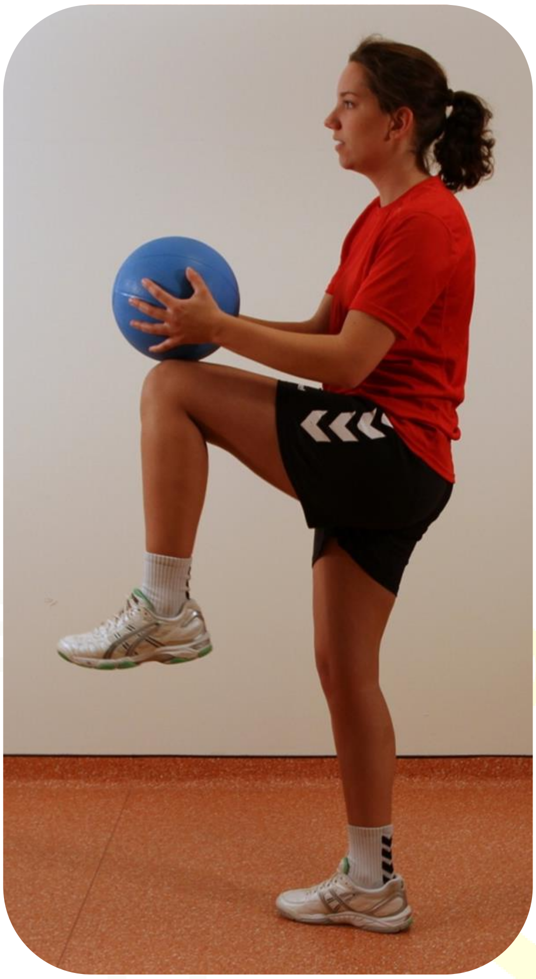

Procedure

- Houd de bal met gebogen armen vast. Houd de bal voor je borst. Je voeten staan ter breedte van je schouders.
- Til je rechter knie op ter hoogte van je heup. Raak de bal aan met je rechter knie. Wissel hierna van been. Herhaal de beweging.

Jog and Catch

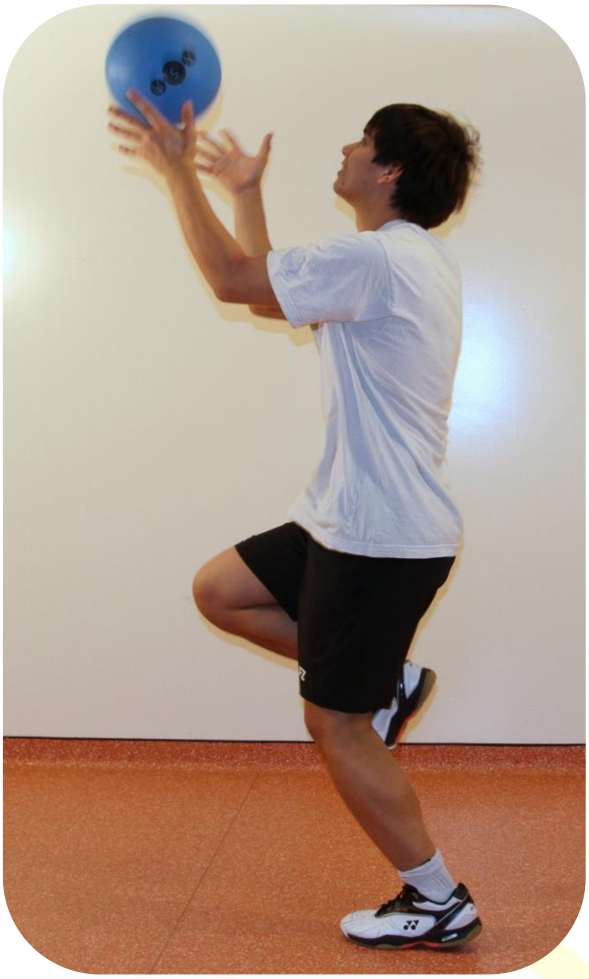

Procedure

- Houd de bal met gebogen armen vast. Houd de bal voor je borst. Je voeten staan ter breedte van je schouders.
- Begin met joggen op de plaats. Terwijl je dit doet gooi je de bal omhoog en vang je hem op.

Tip

- Voor variatie kun je kinderen in hun handen laten klappen als de bal in de lucht is, over laten gooien met een partner of de bal laten gooien van de ene hand naar de andere.
- In plaats van joggen kun je de leerlingen ook vragen om te knie-heffen.

Medicine Ball Front Squat

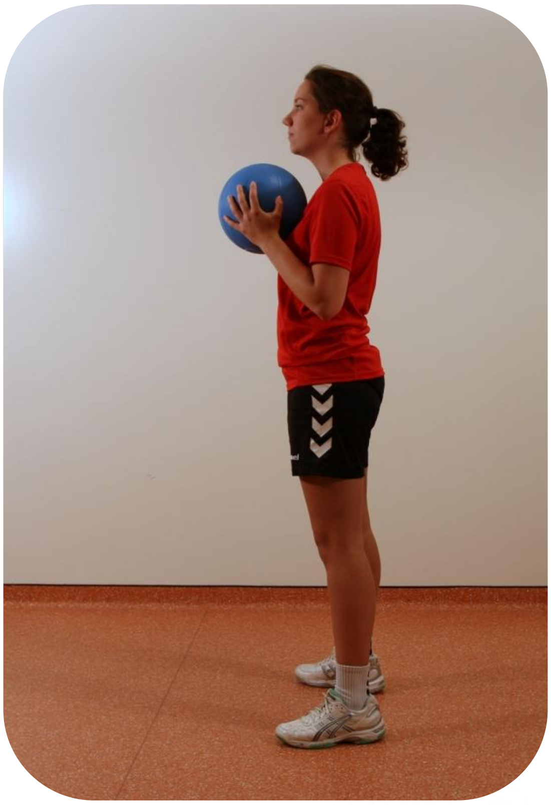

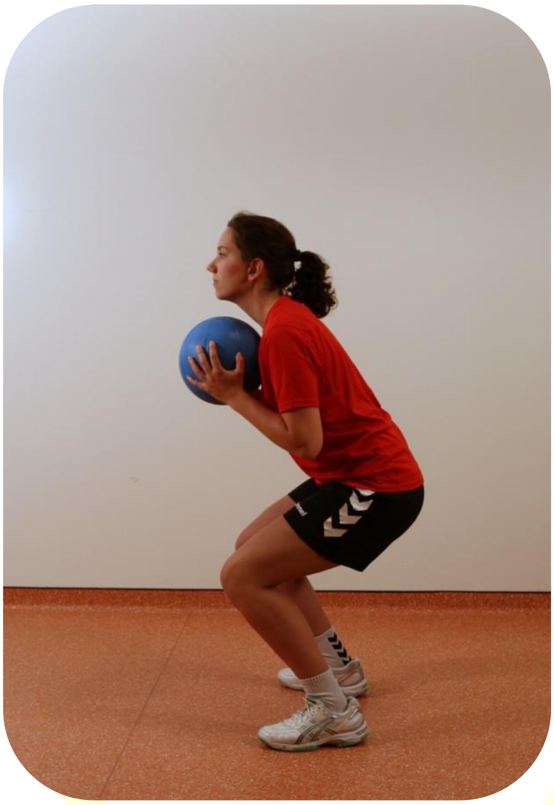

Procedure

- Houd de bal met gebogen armen vast. Houd de bal voor je borst. Je voeten staan ter breedte van je schouders.
- Buig langzaam je enkels, heupen en knieën totdat je bovenbenen parallel zijn met de vloer. Houd je rug recht en je hoofd omhoog.
- Ga weer terug naar de startpositie door langzaam je knieën en heupen te strekken.

Tip

- Probeer niet van beneden naar boven te "stuiteren".
- Leun niet te ver naar voren. Houd je rug recht en je borst vooruit.

Medicine Ball Front Shoulder Raise

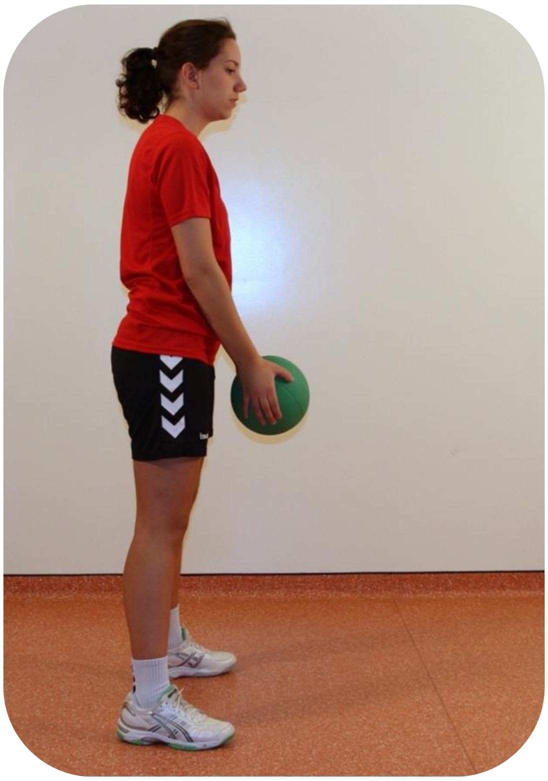

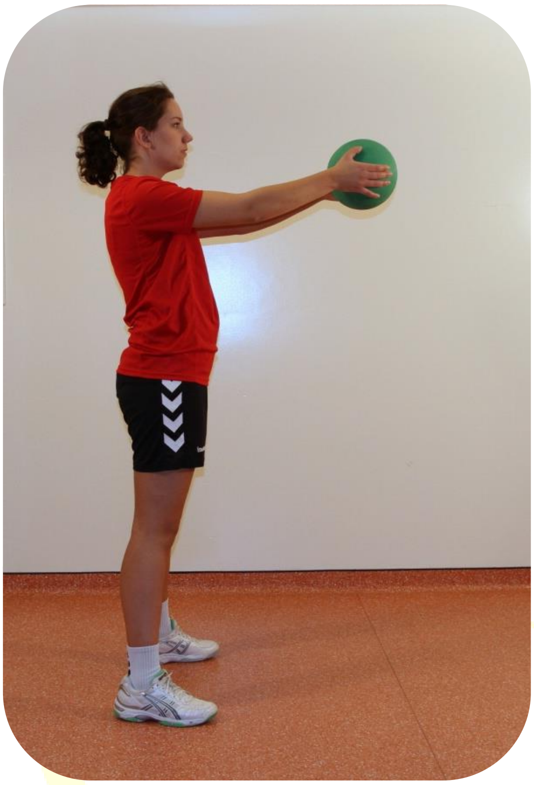

Procedure

- Houd de bal vast met beide handen. Je armen hangen recht naar beneden. Je voeten staan ter breedte van je schouders. Sta rechtop met je rug recht. Kijk recht vooruit.
- Til de bal omhoog met je armen gestrekt.
- Als de bal ter hoogte is van je schouders laat je hem langzaam zakken en herhaal je de oefening.

Tips

- Sta rechtop en houd je onderrug recht door je buikspieren en rugspieren aan te spannen.
- Til je armen niet hoger dan parallel aan de vloer.

Medicine Ball Triceps Press

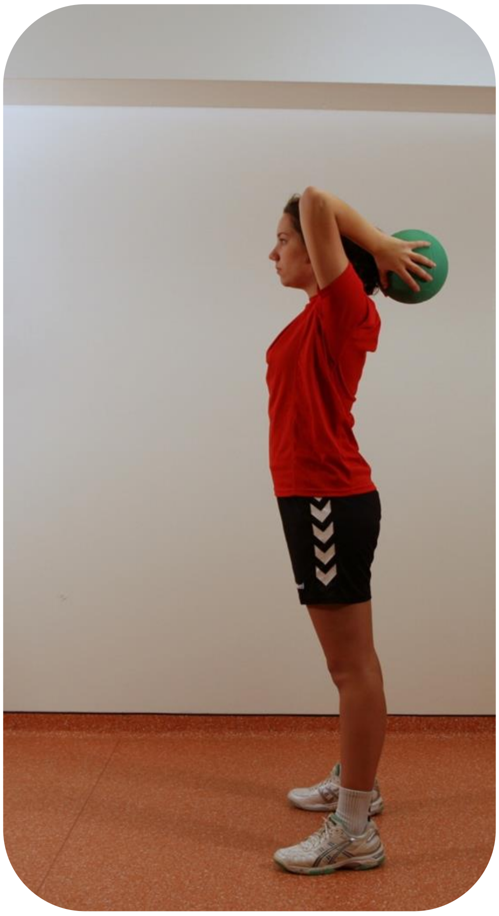

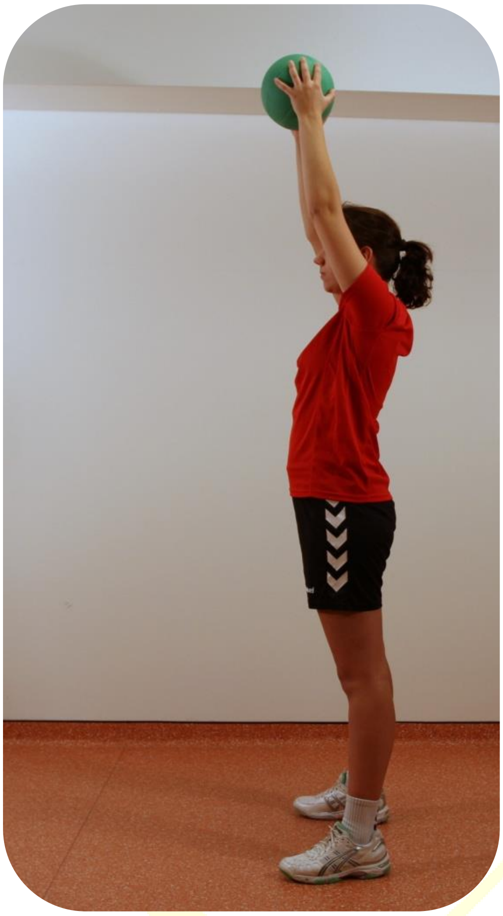

Procedure

- Sta rechtop en houd met beide handen de medicine bal achter je hoofd vast.
- Strek je armen en til zo de bal boven je hoofd.
- Laat de bal weer terugzakken en herhaal de oefening.

Tips

- Alleen de ellebogen en onderarmen mogen bewegen tijdens deze oefening.
- Houd je rug recht door je buikspieren en rugspieren aan te spannen.

Medicine Ball Biceps Curl

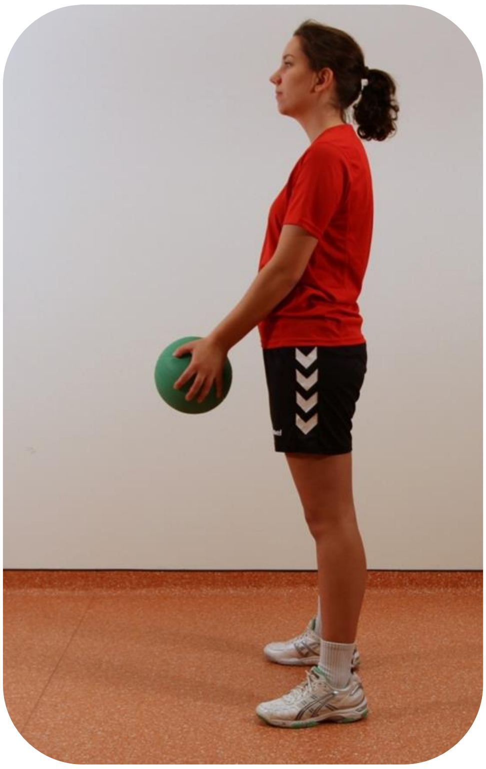

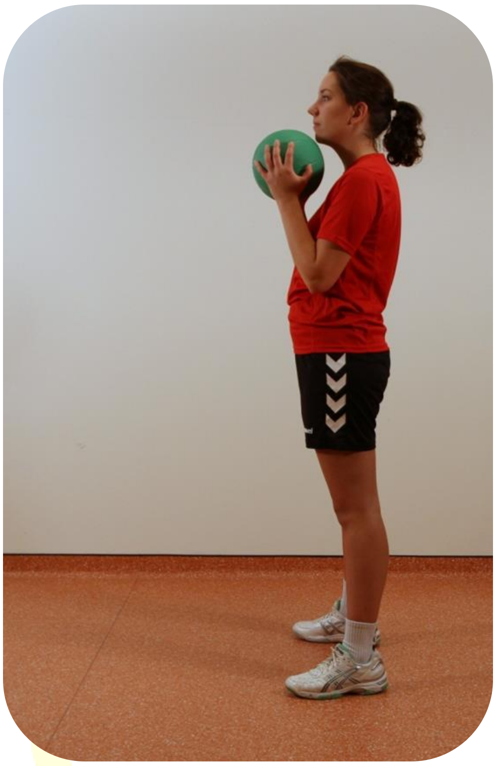

Procedure

- Sta rechtop en houd met beide handen de medicine bal vast. Strek je armen volledig. Deze hangen nu naar beneden.
- Til langzaam de bal richting je borst. Als deze bij de borst is laat je hem weer zakken. Herhaal deze beweging.

Tips

- Sta rechtop met je knieën licht gebogen en je rug recht door de buikspieren en onderrugspieren aan te spannen.
- Als je bovenlichaam toch beweegt kun je tegen een muur staan. Zo sta je zeker weten recht en stil.

Medicine Ball Curl-Up

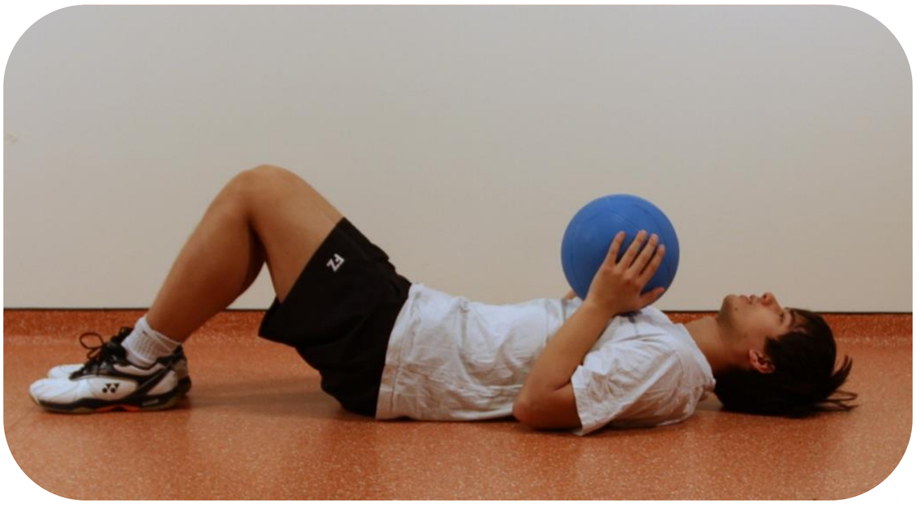

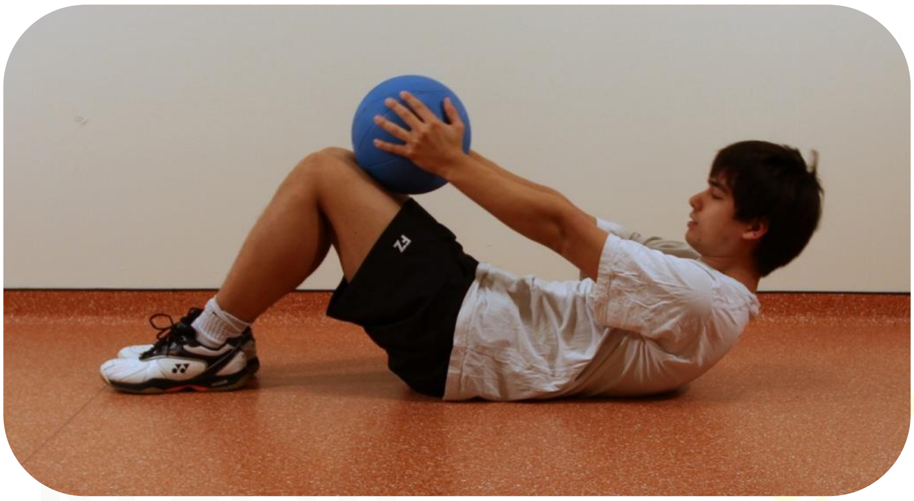

Procedure

- Lig met je rug op de grond en houd de medicine bal vast boven je borst. Je knieën zijn gebogen in een hoek van ongeveer 90 graden.
- Kom met je schouders van de grond af en raak je knieën aan met de bal. Laat je schouders weer zakken en herhaal de beweging.

Tips

- Houd je onderrug in contact met de vloer.

Medicine Ball Twist and Turn

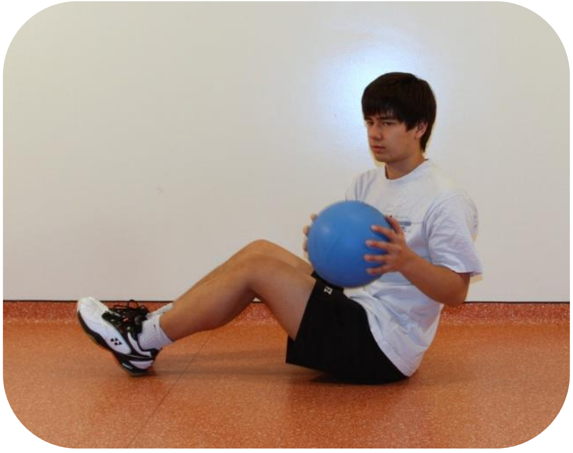

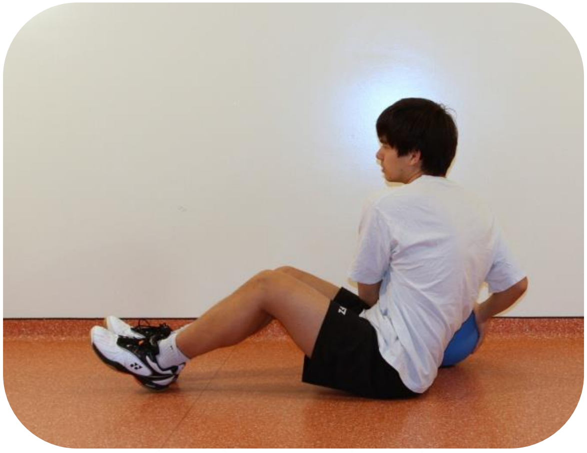

Procedure

- Zit op de vloer met je knieën gebogen in een hoek van ongeveer 45 graden. Je voeten zijn los van de vloer. Houd de medicine bal vast met beide handen. Je houd de bal voor je borst.
- Draai je bovenlichaam van links naar rechts. Je raakt de vloer met de bal aan iedere kant.
- Herhaal de oefening.

Tips

- Leun met je bovenlichaam een beetje naar achter terwijl je draait.

Medicine Ball Biceps Curl

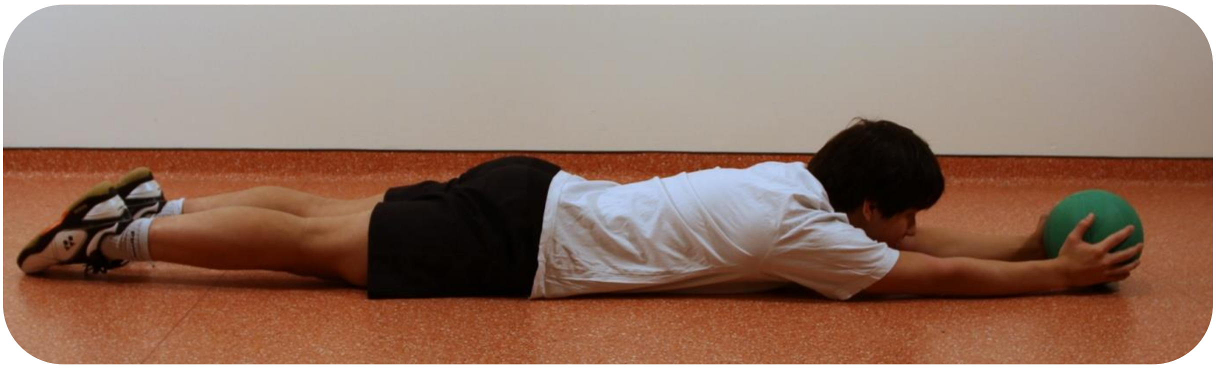

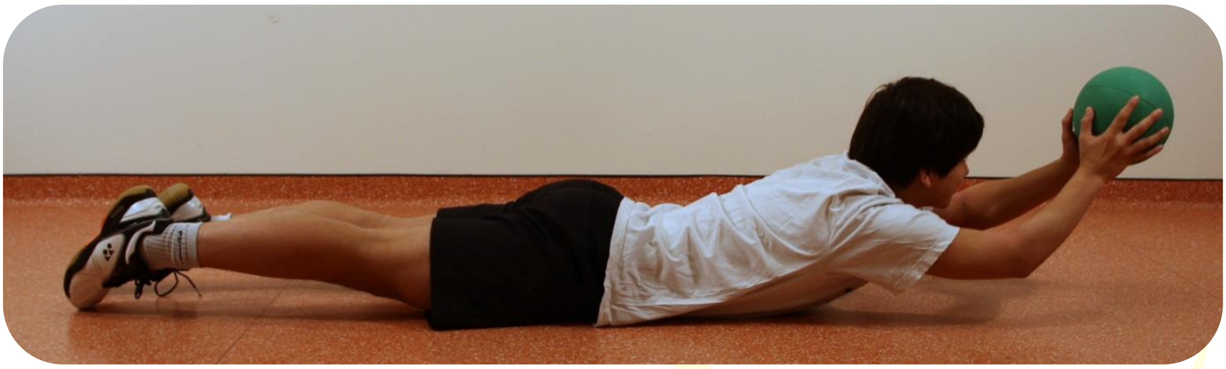

Procedure

- Lig op je buik op de grond. Houd de bal voor je hoofd vast met beide handen.
- Til je bovenlichaam en de bal van de grond. Buig je ellebogen. Houd dit enkele seconden vast en laat je daarna weer zakken.
- Herhaal deze oefening.

Tips

- Probeer normaal te blijven ademen tijdens de oefening.

Medicine Ball Squat Toss

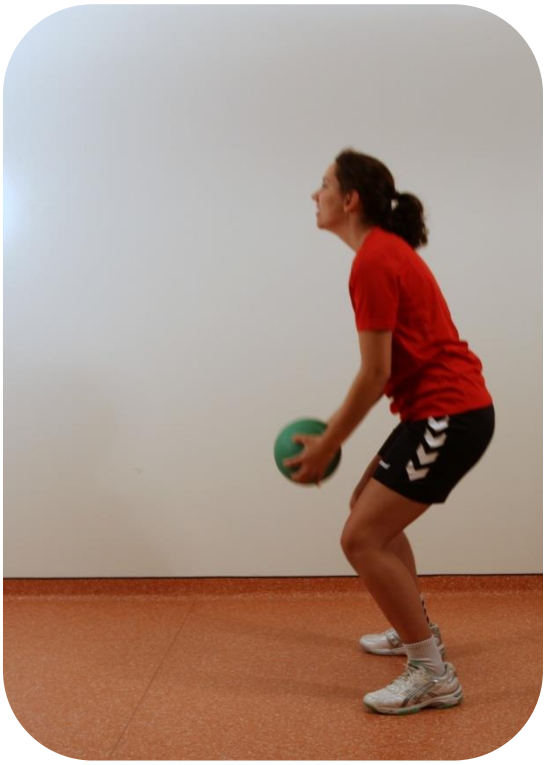

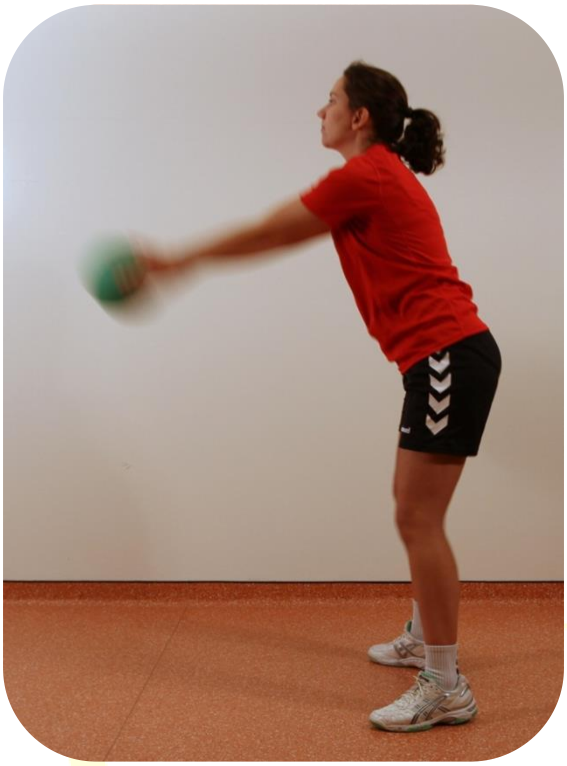

Procedure

- Sta rechtop en houd met beide handen de medicine bal vast. Strek je armen volledig. Deze hangen nu naar beneden. Buig je knieën, enkels en heupen totdat je bovenbenen parallel zijn aan de vloer en houd je rug recht.
- Spring snel omhoog en gooi de bal zo ver mogelijk vooruit.

Tips

- Probeer niet te “stuiteren” tijdens de oefening.

Medicine Ball Chest Pass

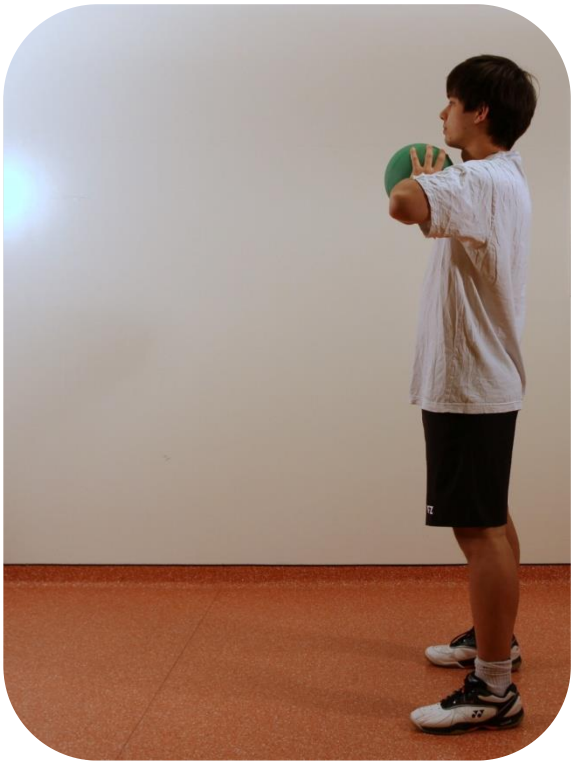

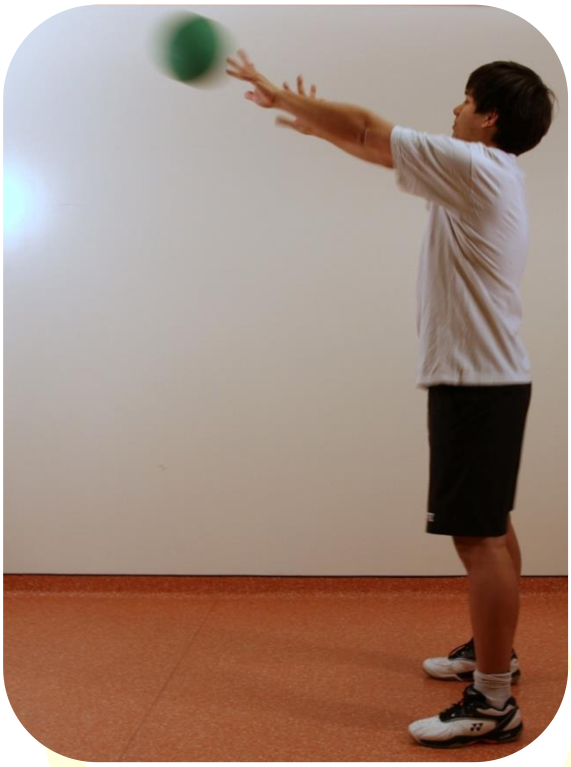

Procedure

- Sta rechtop en houd met beide handen de medicine bal vast. Houd de medicine bal voor je borst.
- Doe een stap naar voren en duw de bal van je borst af. Gooi de bal zo ver als je kunt.

Tips

- Probeer niet naar voren te leunen maar houd je rug recht.
- Je kunt deze oefening met tweetallen doen. In plaats van zo ver mogelijk te gooien gooi je met elkaar over.

Medicine Ball Side Pass

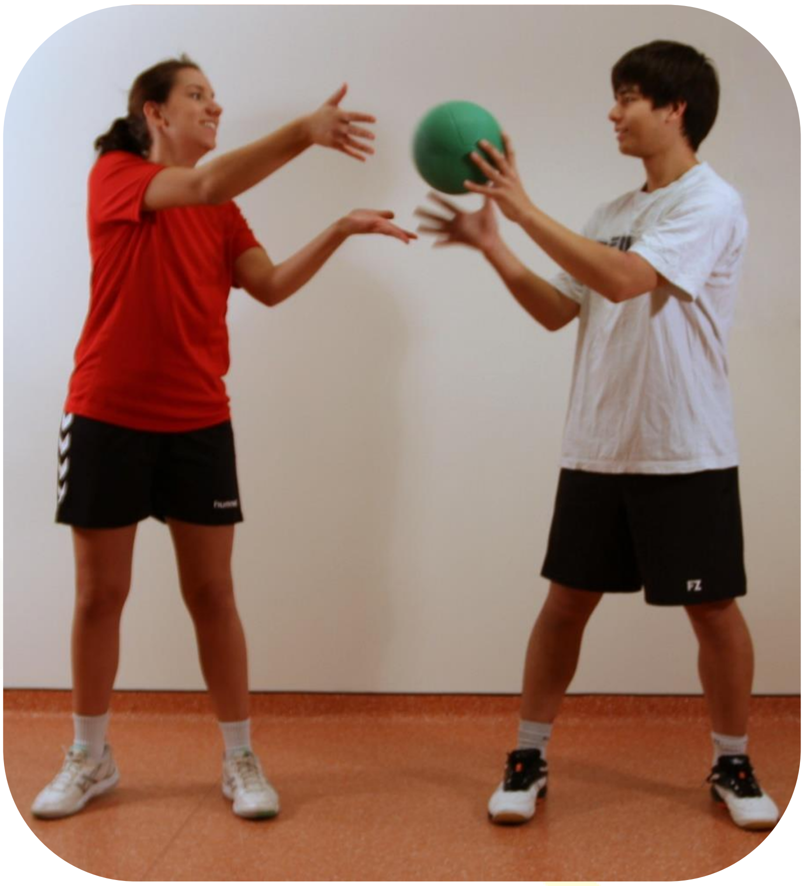

Procedure

- Deze oefening doe je met minstens twee personen. Sta naast elkaar met ongeveer 2 meter tussen jullie in (let op: de afstand op de foto is korter!). Sta rechtop en houd met beide handen de medicine bal vast.
- Gooi de bal naar de ander.

Tips

- Sta rechtop met je knieën licht gebogen en je rug recht door de buikspieren en onderrugspieren aan te spannen.
- Draai je achterste voet mee om een grotere draaicirkel te creëren.

Medicine Ball Overhead Throw

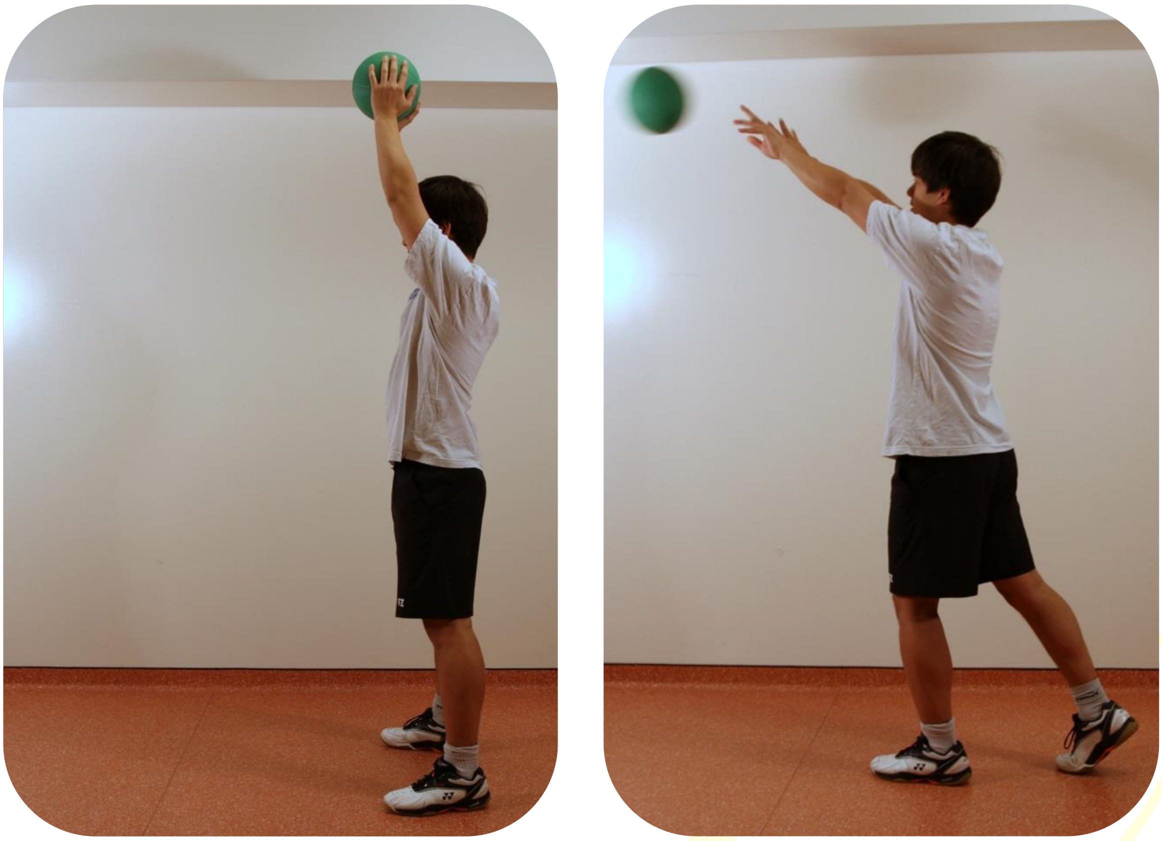

Procedure

- Sta rechtop en houd met beide handen de medicine bal vast. Strek je armen volledig. Houd de bal boven je hoofd.
- Gooi de bal boven je hoofd naar voren.

Tips

- Sta rechtop met je knieën licht gebogen en je rug recht door de buikspieren en onderrugspieren aan te spannen.
- Je kunt deze oefening ook andersom doen. Houd de bal voor je lichaam en gooi de bal boven je hoofd naar achteren.

Gooi de bal tegen de muur als je de oefening alleen doet.

Medicine ball up and over

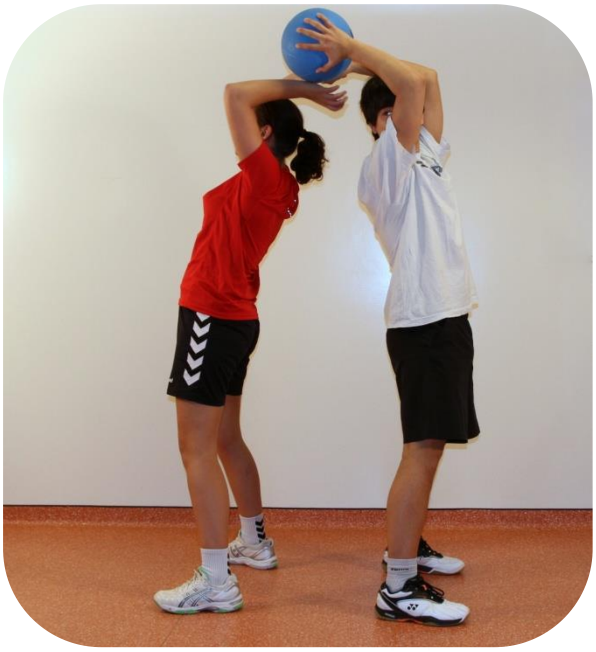

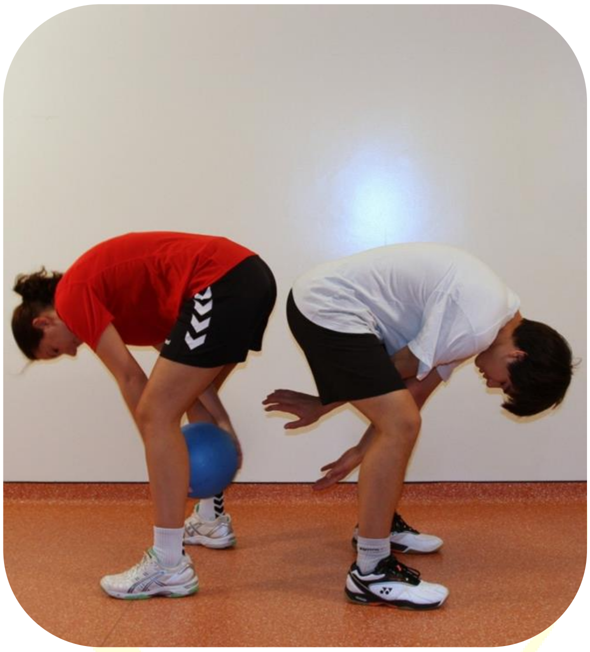

Procedure

- Sta in tweetallen met de rug naar elkaar toe (ongeveer 20-40cm tussenruimte).
- Geef een medicinebal door aan elkaar (a) over je hoofd heen, en (b) door de benen heen.

Krachtoefeningen met Gewichten

Dumbbell Chest Press

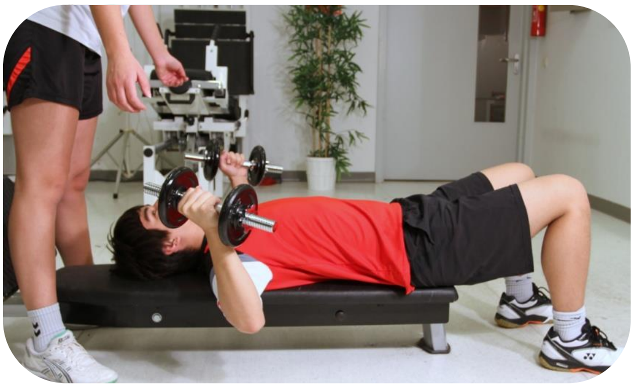

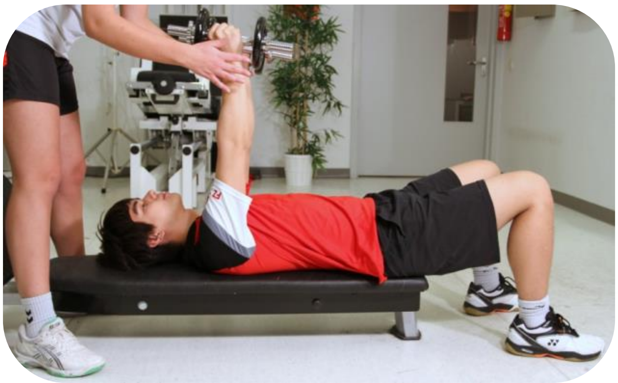

Procedure

- Doe deze oefening in tweetallen (waarbij een de oefening uitvoert en de ander hierbij begeleidt).
- Lig met je rug op de bank en zet je voeten plat op de vloer.
- Hou een halter in iedere hand met je armen naar boven gestrekt.
- Buig langzaam je ellebogen naar buiten toe.
- Duw de halters weer omhoog totdat beide armen weer helemaal gestrekt zijn.

Tips

- Als je voeten de vloer niet raken gebruik een verhoging zodat ze ergens op staan.
- Houd je hoofd, schouders en billen in contact met de bank tijdens de oefening.
- Houd de halters boven je borst in plaats van je gezicht.
- Zorg dat er iemand naast je staat die de beweging kan begeleiden en kan letten op de techniek.
- Voor de variatie kan deze oefening ook worden uitgevoerd met een medicine bal. Ook kan de bank in een hoek van 30-45 graden worden gezet (je ligt dus niet helemaal plat).

Dumbbell Pullover

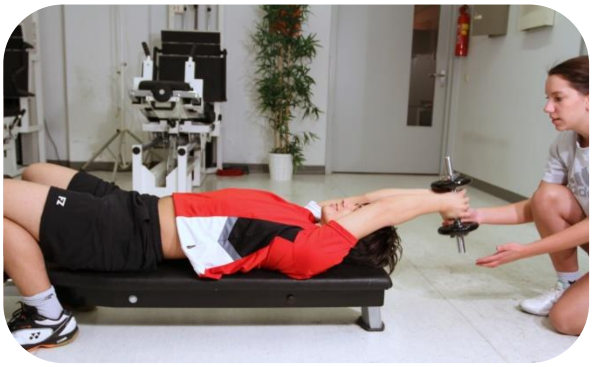

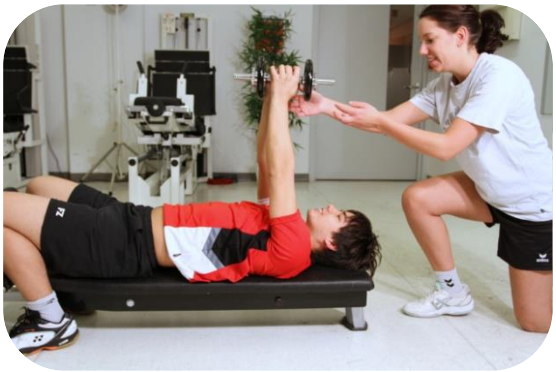

Procedure

- Doe deze oefening in tweetallen (waarbij een de oefening uitvoert en de ander hierbij begeleidt).
- Lig met je rug plat op de bank.
- Strek je armen boven je borst.
- Pak een halter met beide handen vast. Zorg dat je deze goed vast hebt.
- Laat de halter langzaam over je hoofd zo ver mogelijk naar achter zakken. Houd hierbij de ellebogen licht gebogen.
- Ga hierna terug in de startpositie en herhaal deze beweging.

Tips

- De persoon die de oefening begeleidt moet dicht bij het hoofd van de persoon die de oefening uitvoert zitten.
- Omdat het gewicht over het hoofd gaat begin je eerst met een lichter gewicht. Bouw het gewicht langzaam op.
- Doe deze oefening met een vaste halter (in plaats van een halter met losse onderdelen).
- Deze oefening kan ook met een medicine bal uitgevoerd worden.

Dumbbell One-Arm Row

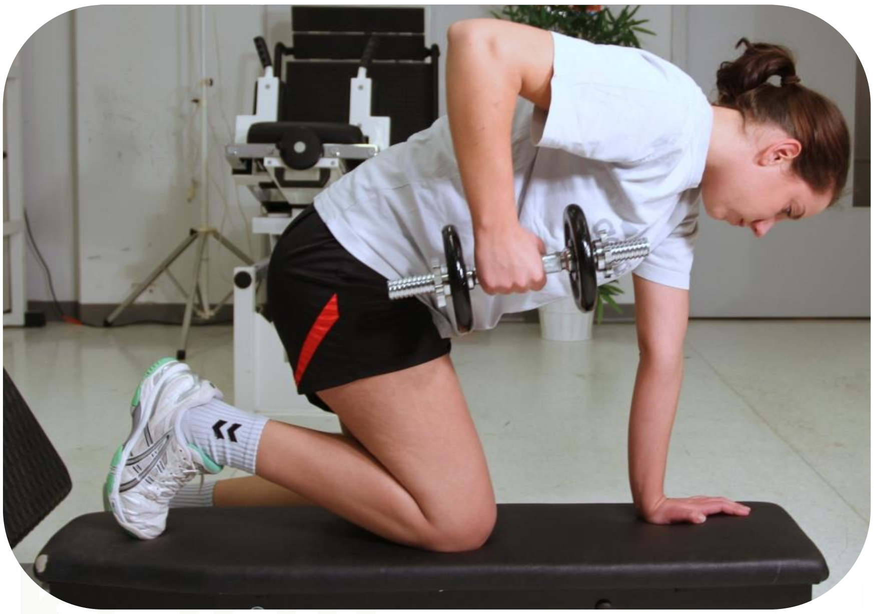

Procedure

- Pak een halter in de rechterhand met de palm van de hand richting het lichaam. Leun met je linkerknie en linkerhand op de bank.
- Laat de halter richting de grond zakken zodat de arm helemaal gestrekt is.
- Til de halter langzaam op richting je borst.
- Laat de halter weer zakken. Herhaal deze beweging.

Tips

- De benen en niet-bewegende arm moeten stevig op de grond en bank staan. De onderrug mag niet draaien tijdens deze oefening.
- Wissel van kant. Houd de halter in de andere hand en plaats de andere knie en hand op de bank.

Dumbbell Upright Row

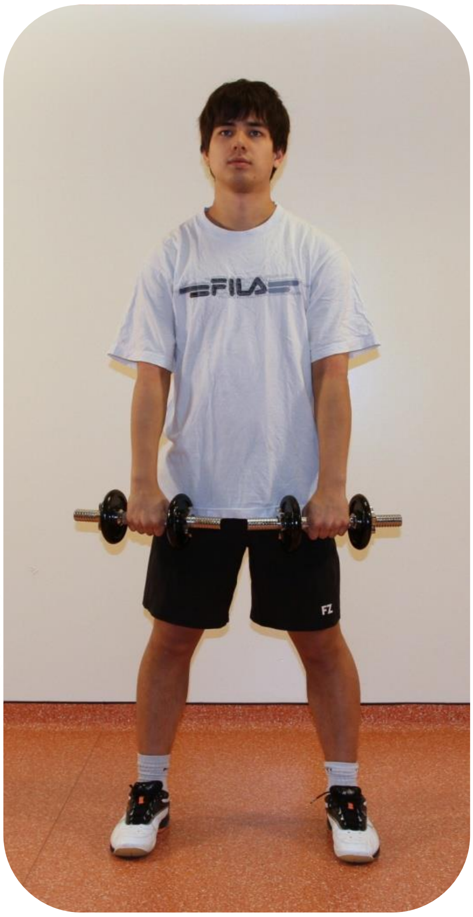

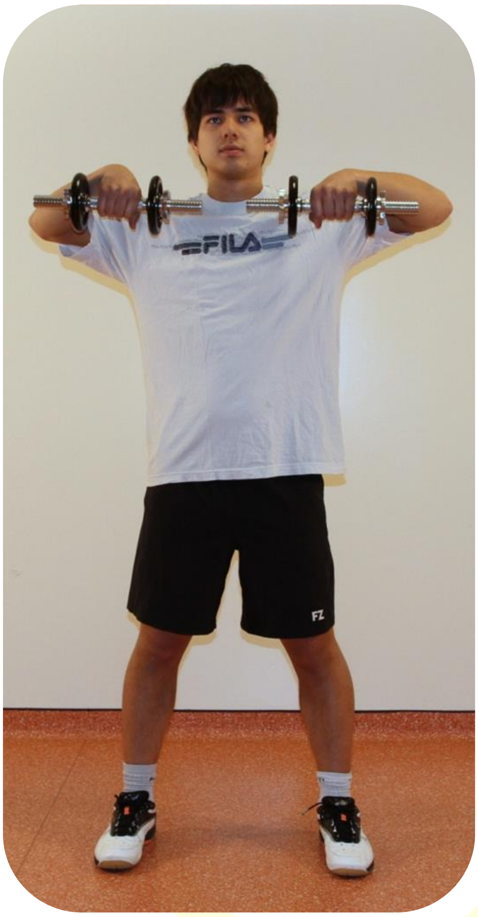

Procedure

- Houd in iedere hand een halter en sta rechtop met de voeten ter hoogte van de heupen.
- De halters hangen voor je lichaam en je handpalmen zijn naar voren gericht.
- Til de halters langzaam omhoog ter hoogte van je borst.
- Laat de halters zakken. Herhaal deze beweging.

Tips

- Sta rechtop en houd de halters dicht bij je lichaam.
- Aan de top van de beweging moeten de ellebogen hoger zijn dan de schouders.

Dumbbell Lateral Raise

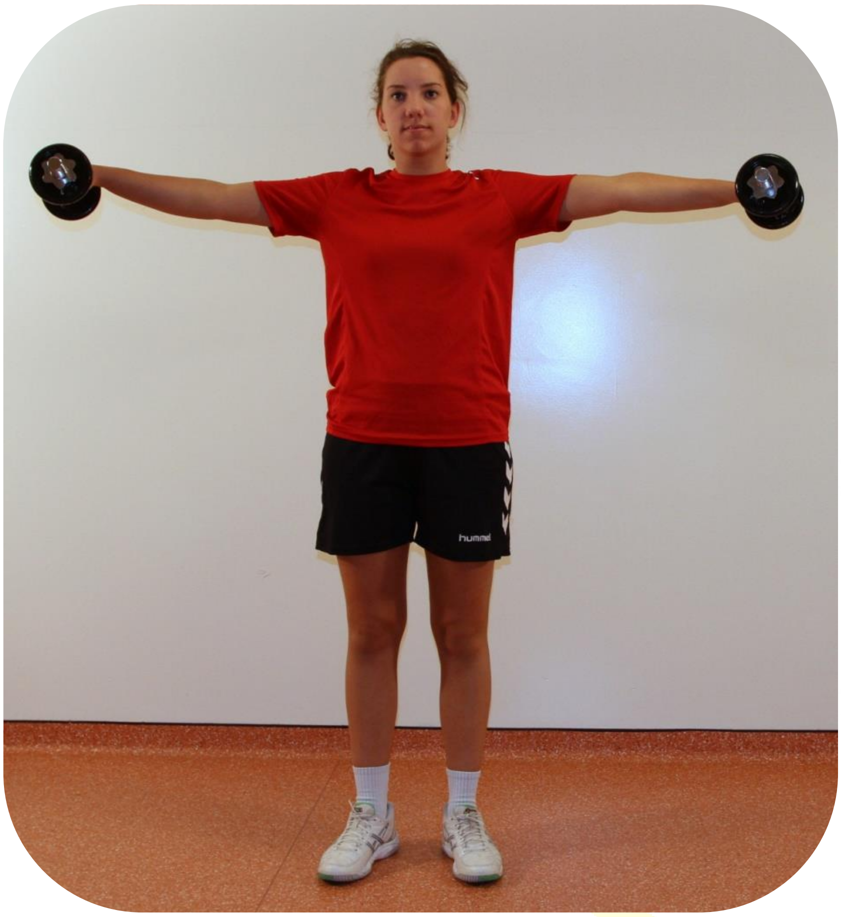

Procedure

- Pak een halter in beide handen met de handpalmen naar het lichaam gericht.
- Sta rechtop, houd je rug recht en de voeten even wijd als de schouders.
- Til de halters langzaam naar boven met de armen gestrekt (de ellebogen zijn hierbij licht gebogen).
- Als de armen parallel zijn aan de vloer laat je de halters weer langzaam zakken.
- Herhaal deze beweging.

Tips

- Houd de rug recht door de buikspieren en onderrugspieren aan te spannen.
- Probeer niet naar achter te leunen.
- Til je armen niet hoger dan je schouders.

Dumbbell Shrug

Procedure

- Sta rechtop en houd in iedere hand een halter. De handpalmen zijn naar je lichaam gericht. De voeten zijn even wijd als de schouders.
- Haal langzaam beide schouders omhoog richting de oren.
- Als ze zo hoog zijn als je kunt laat je ze weer zakken. Herhaal deze beweging.

Tips

- Sta rechtop met je rug recht. Doe dit door je buikspieren en onderrugspieren aan te spannen.
- Buig je ellebogen niet tijdens de oefening.

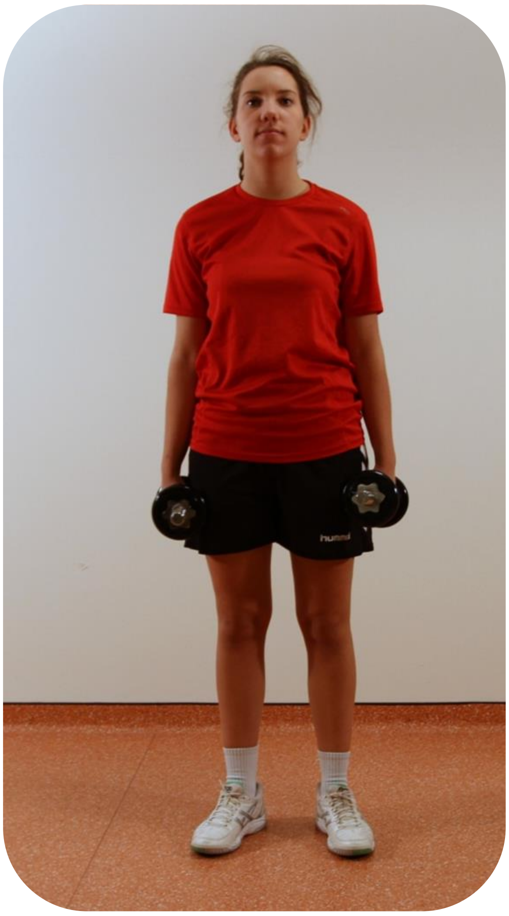

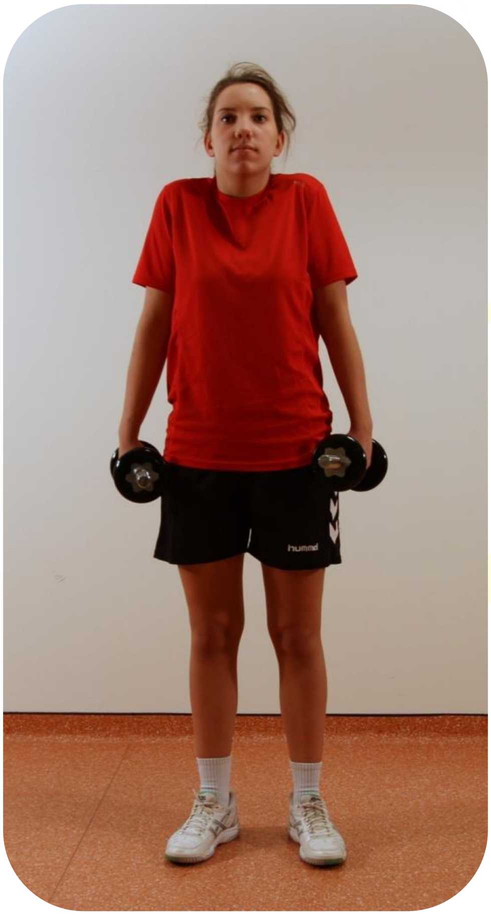

Dumbbell Shoulder Internal Rotation

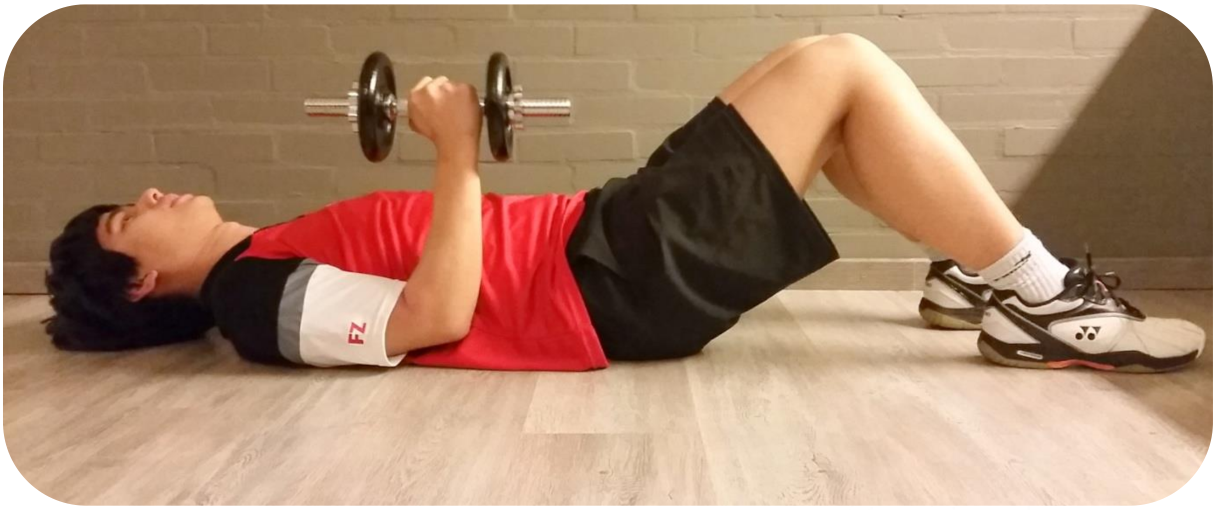

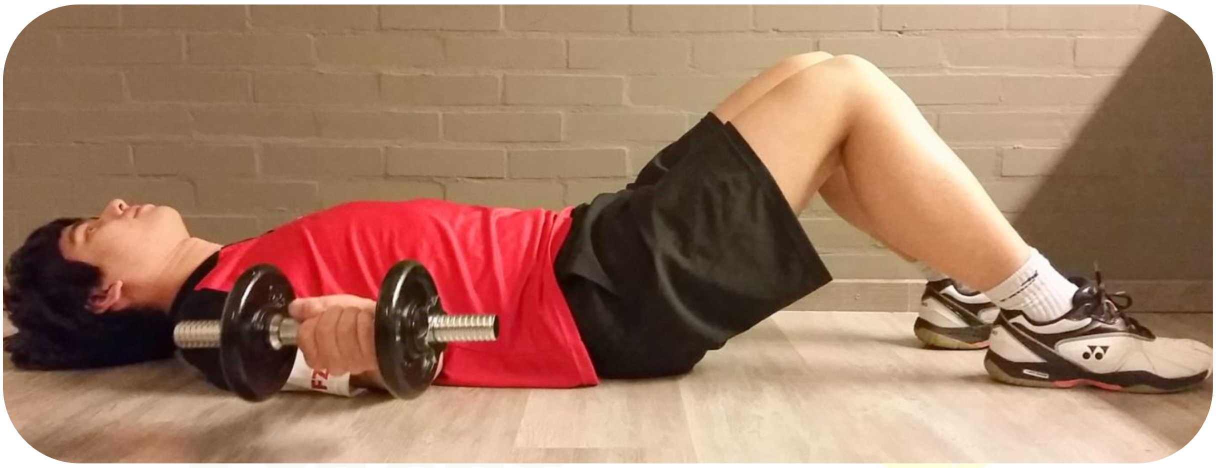

Procedure

- Lig op je rug en houd de halter vast met een hand. Houd de elleboog in een hoek van 90 graden. Houd je bovenarm tegen de grond.
- Laat de halter richting de grond zakken door de schouder te draaien. Ga hierna weer terug naar de startpositie.

Tips

- Dit is een lichtgewicht oefening. Start met een halter van ongeveer 1 of 1,5 kilogram. Verhoog het gewicht met 0,5 kilogram.
- Houd de bovenarm in contact met de grond.

Dumbbell Biceps Curl

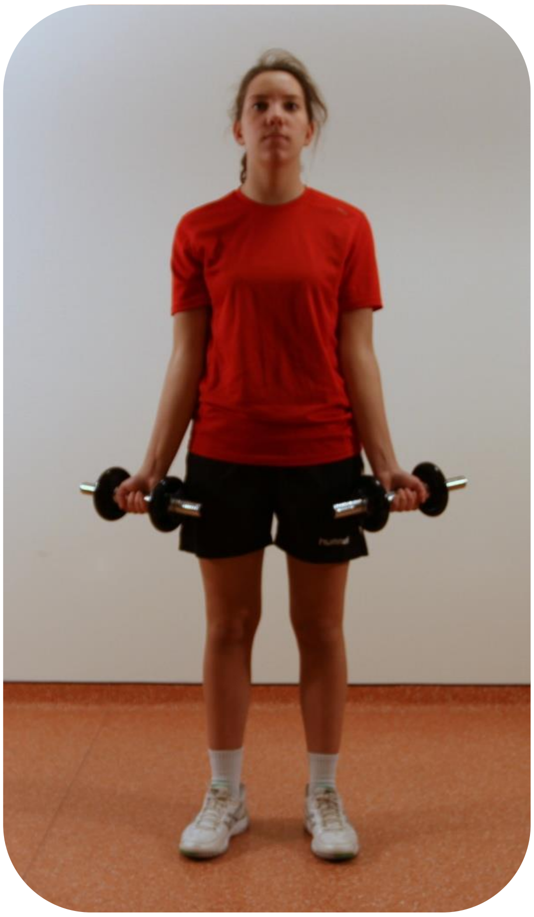

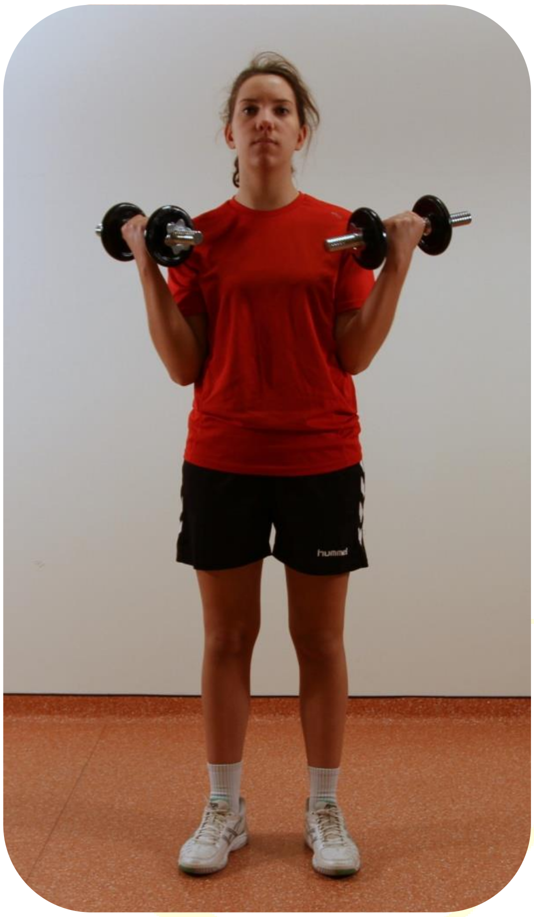

Procedure

- Sta rechtop en houd in iedere hand een halter. Houd de handpalmen naar voren gericht. Strek je armen volledig.
- Til langzaam een halter richting je borst. Als deze bij de borst is laat je hem weer zakken. Herhaal deze beweging.

Tips

- Sta rechtop met je knieën licht gebogen en je rug recht door de buikspieren en onderrugspieren aan te spannen.
- Als je bovenlichaam toch beweegt kun je tegen een muur staan. Zo sta je zeker weten recht en stil.

Dumbbell Hammer Curl

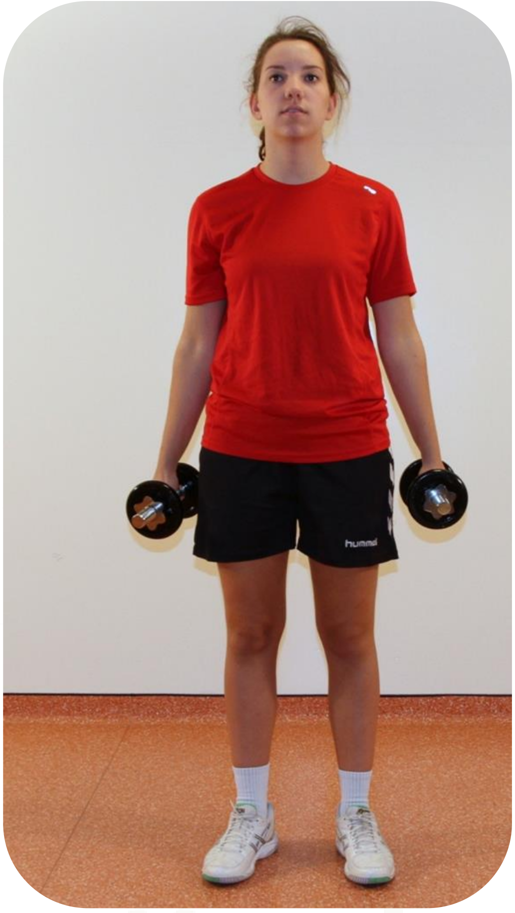

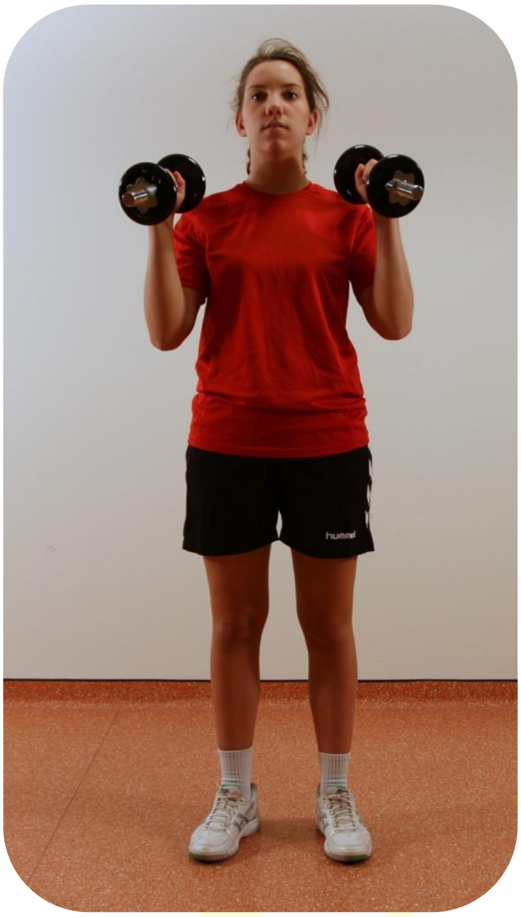

Procedure

- Houd in iedere hand een halter en sta rechtop met je armen gestrekt naast je lichaam en je handpalm naar je lichaam gericht.
- De halters hangen nu ongeveer naast je heupen. De voeten staan ter hoogte van de schouders en de knieën zijn licht gebogen.
- Til de halters langzaam naar je schouders toe door je ellebogen te buigen.
- Zodra de halters ter hoogte van de schouders zijn laat je ze weer zakken door je armen te strekken.
- Herhaal deze beweging.

Tips

- Sta rechtop met je knieën licht gebogen en je rug recht door de buikspieren en onderrugspieren aan te spannen.
- Als je bovenlichaam toch beweegt kun je tegen een muur staan. Zo sta je zeker weten recht en stil.

Dumbbell Triceps Kickback

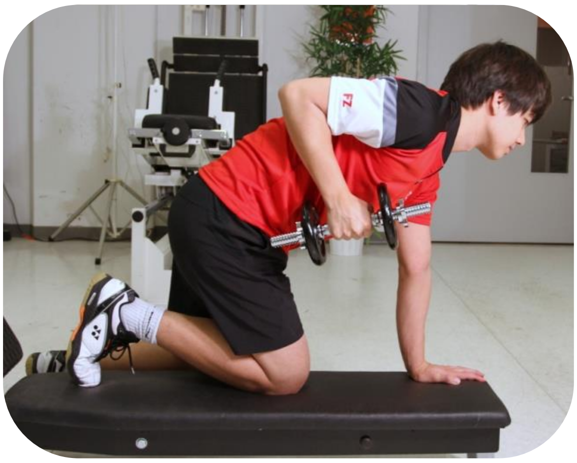
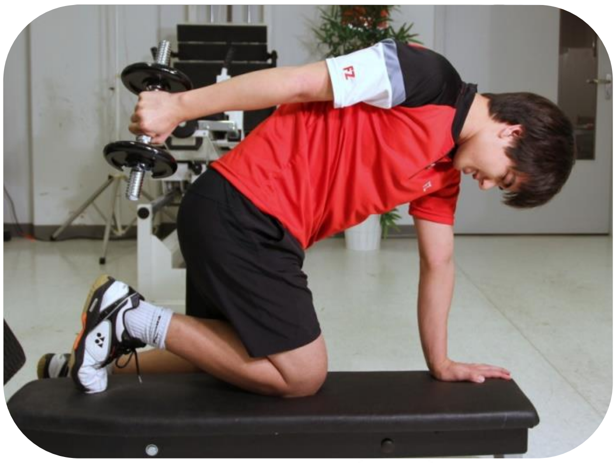

Procedure

- Pak een halter in de rechterhand met de palm van de hand richting het lichaam. Leun met je linkerknie en linkerhand op de bank.
- Houd de elleboog van de arm waarmee de halter wordt vastgehouden gebogen in een hoek van 90 graden. Til de halter naar achter door de arm te strekken.
- Laat de halter weer zakken door je elleboog te buigen. Herhaal deze beweging.

Tips

- De benen en niet-bewegende arm moeten stevig op de grond en bank staan. De onderrug mag niet draaien tijdens deze oefening. Houd de rug helemaal recht.
- Wissel van kant. Houd de halter in de andere hand en plaats de andere knie en hand op de bank.

Dumbbell Wrist Curl

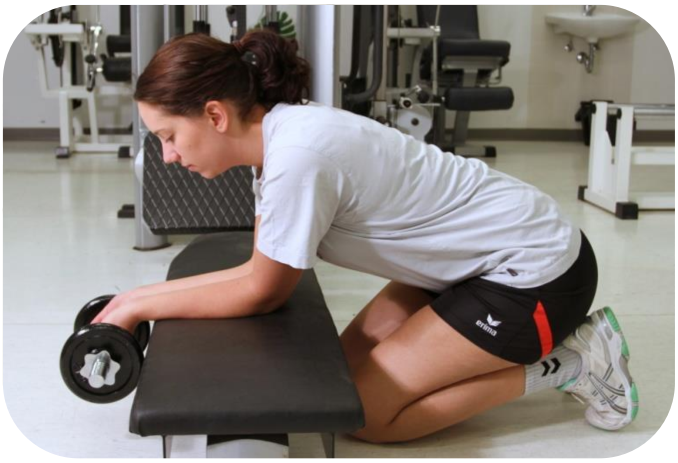

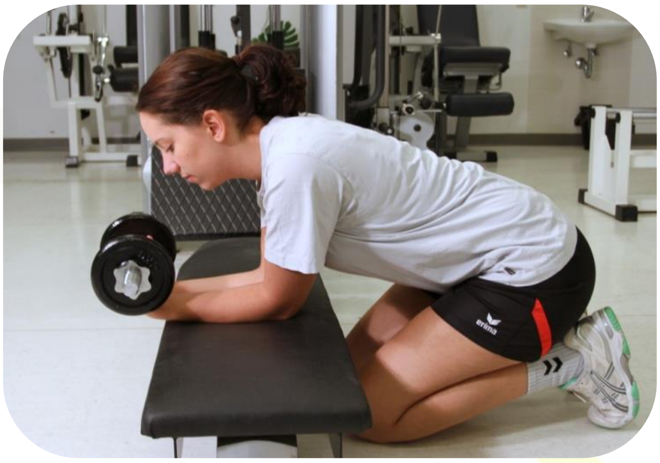

Procedure

- Je zit geknield op de grond en je onderarmen rusten op een bank. Houd in iedere hand een halter vast. Je handpalmen zijn naar boven gericht.
- Buig langzaam je pols richting de grond terwijl je onderarm op de bank blijft liggen.
- Buig hierna je pols weer naar boven (naar je toe). Herhaal deze beweging.

Tips

- De hele onderarm moet in contact blijven met de bank tijdens de oefening. Alleen je pols en vingers mogen bewegen.
- Je kunt deze oefening ook doen met één hand waarna je deze hand afwisselt met de andere.
- Voor de variatie kan het bankje vervangen worden door een grote bal.

Dumbbell Wrist Extension

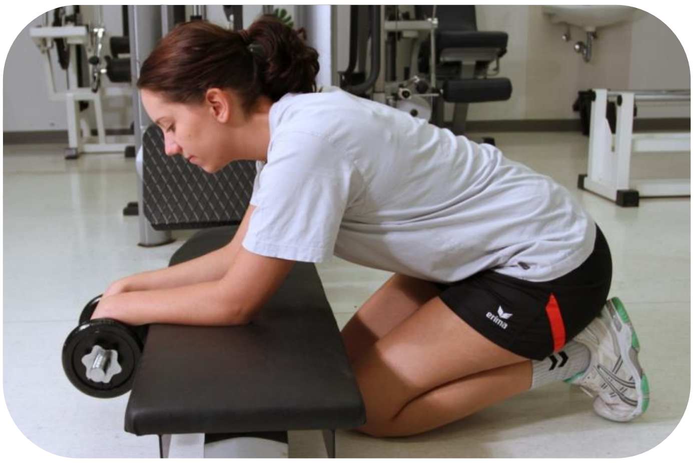

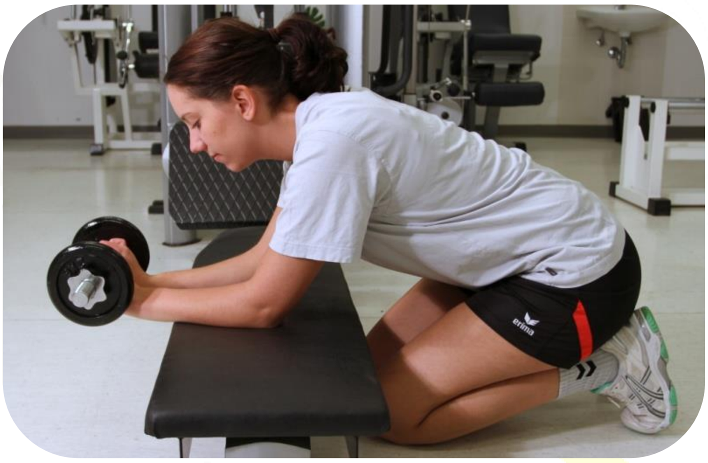

Procedure

- Je zit geknield op de grond en je onderarmen rusten op een bank. Houd in iedere hand een halter vast. Je handpalmen zijn naar de grond gericht.
- Buig langzaam je pols naar boven (naar je toe) terwijl je onderarm op de bank blijft liggen.
- Buig hierna je pols weer naar de grond toe. Herhaal deze beweging.

Tips

- De hele onderarm moet in contact blijven met de bank tijdens de oefening. Alleen je pols en vingers mogen bewegen.
- Je kunt deze oefening ook doen met één hand waarna je deze hand afwisselt met de andere.
- Begin met een licht gewicht omdat deze spiergroep relatief zwak is.
- Voor de variatie kan het bankje vervangen worden door een grote bal.

Wrist Roller

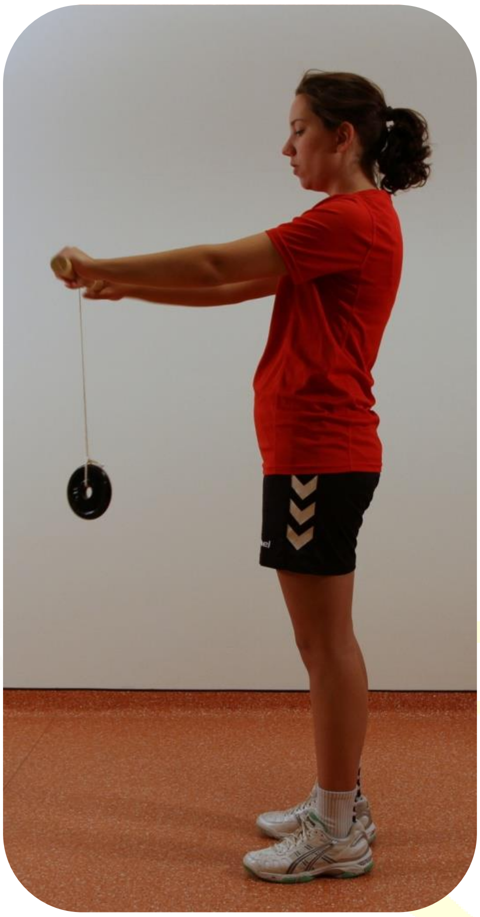

Procedure

- Sta rechtop en houd met beide handen de pols roller vast. De handpalmen zijn hierbij naar de grond gericht. De ellebogen zijn licht gebogen.
- Rol het touw met het gewicht eraan op totdat het helemaal boven is.
- Rol het touw langzaam weer uit.

Tips

- Start met een licht gewicht en bouw dit langzaam op.
- Je kunt variëren door de rol m te draaien. Je kunt dus naar je toe rollen of van je af rollen.

Dumbbell Side Lunge

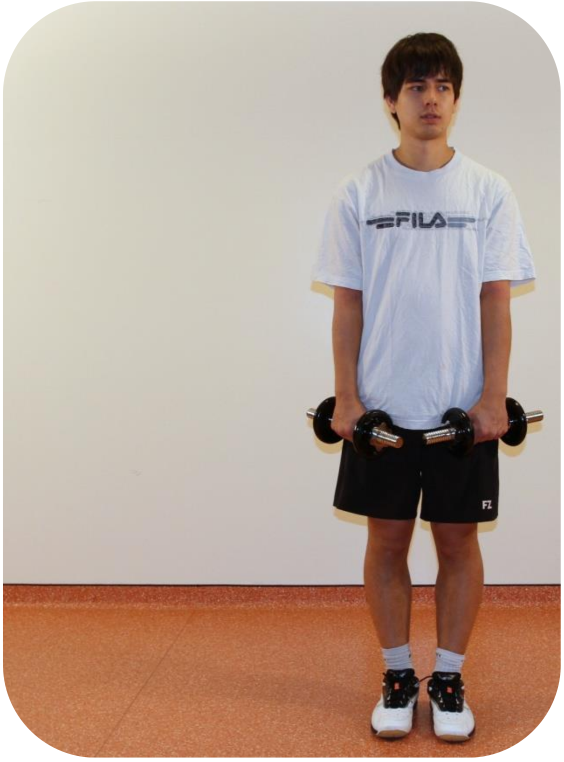

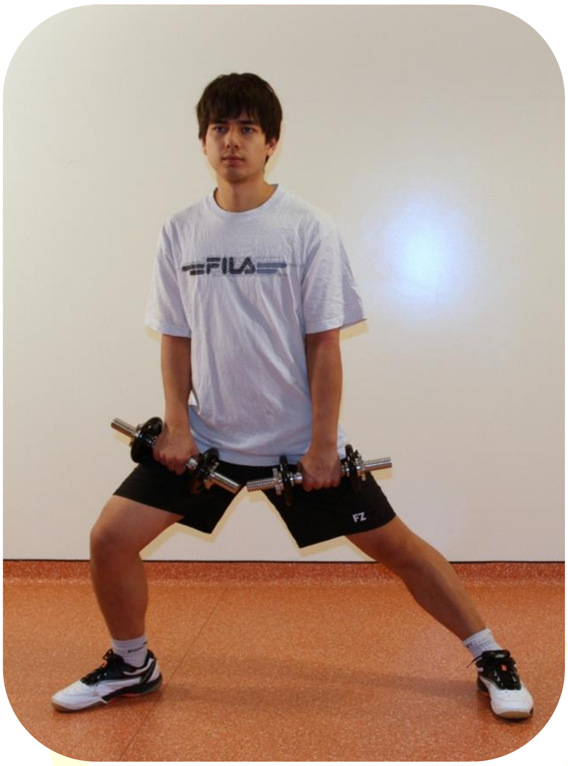

Procedure

- Sta rechtop en houd in beide handen een halter. Deze hangen voor je lichaam. Kijk recht vooruit.
- Neem een grote stap opzij met je rechter knie. Buig de knie van je rechter been en zak met je lichaam omlaag. Dit been moet ongeveer in een hoek van 45 graden zijn met je lichaam.
- Strek je tenen een beetje wanneer je naar buiten stapt.
- Buig je knie zodat je bovenbeen parallel is aan de vloer.
- Ga weer rechtop staan door je af te zetten met je rechterbeen. Als je rechtop staat herhaal je de oefening met het andere been.
- Houd tijdens de oefening je rug recht en je hoofd omhoog.

Tips

- Deze oefening vereist coördinatie en balans. Start daarom eerst zonder gewichten zodat je de techniek goed onder de knie kunt krijgen.
- Let bij het omhoog komen op dat je dit vanuit je benen doet (door goed af te zetten tegen de grond). Probeer dit niet vanuit je bovenlichaam te doen.

Toe Raise

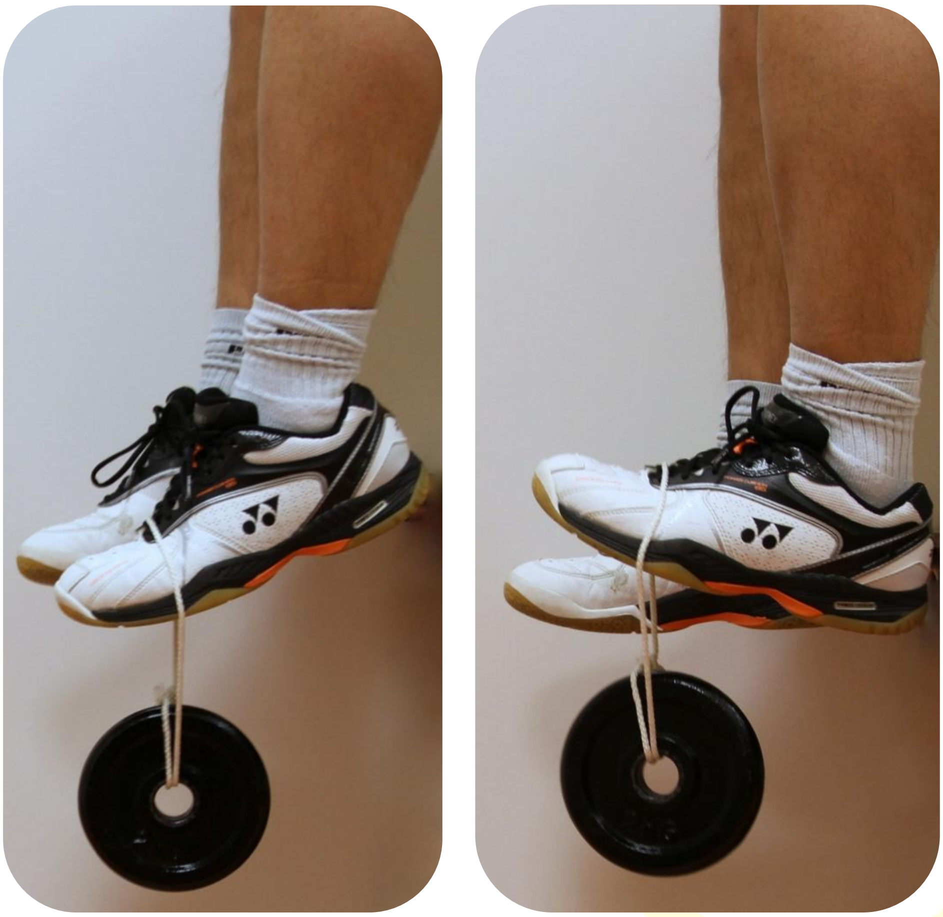

Procedure

- Zit op de rand van een hoge bank en laat je benen hangen. Hang een gewicht aan je voet (bij je tenen). Laat het gewicht los hangen.
- Laat de tenen zo ver mogelijk naar beneden zakken.
- Til het gewicht omhoog door je tenen omhoog te laten wijzen.
- Pauzeer even en laat hierna je tenen weer zakken.

Tips

- De spieren die je met deze oefeningen traint zijn maar klein dus gebruik lichte gewichten.

Krachtoefeningen met elastische band

Elastic Band Leg Curl

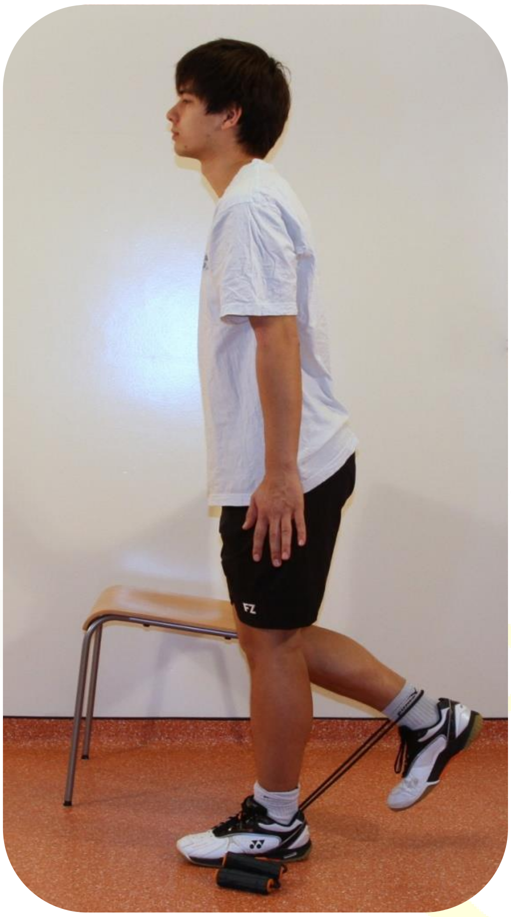

Procedure

- Maak van de elastische band een lus en ga op de uiteinden staan met je linkervoet.
- Je rechtervoet gaat in de lus. De lus zit nu achter je rechter enkel.
- Buig langzaam je rechterbeen naar achteren (richting je billen). Blijf rechtop staan met je lichaam.
- Laat je been weer zakken en herhaal de oefening.
- Wissel van kant.

Tips

- Gebruik om te beginnen een smalle band.
- Gebruik een hand om in balans te blijven. Je kunt ook gebruik maken van een muur of stoel om te balanceren.

Elastic Band Standing Chest Press

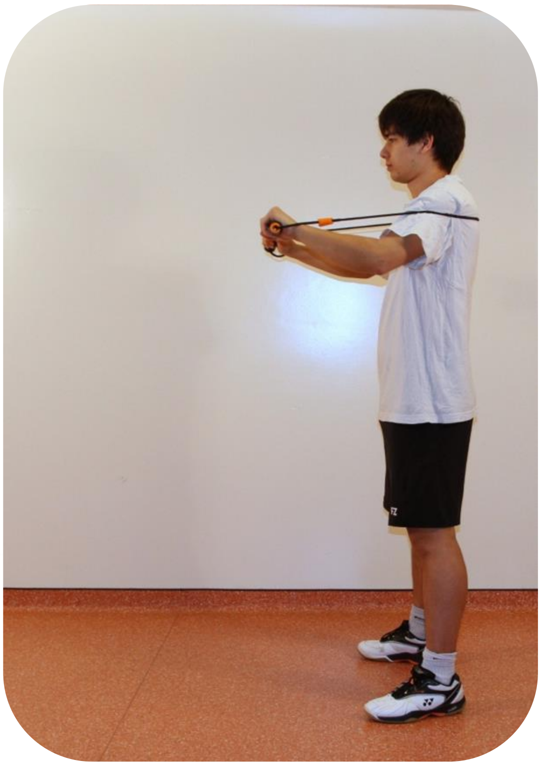

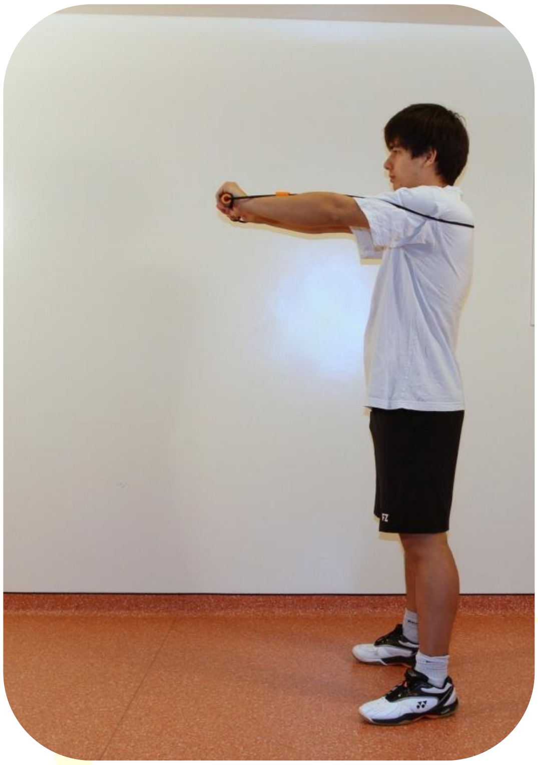

Procedure

- Pak de uiteinden van de band vast met beide handen. De band gaat achter je rug om. Je armen zijn nu gebogen en je houd de band goed vast.
- Strek je armen langzaam.
- Ga weer terug naar de startpositie en herhaal de oefening.

Tips

- Strek beide armen tegelijk en even snel.
- Houd je bovenlichaam recht.

Elastic Band Seated Shoulder Press

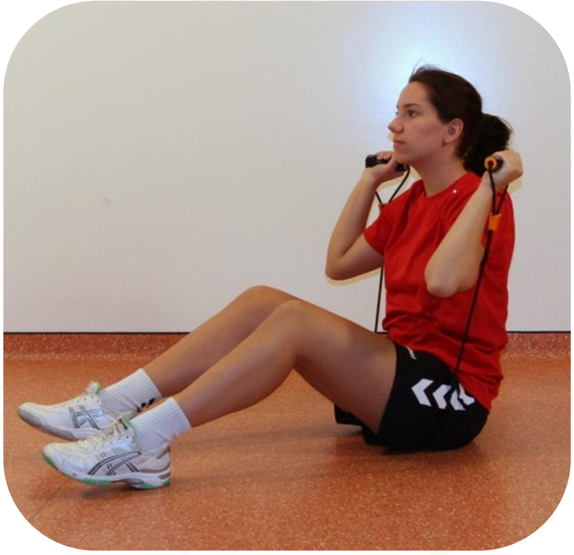
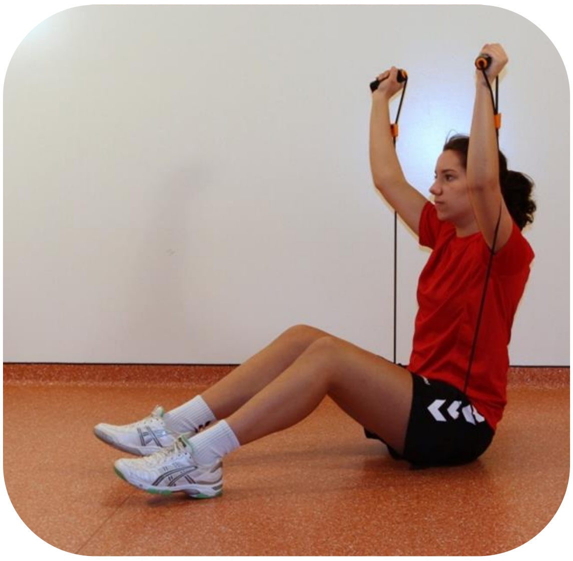

Procedure

- Pak de uiteinden van de band vast met beide handen. Je zit boven op het midden van de band. Je armen zijn nu gebogen en je houd de band goed vast.
- Strek je armen langzaam boven je hoofd.
- Ga weer terug naar de startpositie en herhaal de oefening.

Tips

- Strek beide armen tegelijk en even snel.
- Houd je bovenlichaam recht.
- Strek je ellebogen helemaal boven je schouders.

Elastic Band Upright Row

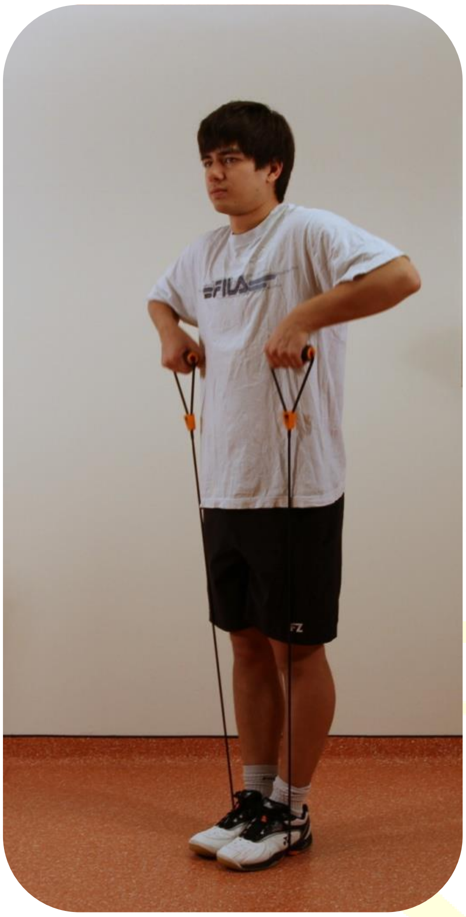

Procedure

- Pak de uiteinden van de band vast met beide handen. Je staat boven op het midden van de band. Je armen hangen recht naar beneden en je houd de band goed vast.
- Trek de uiteinden van de band omhoog door je ellebogen te buigen. Je ellebogen wijzen naar buiten.
- Ga weer terug naar de startpositie en herhaal de oefening.

Tips

- Houd je bovenlichaam recht en houd de band dicht bij je lichaam.
- Je ellebogen moeten in uiteindelijk hoger komen dan je schouders.

Elastic Band Lateral Raise

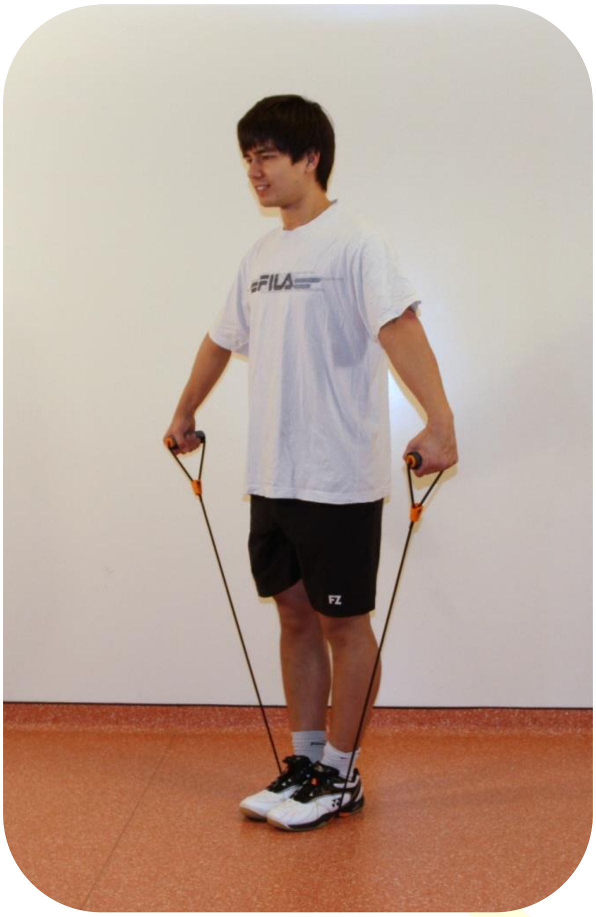

Procedure

- Pak de uiteinden van de band vast met beide handen. Je staat boven op het midden van de band. Je armen hangen recht naar beneden en je houd de band goed vast.
- Til je armen omhoog. Je armen blijven hierbij gestrekt.
- Ga weer terug naar de startpositie als je armen parallel zijn aan de vloer en herhaal de oefening.

Tips

- Houd je bovenlichaam recht door je buikspieren en rugspieren aan te spannen. .
- Je armen mogen niet hoger komen dan parallel aan de vloer.

Elastic Band Seated Row

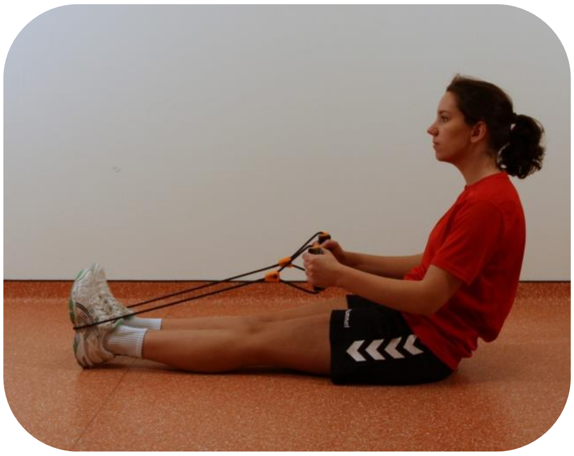

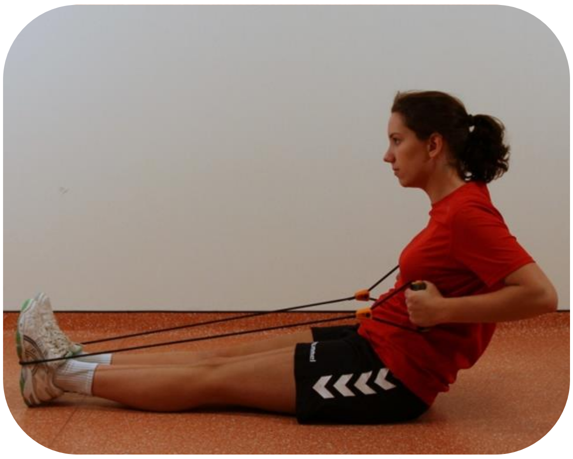

Procedure

- Je zit op de grond en pakt de uiteinden van de band vast met beide handen. De band gaat achter langs je voeten. Je armen zijn gestrekt. Houd je rug recht door je buikspieren en rugspieren aan te spannen.
- Trek de band naar je toe door je ellebogen te buigen en naar achteren te brengen.
- Ga weer terug naar de startpositie en herhaal de oefening.

Tips

- Let er op dat de band goed achter je voeten zit en niet los kan schieten. Je kunt je tenen een beetje naar voren laten wijzen om hier zeker van te zijn.
- Je kunt de band ook om een vast voorwerp draaien. Zo weet je zeker dat hij niet los kan schieten.

Elastic Band Biceps Curl

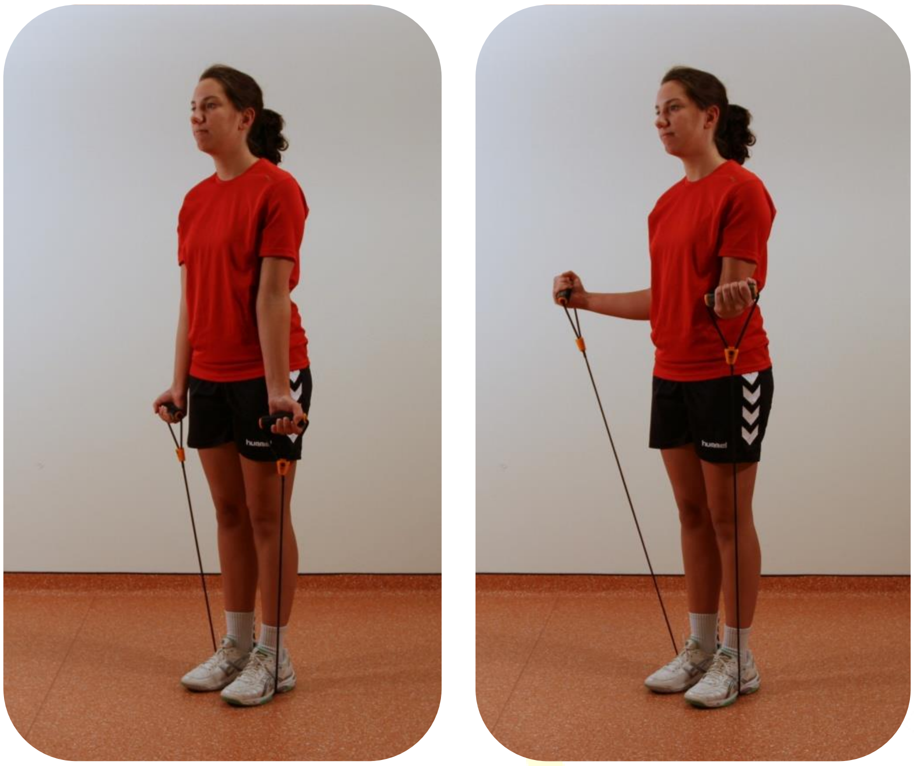

Procedure

- Pak de uiteinden van de band vast met beide handen. Je staat boven op het midden van de band. Je armen hangen recht naar beneden en je houd de band goed vast. De handpalmen wijzen naar voren.
- Trek de uiteinden van de band omhoog door je ellebogen te buigen. Je ellebogen wijzen naar de vloer.
- Ga weer terug naar de startpositie en herhaal de oefening.

Tips

- Houd je bovenlichaam recht en houd de band dicht bij je lichaam.
- Om nog rechter te staan kun je met je rug tegen de muur gaan staan.
- Stap niet van de band af als er spanning op staat.

Elastic Band Triceps Extension

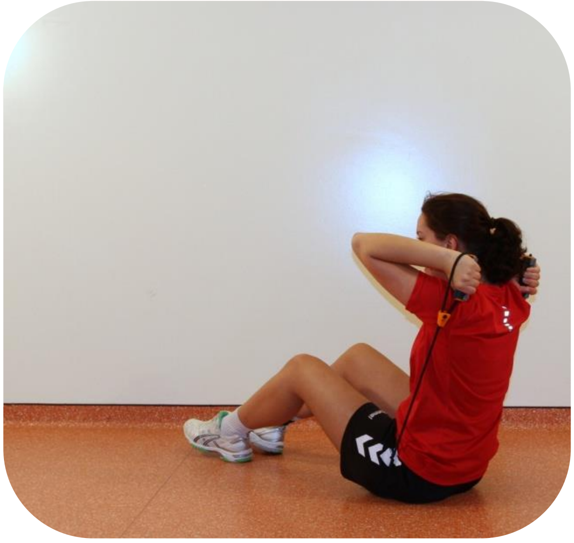

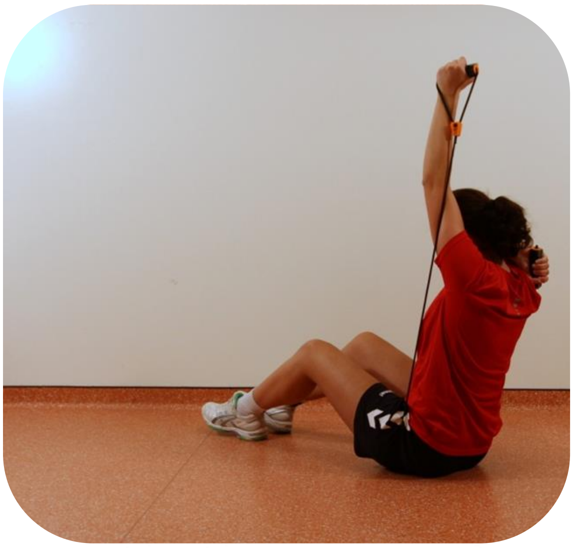

Procedure

- Pak de uiteinden van de band vast met beide handen. Je zit boven op het midden van de band. Je armen hangen achter je hoofd naar beneden en je houd de band goed vast.
- Trek de uiteinden van de band omhoog door je ellebogen te buigen. Doe dit een voor een. Je ellebogen wijzen naar boven.
- Ga weer terug naar de startpositie en herhaal de oefening.
- Wissel van arm.

Tips

- Houd je bovenlichaam recht en houd de band dicht bij je lichaam.
- Zorg dat de band niet los kan schieten.

Krachtoefeningen met apparaten

Leg Press

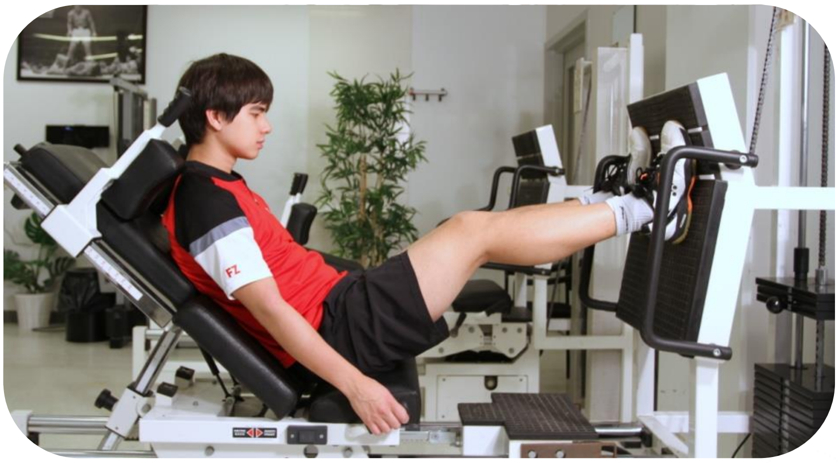

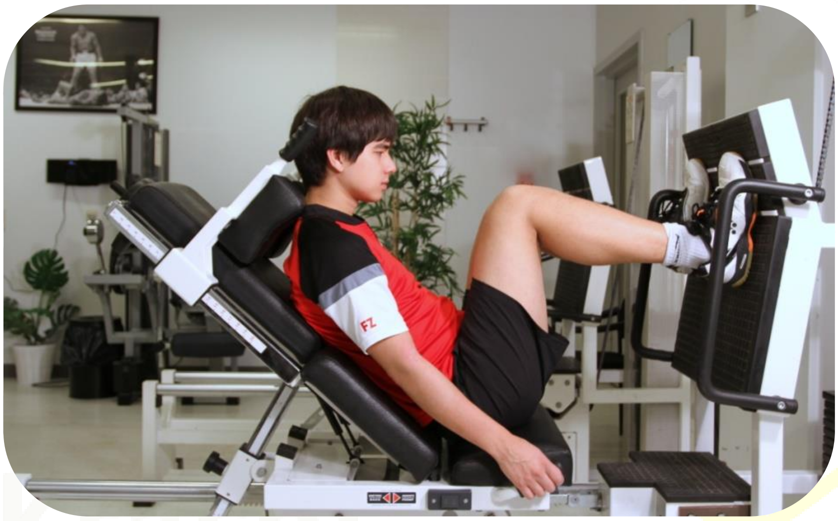

Procedure

- Stel de stoel zo in dat je met je benen in een hoek van 90 graden zit.
- Houd je rug stevig tegen de rugleuning. Zet beide voeten tegen het apparaat. Houd met je handen beide handvaten vast. Blijf met je billen goed op de stoel zitten.
- Duw met beide voeten totdat je benen bijna helemaal gestrekt zijn. Let er op dat je je knieën niet op slot zet.
- Buig langzaam je knieën en ga terug naar de start positie. Begin dan opnieuw met de beweging voordat het gewicht de andere gewichten raakt.

Tips

- Zet je knieën niet op slot als je je benen strekt (je knieën niet overstrekken).
- Houd je rug goed tegen de rugleuning.
- Houd je handen weg van bewegende delen.
- Voer de bewegingen rustig uit.

Leg Extension

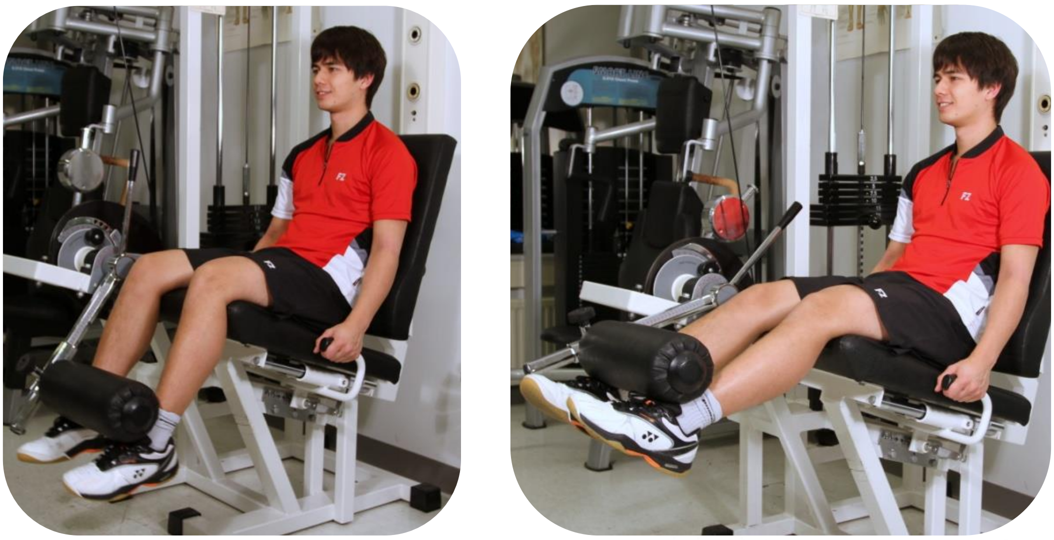

Procedure

- Ga op de stoel zitten en plaats je enkels achter de rol.
- Zit rechtop met je knieën gebogen en je rug stevig tegen de rugleuning. Houd de handvaten vast met beide handen.
- Til de rol op door je knieën te strekken.
- Ga terug naar de start positie door langzaam je knieën te buigen. Begin met de volgende beweging voordat het gewicht de andere gewichten raakt.

Tips

- Laat het gewicht niet te snel vallen bij het teruggaan naar de start positie.
- Het bovenlichaam hoeft niet te bewegen tijdens deze oefening.
- Zorg ervoor dat je je knieën niet overstrekt.

Chest Press

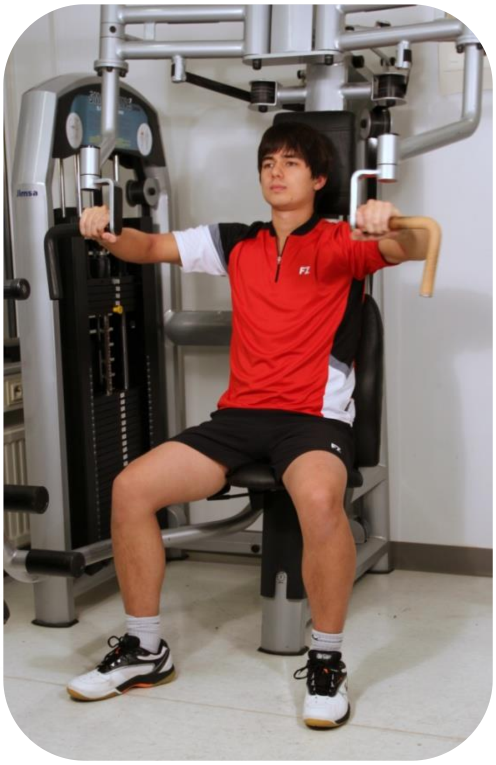

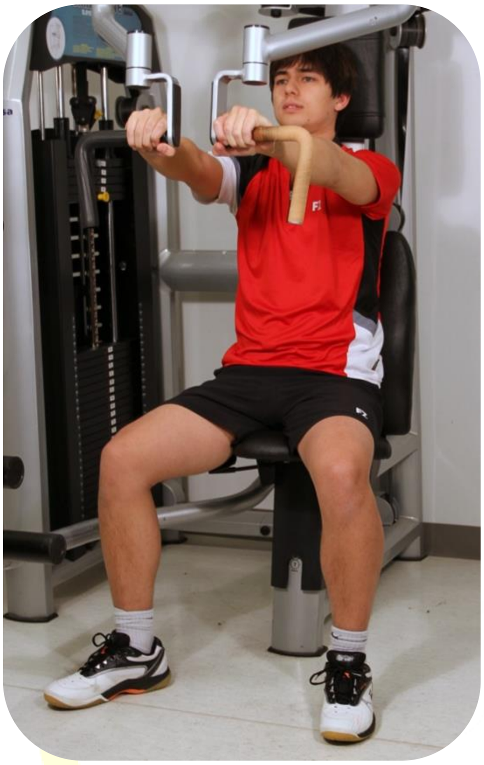

Procedure

- Zit op de machine met je rug en schouders tegen de rugleuning. Houd de handvaten vast met beide handen. De stoel moet zo staan dat je ellebogen op gelijke hoogte zitten met je schouders.
- Duw de handvaten naar voren door je armen te strekken.
- Ga langzaam terug naar de begin positie (ellebogen op één lijn met de schouders).

Tips

- Zet je ellebogen niet op slot als je je armen strekt (ellebogen niet overstrekken).
- Houd je rug recht en tegen de rugleuning tijdens de oefening.
- Laat je ellebogen bij het terugtrekken niet voorbij je schouders komen.

Seated Row

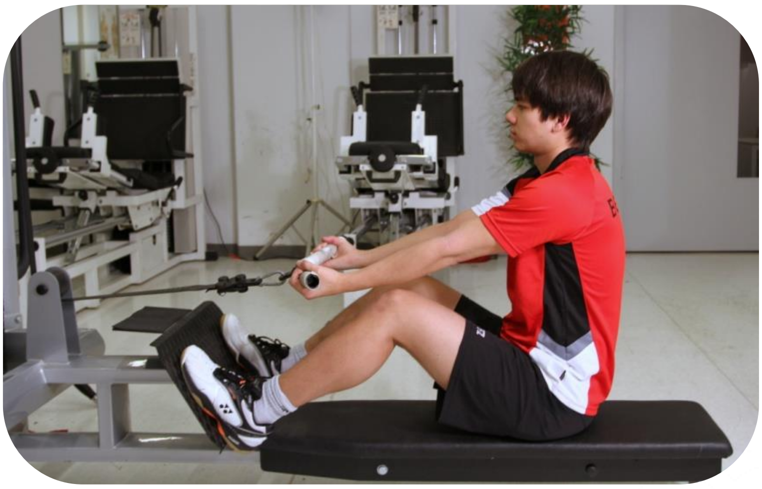

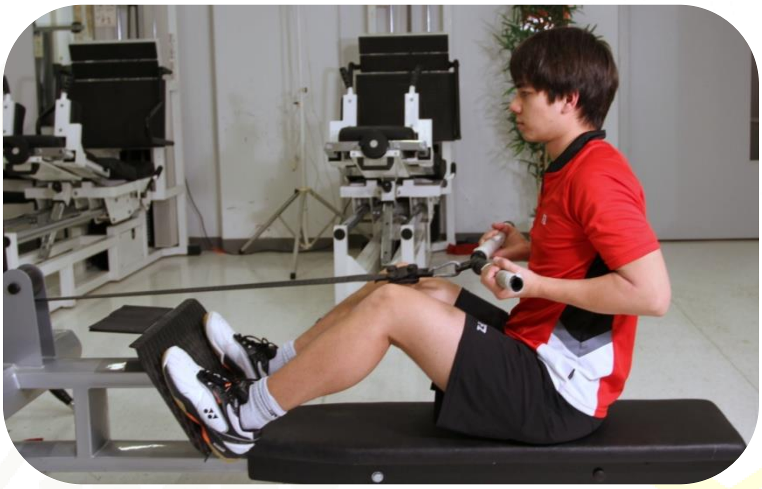

Procedure

- Zit op de machine met je voeten tegen de voetsteun. Houd de handvaten vast met beide handen.
- Trek de handvaten naar je toe tot je borst.
- Ga langzaam terug naar de begin positie en begin de volgende beweging wanneer het gewicht de anderen nog niet raakt.

Tips

- Hou je rug recht tijdens de oefening.

Front Pull-Down

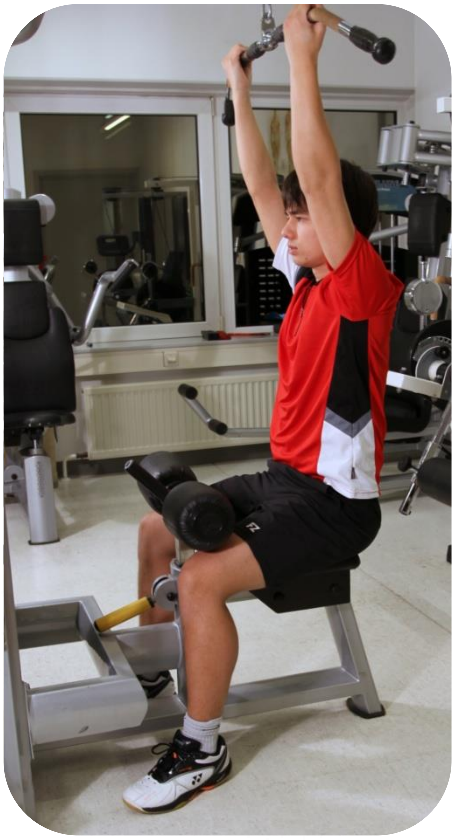

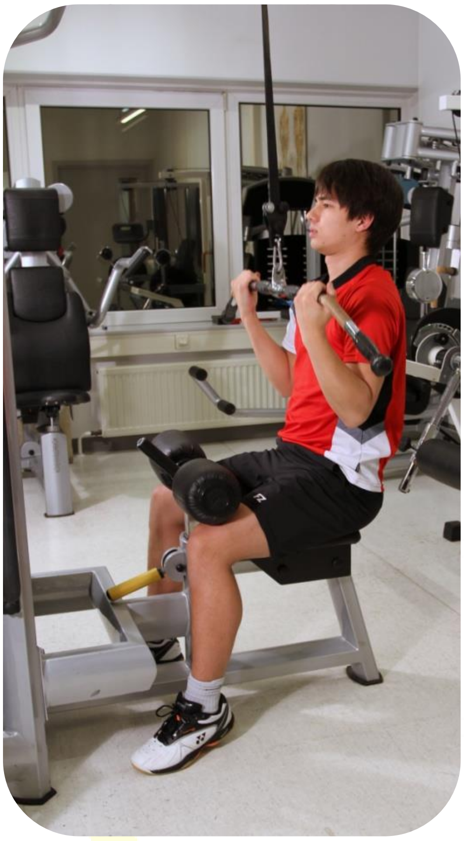

Procedure

- Pak de handvaten vast met beide handen. Zit op de stoel en plaats de knieën onder de rol zodat deze niet bewegen. Houd je bovenlichaam en armen recht.
- Trek langzaam de handvaten naar je toe. Laat hierna langzaam het gewicht terugzakken. Strek je armen weer helemaal.

Tips

- Draai niet met je bovenlichaam tijdens de oefening. Gebruik alleen je armen en rug om de oefening uit te voeren.
- Let op dat je gezicht uit de buurt blijft van de handvaten en andere bewegende onderdelen van het apparaat.
- Het handvat kan ook achter het hoofd naar beneden worden getrokken.

Overhead Press

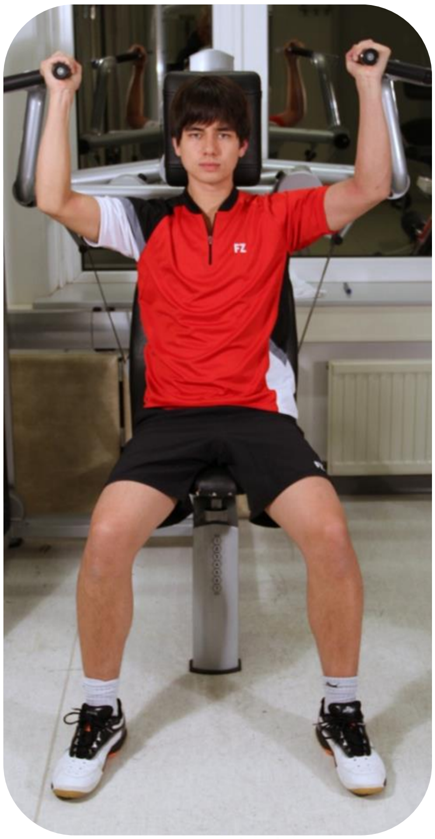
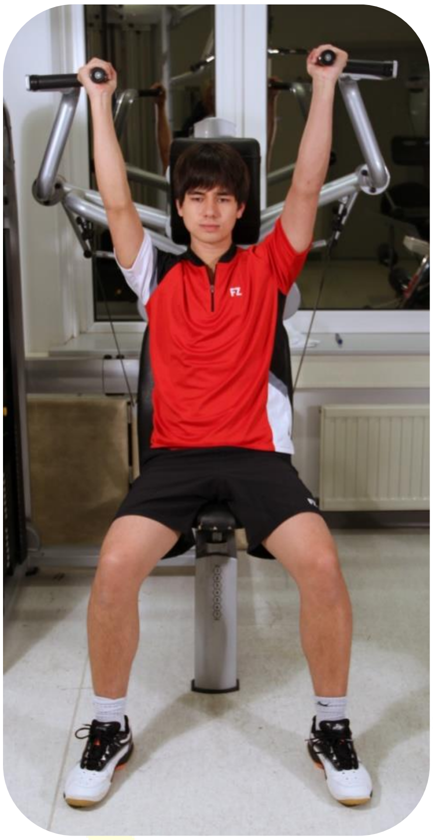

Procedure

- Zit met je rug tegen de rugleuning. Houd met beide handen de handvaten vast.
- Duw de handvaten naar boven totdat je armen bijna helemaal gestrekt zijn.
- Laat de handvaten langzaam zakken en begin de volgende beweging voordat het gewicht de andere gewichten raakt.

Tips

- Zet je ellebogen niet op slot als je ze strekt.
- Houd je rug stevig tegen de rugleuning.

Notities

Notities

Notities

Notities

de Kracht

van

Kracht

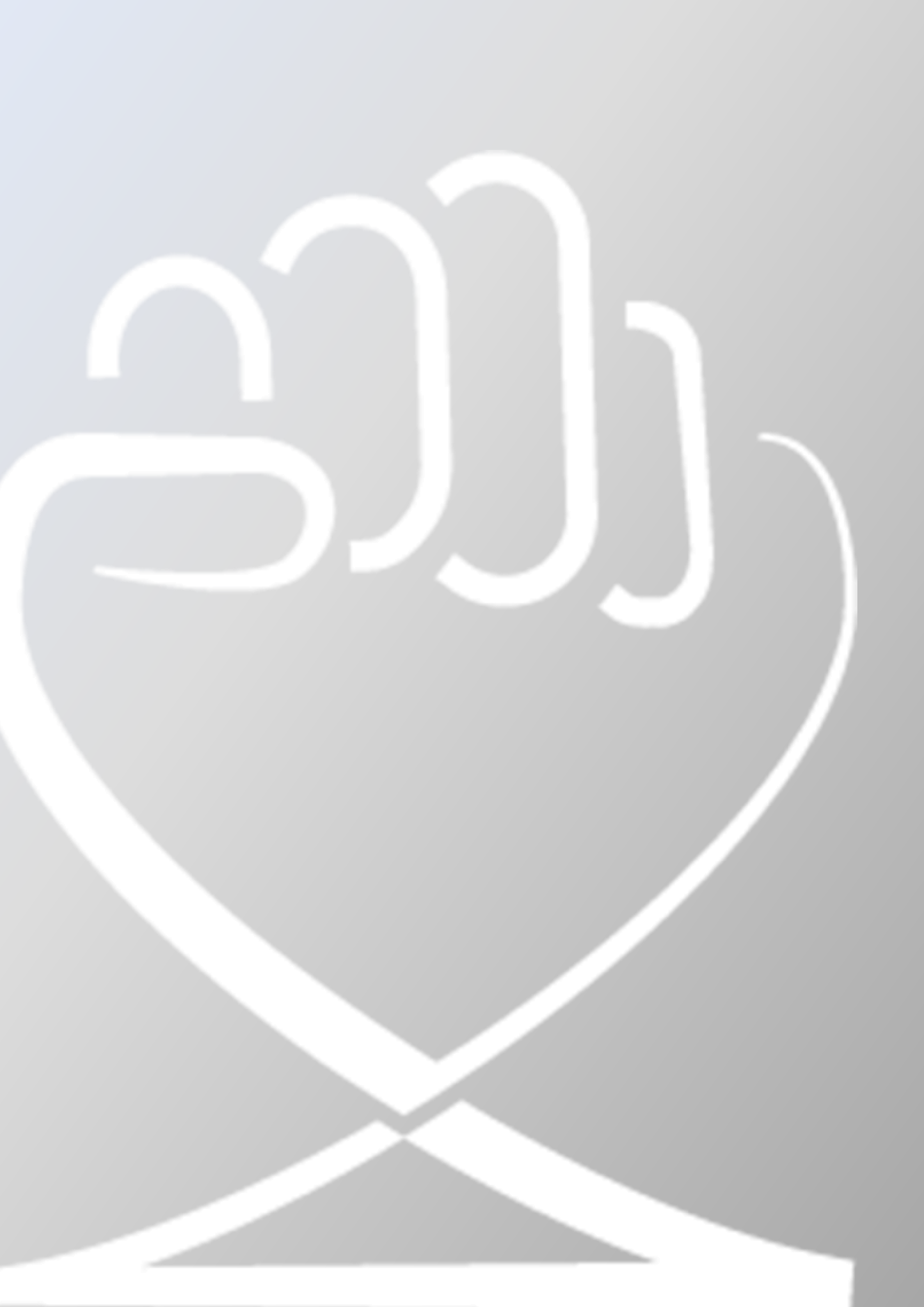

Supplement: Additional file 1 — Inspirational Materials for PE teacher. (PDF 4455 kb) [file 12889_2016_3150_MOESM1_ESM.pdf]
