# Supplementary material for: The Dutch ‘Focus on Strength’ intervention study protocol: programme design and production, implementation and evaluation plan
Source: BMC Public Health. 2016 Jun 10;16:496. doi: 10.1186/s12889-016-3150-6 (PMC4902907; doi:10.1186/s12889-016-3150-6)

Docentenhandleiding

DE KRACHT VAN KRACHT

Motivationele interventie

-Samen op zoek naar bewegen-

September 2015

Voorwoord

Vanuit de Universiteit Maastricht ontwikkelen we een programma dat zich richt op het promoten van fysieke activiteit van jongeren. Het is een programma voor middelbare scholen, met enerzijds in een deel van de lessen lichamelijke opvoeding aandacht voor krachtinspanningen, anderzijds in een deel van de mentorlessen aandacht voor de motivatie om te bewegen. Voor u ligt de docentenhandleiding voor de motivationele interventie: "DE KRACHT VAN KRACHT – Samen op zoek naar beweging."

In deze handleiding willen we samen met u – stap voor stap – niet alleen op zoek naar geschikte manieren om meer te bewegen voor de jongeren in uw klas, maar proberen we ook gezamenlijk mogelijke barrières op te lossen en gaan we op zoek naar de motivatie voor een actieve levensstijl.

Geert Rutten en Gill ten Hoor
Namens het Kracht van Kracht project-team

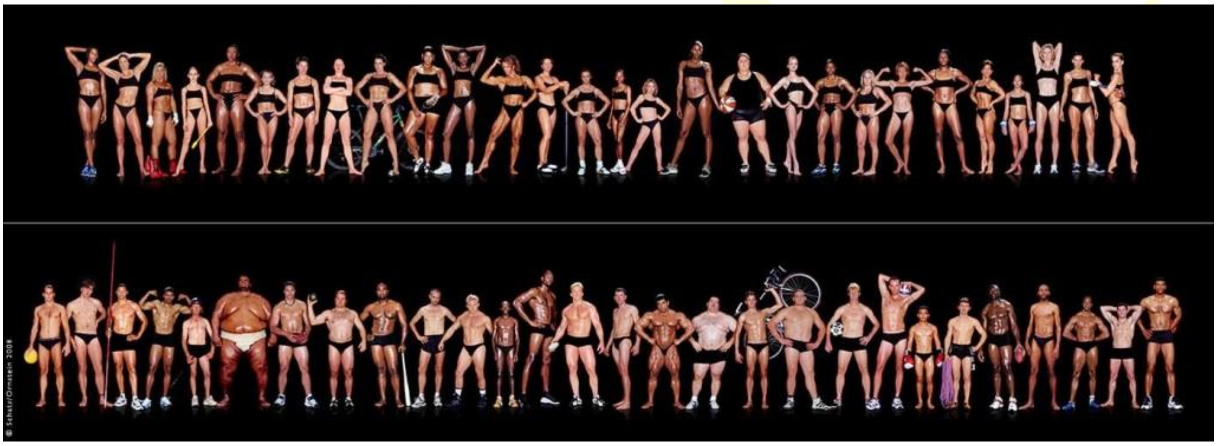

Achtergrond van het programma

Overgewicht is een groot en groeiend probleem. Hoewel het probleem eenvoudig op te lossen lijkt (minder eten en meer bewegen), is het in de praktijk niet zo simpel. In de eerste plaats moet iemand niet alleen gezonder willen eten en meer willen bewegen, maar moet men ook het gevoel hebben dat ze dat kunnen. Ook wanneer het 'willen en 'kunnen' geen probleem is, kan het zijn dat iemand gewoonweg niet de kennis heeft over wat nu precies goed is en wat niet.

Veel beweegprogramma's en activiteiten die overgewicht willen voorkomen richten zich vooral op conditie en niet op kracht. Jongeren met overgewicht hebben vaak een minder goede conditie dan jongeren zonder overgewicht, waardoor ze niet gemotiveerd zijn om aan die beweegprogramma's mee te doen. Wij willen juist op kracht gerichte beweegactiviteiten aanbieden tijdens de gymles waar jongeren met overgewicht goed in zijn, die ze leuk vinden, en die effectief zijn. In het onderzoek combineren we nieuwe inzichten uit de biologie en de psychologie tot één beweegprogramma dat bestaat uit zowel een kracht-gerelateerd als motivationeel deel. Voor jongeren met overgewicht heeft een op kracht gebaseerd programma zowel biologische als psychologische voordelen. Deze jongeren hebben meer lichaamsmassa en daarmee ook meer spiermassa. Hierdoor zijn ze beter in kracht- dan in conditie-gerelateerde inspanningen én sterker dan hun dunnere leeftijdsgenootjes. In ons programma zullen deze jongeren ervaren dat ze hier beter in zijn, waardoor ze het leuker vinden en daardoor meer gemotiveerd raken. Door krachttraining zal de verhouding tussen spier en vet verbeteren waardoor ook de stofwisseling verbetert en lange termijn gezondheidseffecten haalbaar zijn.

In dit programma zullen we echter niet de nadruk leggen op overgewicht.

Dit programma is voor alle jongeren. Bovenstaande gezondheidsvoordelen gelden ook voor jongeren zonder overgewicht!

De motivationele lessen

Gedurende één schooljaar zal er – naast de gymlessen waarin krachtinspanningen terugkomen - 1x per maand een motivationele groepsles plaatsvinden. Voor deze les wordt één volledig lesuur ingeruimd, waarin gezamenlijk gekeken zal worden naar onder anderen wat de jongeren nu doen aan beweging, wat ze graag willen en wat ze weten over de voor- en nadelen van bewegen. Voor het doormaken van het individuele motivationele proces is het van belang dat kinderen zich veilig voelen. Het kan bijvoorbeeld zo zijn dat kinderen zich in een les met de gehele klas terughoudend opstellen. Daarom krijgen de leerlingen naast de klassikale lessen in de eerste 5 maanden ook een online opdracht, waarin (mits de leerlingen niet worden afgeleid door klasgenoten) privacy wordt geboden. Deze online opdrachten zijn te vinden via de website www.krachtvankracht.nl.

Het lesprogramma

Les 1. Inventarisatie (klassikaal).

Doel: In dit lesuur zal worden geïnventariseerd hoe fysiek actief de klas is. Na deze les kan de online opdracht worden gemaakt (zie les 2). Deze online opdracht kan direct na de groepsles worden gemaakt, maar bijvoorbeeld ook twee weken later worden gepland (dit is per school en klas verschillend). De online les dient als voorbereiding op de volgende groepsles (les 3).

Introductie (klassikaal 10-15 minuten)

Introduceer de motivationele interventie met deze tekst als leidraad.

Dit is de eerste motivationele groepsles van het jaar. In deze lessen, die we 1 keer per maand hebben, gaan we het hebben over sporten en bewegen. Hierbij gaat het bijvoorbeeld over vragen zoals wat je eigenlijk leuk of niet leuk vindt aan sporten en bewegen, of het belangrijk is, wat je al doet en of je misschien liever iets anders of iets meer zou doen en wat dat dan zou zijn. Behalve de lessen met de klas krijgen jullie ook nog wat opdrachtjes, waarvoor je naar de website www.krachtvankracht.nl gaat, die bij dit programma hoort. Zo'n opdrachtje maak je steeds vóór de volgende groepsles. Je krijgt een eigen gebruikersnaam en wachtwoord voor de website. Op dat deel van de website waar jij je opdrachtjes maakt, kan niemand anders van je klas kijken, dat is en blijft van jou alleen.

Deel de klappers + persoonlijke naam/wachtwoord kaartjes uit die gemaakt zijn voor de leerlingen. Geef aan wanneer deze online opdrachten worden gemaakt (ofwel direct na de groepsles, ofwel na een week of 2; let op: ook de online opdrachten moeten op school worden gemaakt, maar dan achter de computer).

Mogelijk hebben de leerlingen deze kaartjes met hun unieke code al ontvangen. Geef aan dat de leerlingen de website, hun gebruikersnaam en wachtwoord in hun agenda, en in hun telefoon moeten opslaan.

Inventarisatie fysieke activiteit

1. De scholieren wordt gevraagd om (ieder voor zich) op te schrijven hoe vaak en hoeveel ze fysiek actief zijn. Dit doen ze op bijgevoegd formulier dat ze (in tweevoud) vinden in hun klapper (zie volgende pagina). Het is belangrijk dat er een onderscheid wordt gemaakt tussen bewegen in het dagelijkse leven (bijvoorbeeld: naar school fietsen, buiten spelen) en sporten (bijvoorbeeld: wekelijks in team- of clubverband sporten). **Zorg ervoor dat dit duidelijk is voordat de scholier dit invult. Geef ook aan dat ze op beide formulieren hetzelfde invullen, en dat ze één van deze formulieren dadelijk met hun unieke code erop (dus niet hun wachtwoord! Wanneer ze deze niet bij zich hebben kunnen ze hun naam**

invullen) inleveren bij de docent. Het tweede formulier kunnen ze toevoegen aan hun persoonlijke klapper.

2. Nadat de scholieren dit formulier hebben ingevuld, leveren ze dit in bij de docent. **Zorg ervoor dat de scholieren de totaalscores ook voor zichzelf ergens opschrijven. Het is niet de bedoeling de individuele scores klassikaal te bespreken.** De docent zal een lijst maken waarin alle scores zijn opgenomen door de scores in de bijgeleverde Excel -sheet in te voeren. Deze lijst zal worden gezien als 'basis fysieke activiteit'. Tijdens het invoeren van de cijfers (max 5 minuten) kan het volgende filmpje worden getoond.

Dance battle

https://www.youtube.com/watch?v=rnM8XsuKi0M (30 seconden tot 4 minuten)

3. Koppel **het gemiddeld aantal minuten** dat de klas sport en beweegt (zowel qua sporten als qua 'overige activiteiten') terug. Een deel van de leerlingen zal meer dan gemiddeld sporten en bewegen, een deel minder dan gemiddeld, en een aantal leerlingen zal precies gemiddeld sporten en bewegen. Laat dit gemiddelde aan de klas zien. Hierover kan een open discussie worden gevoerd uitgaande van de vraag 'Wat valt jullie hier aan op?' **Vraag niet aan de kinderen om klassikaal aan te geven of zij boven of onder dat gemiddelde zitten.** Als ze het uit zichzelf vertellen, moedig, in het kader van de *sociale vergelijking* waar we zorgvuldig mee om moeten gaan, anderen dan niet aan om dat ook te doen. Vraag vervolgens aan de leerlingen om in hun eigen klapper op te schrijven of zij meer, minder of evenveel als de hele klas lichamelijk actief zijn.

Let op: Bewaar de ingevulde formulieren en schrijf de gemiddelde scores ook op: deze hebben we in les 11 opnieuw nodig!

Persoonlijke code: ………………………….

HOEVEEL SPORT IK?
Schrijf hieronder op hoeveel je sport **buiten de gymlessen op school**. Dit kan bijvoorbeeld een sport zijn bij een in club (bijvoorbeeld voetbal, tennis, breakdance enz.) of op een sportschool.

	Welke sport	Hoe vaak per week?	Hoeveel minuten per keer?	Totaal aantal minuten per week
Sport 1				
Sport 2				
Sport 3				
Sport 4				
Sport 5				
BEREKEN JOUW TOTAAL AANTAL MINUTEN PER WEEK				

HOE ACTIEF BEN IK NAAST HET SPORTEN?
Denk hierbij aan beweegactiviteiten die je doet naast het sporten, zoals bijvoorbeeld lopen of fietsen naar school, potje voetbal, rolschaatsen, voor je plezier zwemmen en zo.

	Welke activiteit	Hoe vaak per week?	Hoeveel minuten per keer?	Totaal aantal minuten per week
Activiteit 1				
Activiteit 2				
Activiteit 3				
Activiteit 4				
Activiteit 5				
BEREKEN JOUW TOTAAL AANTAL MINUTEN PER WEEK				

Les 2 Bewustwording en ambivalentie (Online).

[ONLINE LESSEN DIE MOETEN VERSCHIJNEN OP PAGINA VAN LEERLING (WANNEER INGELOGD). Elke maand is maar 1 les toegankelijk. De maand erna zijn alle lessen ervoor in te zien, maar niet meer te bewerken. De informatie voor de online lessen is verwerkt in deze klapper, zodat jullie als docent op de hoogte zijn van wat de leerlingen online doen]

Doel: **Bewustwording en ambivalentie.**
Inschatting van het eigen niveau van lichamelijke activiteit en al nadenken over de voor en nadelen van wel of niet actief zijn/sporten.

1) Geef een punt voor hoe lichamelijk actief (sporten en bewegen) je bent. Een 0 betekent bijvoorbeeld dat je helemaal niet lichamelijk actief bent (dan zou je dus bijna de hele dag zitten of op de bank liggen), een 10 betekent dat je heel veel en vaak lichamelijk actief bent en een 5 bijvoorbeeld dat je best wel, maar ook niet heel erg veel lichamelijk actief bent. Kies (klik op) het cijfer dat het beste bij jou past.

Ik ben (bijna) niet actief — Ik ben heel erg veel actief

0 1 2 3 4 5 6 7 8 9 10

2) Kijk eens naar het cijfer dat je hebt gegeven. Bedenk waarom je niet 2 punten minder hebt gegeven en schrijf dat op. Als je meer dan één reden hebt schrijf die dan ook maar allemaal op. (wanneer de leerling een twee of lager heeft gegeven wordt gevraagd waarom dit cijfer is gegeven)

………

………

………

3) Bedenk 2 redenen (mag ook meer zijn) waarom je **niet** zou sporten en bewegen en schrijf ze op.

………

………

………

4) Bedenk 2 redenen (mag ook meer zijn) waarom je **wél** zou sporten en bewegen en schrijf ze op.

……

……

……

5) Stel je gaat meer bewegen dan je nu doet. Schrijf hieronder op wat je dan zou gaan doen (hoeft niet per sé op een sportclub te zijn). Het mag ook meer dan één ding zijn.

……

……

……

6) Je hebt gekozen voor [antwoorden op vraag 5]. Wat zou je daarmee willen bereiken? Als je meer dan 1 ding wil bereiken, schrijf die dan allemaal op.

……

……

……

Les 3. Voor en nadelen van bewegen (klassikaal).

Doel: **Ambivalentie fase**
Positiebepaling van de kinderen voor wat betreft hun ambivalentie t.a.v. sporten en bewegen. De kinderen laten nadenken over de voor- en nadelen van niet of wel sporten en bewegen. Dit is een eerste stap op weg naar verandering.

De kinderen hebben in de eerste online les opgeschreven wat ze zouden willen gaan doen als ze meer actief zouden worden. De daarop volgende vraag was wat ze ermee zouden willen bereiken (doel). Mogelijk is dat een lastige vraag voor de kinderen, dus even navragen en eventueel even op weg helpen met deze vraag. Probeer dit te doen met niet-suggestieve, open vragen (dus niet: “vind je dat omdat je er misschien fit van wordt?” maar “waarom vind je het belangrijk?”)

Voor en nadelen van lichamelijke activiteit

1) Op het bord worden 4 vlakken getekend. 1) voordelen van niet sporten/bewegen, 2) nadelen van niet sporten/bewegen zijn, 3) nadelen van wel sporten en bewegen, 4) voordelen van wel sporten en bewegen zijn.

2) Laat de leerlingen eerst even voor zichzelf argumenten bedenken voor de 4 vlakken (voor formulier zie pagina 6). Laat ze daarna in 2-tallen werken (10 minuten). Deel de klas daarbij in twee helften. De 2-tallen in de ene helft bedenken de *voordelen van niet sporten en bewegen* en de *voordelen van wel sporten en bewegen*, de andere helft bedenkt de *nadelen van niet sporten en bewegen* en de *nadelen van wel sporten en bewegen*. **Gebruik post-its** die ze vervolgens in ‘hun’ vlakken op het bord plakken. **Geef hierbij duidelijk aan dat er geen goede en foute voor en nadelen zijn, alles is goed!** Vervolgens wordt op de antwoorden per vlak gereflecteerd. Herkennen de kinderen wat er op de post-its staat. Geef andere kinderen daarbij de mogelijkheid om vakken aan te vullen. **Let hierbij op de neutraliteit**. Het is niet de bedoeling dat er nu voor en nadelen gebagatelliseerd worden, want dat kan sommige kinderen het gevoel geven dat hun mening/idee er dus niet toe doet. Stel eventueel nieuwe **open vragen** (bv Wat maakt dan dat jullie dit een nadeel vinden?). Bespreek de vlakken daarbij in de bovenstaande (zie 1.) volgorde (voordelen van sporten en bewegen als laatste).

3) Wanneer dit gesprek ten einde loopt worden klassikaal alle voors en tegens nog eens opgeschreven op het bord (zie AMBIVALENTIEMATRIX op de volgende pagina). De kinderen schrijven die op in hun klapper!

4) Eventueel kan hierna nog eens de vraag worden gesteld: Wat valt jullie nou op als je dit ziet? Probeer hierbij **verandertaal** uit te lokken. Erken de voordelen van niet sporten en bewegen en de nadelen van wel sporten en bewegen. Leg daar niet de nadruk op (bijvoorbeeld blijven doorvragen waarom iets een nadeel is lokt juist behoudtaal uit), geef aandacht aan de voordelen van lichamelijk actief zijn (bijv Wat maakt dan dat je dit een voordeel vindt? Wat levert het je dan op?).

AMBIVALENTIE MATRIX

Voordelen van<br>**niet** sporten en bewegen	Nadelen van<br>**niet** sporten en bewegen
Nadelen van<br>**wel** sporten en bewegen	Voordelen van<br>**wel** sporten en bewegen

Les 4. Belangrijkheid van bewegen (Online).

Doel: **Kennis vergaren over normen t.a.v. sporten en bewegen en zitten. Inventarisatie van het belang dat ze hechten aan voldoende sporten en bewegen en een bewustwording van de redenen waarom ze dat belang eraan hechten.**

1) Waarom denk je dat bewegen goed voor je is?

………

………

………

Gebruik de volgende websites om de antwoorden op de volgende 2 vragen op te zoeken:

http://www.30minutenbewegen.nl/home-ik-voer-campagne/over-de-campagne/de-beweegnorm.html

http://www.nisb.nl/weten/feiten_cijfers_trends-ontwikkelingen/lichamelijke-inactiviteit-en-sedentair-gedrag.html

http://www.nationaalkompas.nl/gezondheidsdeterminanten/leefstijl/lichamelijke-activiteit/normen-van-lichamelijke-in-activiteit/

2) Hoeveel tijd zou je per dag moeten bewegen: ....minuten

3) Hoe zit het dan met TV kijken/computeren (met een moeilijk woord ook wel ‘sedentair gedrag’ genoemd)? Hoeveel tijd mag je per dag TV kijken/computeren?

   ……………………minuten

Je geeft aan dat je per dag minstens [xx] minuten moet bewegen.

1) Denk je dan dat je dat zelf ook doet? Ja/nee

2) Stel je zou minstens 60 minuten per dag sporten en bewegen. Wat zou er voor nodig zijn dat je dat gaat doen. Kun je daar twee dingen (mag ook meer) voor noemen? Degenen die al meer dan 60 minuten per dag sporten en bewegen kunnen hier opschrijven hoe dat is gelukt, hoe ze dat hebben aangepakt en hoe ze dat volhouden.

………

………

………

Je geeft aan dat je per dag [xx] minuten TV mag kijken/mag computeren.

3) Hoeveel tijd per dag kijk jij, buiten schooltijd, TV en/of zit je achter de computer als je dat bij elkaar optelt?

……………………minuten

4) Stel je zit meer dan 2 uur per dag TV te kijken/aan de computer (tijd opgeteld). Wat zou er dan voor nodig zijn ervoor te zorgen dat je dat minder dan 2 uur doet? Schrijf dat hieronder op.

………

………

………

5) Geef een punt voor hoe belangrijk je het vindt om voldoende te sporten/bewegen. Een 0 betekent bijvoorbeeld dat je dat heel erg onbelangrijk vindt, een 10 betekent dat je het juist heel erg belangrijk vindt en een 5 bijvoorbeeld dat je best wel belangrijk vindt, maar ook weer niet zo heel erg belangrijk. Kies (klik op) dus het cijfer dat het beste bij jou past.

Heel erg onbelangrijk										Heel erg belangrijk
0	1	2	3	4	5	6	7	8	9	10

6) Als je naar je eigen cijfer kijkt, bedenk dan eens waarom je niet 2 punten minder hebt gegeven en schrijf dat op. Heb je meer dan 1 reden schrijf die dan allemaal op.

……

……

……

7) Wie vind jij een heel erg goede sporter?

Een goede sporter vind ik: ………………………………………………………

Schrijf hieronder op (mag meer dan één reden zijn) waarom je deze sporter zo goed vindt? Redenen:

……

……

……

Les 5. Belangrijkheid II (klassikaal).

Doel: Dat leerlingen zelf inzicht krijgen in een gezonde leefstijl. Daarnaast de bewustwording van het feit dat je niet perse een zeer atletisch uitziend lichaam hoeft te hebben om een goede sporter te kunnen zijn en dat ervoor alle (lichamelijke) eigenschappen sporten zijn waarin je veel kunt bereiken.

In deze les de huiswerkopdracht bespreken (mogelijk hebben niet alle kinderen die helemaal af gekregen):

- De antwoorden die ze hebben gevonden op de feitjeskennis vragen plenair bespreken. Alleen als kinderen vanuit zichzelf de informatie geven over hoe hun sport en beweeggedrag zich verhoudt tot de gevonden normen is dat prima, maar niemand is daartoe verplicht (**dus ook niet op aandringen**).
- **Als ze het uit zichzelf doen is het prima, maar kinderen hoeven hun eigen score voor belangrijkheid niet in de klas openbaar te maken!** Dat neemt niet weg dat gevraagd kan worden waarom ze niet 2 punten lager hebben gescoord – Een volgende belangrijke vraag is wat ze nodig zouden hebben om 2 punten hoger (van x naar y) te scoren. Gebruik hiervoor niet de vraag *'Waarom niet 2 punten hoger?'*, maar bij voorkeur een 'stel-vraag': *Stel, je zou het nóg belangrijker vinden dan je het nu al vindt (je zou bijvoorbeeld 2 punten hoger scoren) wat zou daar dan voor nodig zijn?* In tegenstelling tot de *Waarom-vraag* vermijd je hiermee dat de leerling het gevoel krijgt dat hij/zij te laag heeft gescoord, waardoor hij/zij 'in de verdediging' moet. Dat zou leiden tot behoudtaal, terwijl de stel-vraag eerder verandertaal uitlokt.

Plenair: *Jullie hebben al eens nagedacht over waar je zelf aan denkt bij sporten en bewegen. Stel nu dat je meer gaat bewegen, wat zou je dan willen doen en waarom zou je nou juist dát willen doen. Heb je er ook een bepaald doel mee, iets wat je met die sport zou willen bereiken? Degenen die al veel sporten zouden vooral hun doelen kunnen weergeven.*
Het is hier erg belangrijk dat juist ook kleine stapjes worden gewaardeerd. Als een kind dat niet sport zegt dat het 1x/week wil gaan wandelen o.i.d. moet dat positief ontvangen worden. Eventuele negatieve of wat minachtende reacties van andere kinderen zouden bespreekbaar gemaakt kunnen worden - iedereen heeft zijn eigen haalbare doelen!

Van het nu volgende deel is het doel dat kinderen zien dat verschillende sporten ook om verschillende lichamelijke kwaliteiten vragen. Voor sommige sporten is het makkelijker als je dun en licht bent, maar voor andere sporten is meer kracht en een zwaarder lichaam juist een voordeel

1) Vraag de studenten waar een goede sporter aan moet voldoen, welke eigenschappen moet die hebben (lichamelijk en evt ook geestelijk). Schrijf de antwoorden op het bord.
2) In de volgende filmpjes worden sporters die op het hoogste niveau hun sport bedrijven of hebben bedreven gepresenteerd. We hebben hier sporters geselecteerd met duidelijk andere fysieke eigenschappen, die ze in hun sport juist heel goed kunnen gebruiken.

Mannen: **Dennis vd Geest vs. Churandy Martina**

Dennis van der Geest

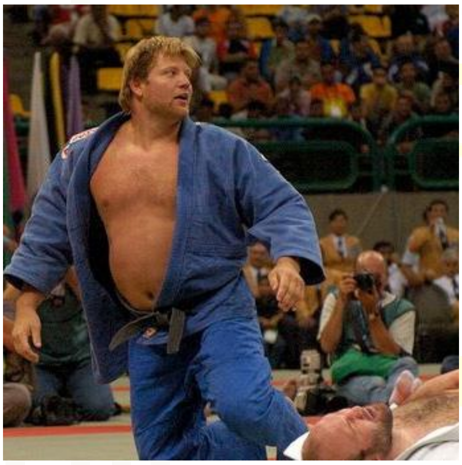

https://www.youtube.com/watch?v=GqqUaYh_LlI

Churandy Martina

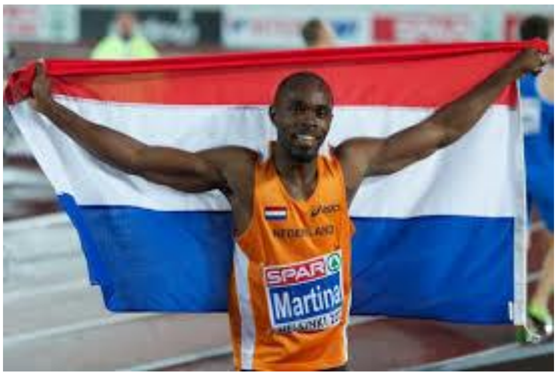

https://www.youtube.com/watyoutch?v=8dJPlRa9bOk   tot 50 seconden

Vrouwen: **Elisa Casanova vs Blanka Vlasic**

Elisa Casanova

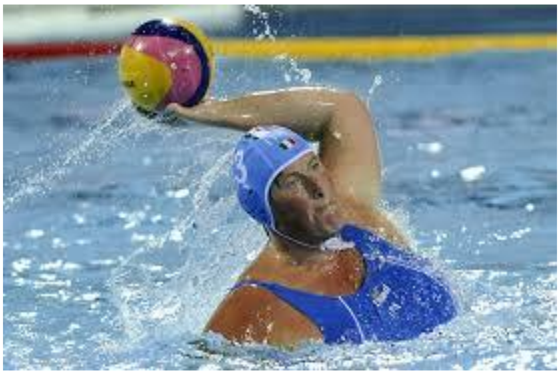

https://www.youtube.com/watch?v=w8G_gjQkaQ0

Blanka Vlasic

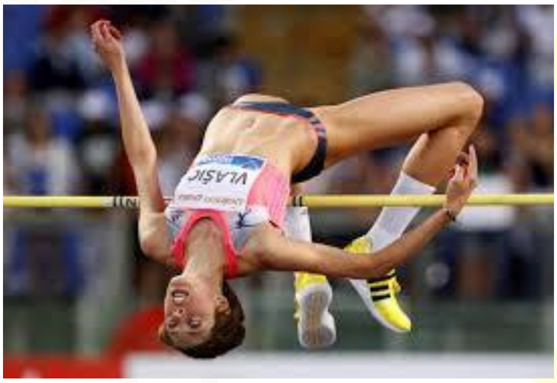

https://www.youtube.com/watch?v=0wlQN8uH8Bk (tot ongeveer 1.10)

Of eens iets heel anders, om te laten zien dat bewegen meer is dan alleen de sporten die we op tv zien.

Junior Company van het Nationaal Ballet
https://www.youtube.com/watch?v=dl1kk4Hg8B0

Dance battle
https://www.youtube.com/watch?v=rnM8XsuKi0M (30 seconden tot 4 minuten)

Maar dat kan dus ook als je wat zwaarder bent

https://www.youtube.com/watch?v=yoLmphvOkgc (van 2 – 4 minuten)

https://www.youtube.com/watch?v=dlihEYg34dg (15 sec tot 2.20 minuten)

https://www.youtube.com/watch?v=eqzUI5t9qJ4 (vanaf 45 sec tot ongeveer 2.03 min)

We zetten deze sport en beweegvormen eens tegenover elkaar. Zou Dennis (voormalig topjudoka en wereldkampioen) een goede hardloper zijn? En zou Churandy (Nederlands beste 100 en 200 meter loper met meerdere finale plaatsen of grote toernooien) een goede judoka zijn? En Elisa (een van 's werelds beste waterpolosters), zou die erg goed hoogspringen, of Blanka (wereld en Olympisch kampioene hoogspringen) lekker waterpoloën. Waarom dan wel/niet. Waarin ze vooral zien dat ieder zijn eigen talent kan hebben en dat je voordeel hebt van je lichaamsbouw in bepaalde sporten.

1) De leerlingen hebben in de huiswerkopdracht een sporter gekozen die ze heel goed vinden. Wat maakt nou dat ze die persoon zo goed vinden? Welke eigenschappen die hen aanspreken heeft die sporter dan?

In de hierop volgende online les zit een sportkeuzetabel (zie hieronder). Mogelijk is die voor de kinderen te ingewikkeld en is het beter die in de volgende klassikale les in te laten vullen. Daar is ruimte voor. De tabel zit ook in de map van de kinderen.

Opdracht: Welke sport past bij mij?

Gebruik de tabel op de volgende pagina (Lees eerst goed wat hieronder staat, zodat je weet hoe je de tabel moet gebruiken.)

1) Als je de vraag met JA kunt beantwoorden zet dan een 3 in het vakje. Bijvoorbeeld als een vriend of vriendin van jou atletiek doet zet je in dat vakje een 3
2) Als je een antwoord NIET WEET zet je een 1 in het vakje
3) Als je een vraag met NEE kunt beantwoorden laat je het vakje leeg
4) Bij nummer 22 en 23 kun je zelf nog een sport opschrijven die niet in de lijst staat. Vul ook daarvan dan de vakjes in. Het is **niet verplicht** om zelf een sport te bedenken. Vul 22 en/of 23 alleen in als je een sport mist waarvan je vindt dat die er wel bij moet staan.
5) Als je bij alle sport en beweegactiviteiten de vakjes hebt ingevuld kijk je onderaan in de tabel en zie je de opgetelde cijfers van elke kolom staan. In sommige kolommen zul je hoog scoren en in andere laag. Zo kun je zelf zien wat je leuk vindt aan een sport of beweegactiviteit.
6) Bedenk, als je de scores op de kolommen bekijkt welke sport(en) daar dan bij zou(den) passen en welke je dan leuk zou vinden om te doen. Schrijf die hieronder op.

Ook als je al sport is het leuk om deze tabel eens in te vullen.

Nr	Soort sport of beweegactiviteit	Vinden mijn ouders leuk	Doet mijn vriend of vriendin	Er zijn goede clubs in de buurt	Vind ik een leuke sport/ beweeg-activiteit	Wil je graag in de natuur sporten	Het is goed voor mijn conditie	Ik zou het vooral voor de wedstrijd doen	Het is een individu-ele sport (doe je alleen)	Het is een team-sport	**Totaal per sport**
1	atletiek										
2	badminton										
3	ballet/dansen										
4	(kick)boksen										
5	duursport (lopen of fietsen)										
6	fitness/aerobics										
7	bodybuilding										
8	golf										
9	hockey										
10	judo										
11	korfbal										
12	paardrijden										
13	roeien										
14	rugby										
15	skiën										
16	softbal/honkbal										
17	Vechtsport (bv karate, taekwondo)										
18	voetbal										
19	volleybal										
20	waterpolo										
21	zeilen										
22	wedstrijd-zwemmen										
23	eigen keuze …………………………										
24	eigen keuze …………………………										
	**Totalen**										

Les 6. Wat past bij mij? (Online).

Doel: Een stap verder in de motivatie. Opstap naar een sportkeuzen en, waarbij ook wordt gekeken naar de eigen (lichamelijke) eigenschappen.

Geef een punt voor hoe graag je een sport of beweegactiviteit zou doen (denk niet alleen aan sporten bij een club, maar bijvoorbeeld ook aan fietsen of dansen). Als je al sport geef dan een punt voor hoe graag je dat doet.

Nee, helemaal niet										Ja, heel erg graag
0	1	2	3	4	5	6	7	8	9	10

Kijk eens naar het punt dat je hebt gegeven en bedenk dan eens waarom je niet 2 punten lager hebt gegeven? Schrijf dat hieronder kort voor jezelf op. Heb je meer dan één reden schrijf die dan ook allemaal op.

....

....

....

Stel dat je het nóg fijner zou vinden om te sporten/bewegen, wat zou er dan voor nodig zijn om dit punt 1 of 2 punten hoger te krijgen? Schrijf dat hieronder op.

....

....

....

In de vorige les met de klas hebben we verschillende sporters gezien met verschillende eigenschappen. Met eigenschappen bedoelen we je *lichamelijke eigenschappen* (hoe snel je bent, hoe sterk je bent, of je een goed uithoudingsvermogen hebt en hoe gemakkelijk of moeilijk je bewegingen nauwkeurig en precies kunt uitvoeren), maar ook je *karakter eigenschappen* (doorzettingsvermogen, inzet, sport/beweeg je graag met anderen samen of liever alleen, wil je graag steeds beter worden in je sport, enzovoort).

Klik achter elke eigenschap het antwoord aan dat het beste bij jou past

	Niet	Best wel	Heel erg
Ik ben snel			
Ik ben sterk			
Ik heb een goed uithoudingsvermogen (kan sporten lang volhouden)			
Ik kan bewegingen gemakkelijk uitvoeren			
Ik leer graag nieuwe bewegingen			
Ik ben een doorzetter			
Ik doe er veel moeite voor			
Ik wil graag winnen			
Ik wil graag steeds beter worden			
Ik wil graag samen met anderen sporten			

Opdracht: Welke sport past bij mij?

Gebruik de tabel hieronder (Lees eerst goed wat hieronder staat, zodat je weet hoe je de tabel moet gebruiken.)

1) Als je de vraag met JA kunt beantwoorden zet dan een 3 in het vakje. Bijvoorbeeld als een vriend of vriendin van jou atletiek doet zet je in dat vakje een 3
2) Als je een antwoord NIET WEET zet je een 1 in het vakje
3) Als je een vraag met NEE kunt beantwoorden laat je het vakje leeg
4) Bij nummer 22 en 23 kun je zelf nog een sport opschrijven die niet in de lijst staat. Vul ook daarvan dan de vakjes in. Het is **niet verplicht** om zelf een sport te bedenken. Vul 22 en/of 23 alleen in als je een sport mist waarvan je vindt dat die er wel bij moet staan.
5) Als je bij alle sport en beweegactiviteiten de vakjes hebt ingevuld kijk je onderaan in de tabel en zie je de opgetelde cijfers van elke kolom staan. In sommige kolommen zul je hoog scoren en in andere laag. Zo kun je zelf zien wat je leuk vindt aan een sport of beweegactiviteit.
6) Bedenk, als je de scores op de kolommen bekijkt welke sport(en) daar dan bij zou(den) passen en welke je dan leuk zou vinden om te doen. Schrijf die hieronder op.

Ook als je al sport is het leuk om deze tabel eens in te vullen.

Nr	Soort sport of beweegactiviteit	Vinden mijn ouders leuk	Doet mijn vriend of vriendin	Er zijn goede clubs in de buurt	Vind ik een leuke sport/ beweeg-activiteit	Wil je graag in de natuur sporten	Het is goed voor mijn conditie	Ik zou het vooral voor de wedstrijd doen	Het is een individu-ele sport (doe je alleen)	Het is een team-sport	**Totaal per sport**
1	atletiek										
2	badminton										
3	ballet/dansen										
4	(kick)boksen										
5	duursport (lopen of fietsen)										
6	fitness/aerobics										
7	bodybuilding										
8	golf										
9	hockey										
10	judo										
11	korfbal										
12	paardrijden										
13	roeien										
14	rugby										
15	skiën										
16	softbal/honkbal										
17	Vechtsport (bv karate, taekwondo)										
18	voetbal										
19	volleybal										
20	waterpolo										
21	zeilen										
22	wedstrijd-zwemmen										
23	eigen keuze ……………………….										
24	eigen keuze ……………………….										
	**Totalen**										

Als je nu naar de tabel kijkt, bij welke sport zouden jou eigenschappen, die je eerder hebt aangegeven, dan het beste passen? Het mogen er ook meer dan één zijn. Schrijf die hieronder op (niet meer dan 4). *(eigenschappen keuze hier zichtbaar maken)*

1.
2.
3.
4.

En heb je bij die sport ook veel punten gescoord?

Kracht

Kracht

Les 7. Actieplanning (klassikaal, maar veel werken in eigen schrift).

Doel: De leerlingen gaan beginnen met het maken van een actieplan om dan ook daadwerkelijk een sport/beweegactiviteit op te starten. De vragen die hierbij moeten worden beantwoord zijn:

-Wat ga je concreet doen
-Waar ga je dat doen
-Hoe ga je dat aanpakken
-Waar heb je hulp bij nodig en van wie kun je die krijgen

(inventariseer in hoeverre het invullen van de tabel online is gelukt)

Eerst een vraag over het vertrouwen dat het zal lukken

Degenen die al een sport doen kunnen opschrijven of het ze veel moeite heeft gekost om daarmee te beginnen of juist niet en hoe dat dan kwam. Ze kunnen hetzelfde doen voor het vertrouwen dat ze het ook lang gaan volhouden.

Voor de kinderen die nog niet of weinig sporten en/of die wel een andere sport zouden willen doen of meer zouden willen sporten het volgende:

Jullie hebben een sport gekozen die je leuk vindt en misschien wel zou willen gaan doen. Schrijf in je schrift een punt tussen 0 en 10 voor het vertrouwen dat je erin hebt dat je het je lukt om met die sport te beginnen.

Geef nu een punt aan het vertrouwen dat je erin hebt dat je het je lukt om met die sport te beginnen/volhouden (voor meetinstrument zie pagina 17).

Helemaal geen Vertrouwen										Heel veel vertrouwen
0	1	2	3	4	5	6	7	8	9	10

Schrijf vervolgens eens kort op waarom je niet 2 punten lager hebt gegeven?

………

………

………

Stel je gaat meer bewegen/sporten, wat is er dan voor nodig om er nog meer vertrouwen in te krijgen (bijvoorbeeld 2 punten meer) dat je je doel bereikt?

………

………

………

Het actieplan

Om te beginnen pakken de leerlingen nog eens de voordelen van het bewegen, die ze eerder hebben bedacht, erbij. Laat ze eerst nog eens nadenken over wat ze eigenlijk wilden bereiken met sporten (bv 'Hoe vaak wil je dat doen?', 'Willen ze het gewoon voor hun lol doen, of willen ze bij de beteren/besten gaan horen?' **Geef daarbij aan dat het belangrijk is dat het ook een haalbaar doel moet zijn, dus geen té grote stap willen maken**.

Er zitten kinderen die al lang sporten in de groep en die dus geen actieplan nodig hebben. <u>Zij kunnen, aan de hand van dezelfde vragen, wel opschrijven hoe dat bij hun is gegaan</u>. In elke vraag is daarom met bold lettertype aangegeven welke vraag hen wordt voorgelegd. De antwoorden die zij geven zouden de kinderen die nu moeten starten kunnen helpen.

1) Laat elk in zijn eigen schrift kind opschrijven wat het ze ervoor nodig heeft **(gehad)** om het sportdoel te bereiken? Wat kun(**/kon)** je zelf doen en waar heb je hulp bij nodig **(gehad)**? Als je hulp nodig hebt **(gehad)**, van wie zou(**heb)** je die dan kunnen krijgen en waar kun je die **(/heb je die kunnen)** vinden?
   Een andere optie is om aparte groepjes te maken van kinderen die gaan beginnen met een sport en kinderen die al sporten en ze dan hetzelfde in die groepjes te laten doen

Vervolgens gaan we dit plenair bespreken, waarbij er ook weer geen sprake kan zijn van goede en fouten antwoorden. Elk kind moet zich erkend voelen. Het moet het karakter van een brainstorm hebben waarin alleen per vraag verschillende antwoorden worden geïnventariseerd. Probeer hierbij kinderen die al sporten te stimuleren aan te geven hoe dat bij hun is gegaan of hoe zij dat hebben aangepakt. In de volgende les wordt hierop terug gekomen

Vervolgens starten we met de actieplanning (voor formulier zie volgende pagina). De leerlingen gaan de antwoorden op de 'wat, waar, hoe en wie vragen' voor zichzelf opschrijven in hun klapper. Het uitgangspunt is een Stel-vraag: Stel, je gaat…(eigen voorkeursactiviteit invullen)..**voor kinderen die al sporten kan hier hun gestelde doel als uitgangspunt woorden genomen (het plan is er dus op gericht op hoe ze dat denken te gaan bereiken)**

2) Wat ga je doen om daarmee te kunnen beginnen? (moet je het bijvoorbeeld met iemand bespreken, moet er iemand worden gebeld om een afspraak te maken, ga je een keertje kijken of misschien heb je bepaalde sportkleding nodig… enzovoort)
3) Waar ga je dat dan doen?
4) Wat kun je zelf doen en waar heb je hulp bij nodig? Als je hulp nodig hebt, van wie zou je die dan kunnen krijgen en waar kun je die vinden?

[Ga verder op de volgende pagina]

Jouw ACTIEPLAN

Stel ik ga …………………………………………………………… (vul hier de sport of beweegactiviteit uit die je zou willen doen **of het doel dat je wil bereiken**)

Schrijf hier op wat er eerst moet worden geregeld voordat je kunt beginnen en wanneer je dat gaat doen

Wat ga ik doen.	Wanneer doe ik dat

Schrijf hier op waar je dat dan gaat doen

Schrijf hier op wat je zelf kunt doen

Schrijf hier op of je hulp nodig hebt en van wie dan

Schrijf hier op waar je die hulp kunt vinden

Les 8. Opstel (Online).

Doel: **Soort synthese van voorgaande lessen. Bovendien kunnen we hieruit opmaken hoe leerlingen er nu voor staan voor wat betreft hun motivatie voor sporten en bewegen. Dit geeft dan voeding aan het vervolg van de lessen.**

Schrijf een kort opstel (maximaal ongeveer 20 regels) wat je het allerliefst aan sport en bewegen zou doen en wat je daar het allerliefst mee zou bereiken. Geef ook aan waarom dat zo is. Zou je dat dan kunnen bereiken en als dat niet kan, wat zou je dan wél willen bereiken? Hoe belangrijk vind je het om dat doel te bereiken en wat maakt dat je het zo belangrijk vindt?

Les 9. Commitment (klassikaal).

De uitkomsten van de voorafgaande klassikale les over het actieplan en de online opdracht zijn het uitgangspunt voor deze sessie.

1) Heeft iedereen een doel waarvan hij denkt dat dat haalbaar is? **(De leerlingen mogen hun doel benoemen, maar dat is niet verplicht)**

2) Als we kijken naar eenieders aanpak om zijn doel te bereiken, kunnen we dan iets van elkaar leren? Hierbij dienen het actieplan en de plenaire bespreking van les 7 als leidraad.

3) Wat kunnen we van elkaar leren over:

   a) De stappen die we nemen om ons doel te bereiken
   b) Waar we onze activiteit kunnen doen
   c) Wat we zelf kunnen doen en bij wie we hulp kunnen vragen
   d) Kunnen we elkaar eventueel ook helpen

Les 10 (Online).

Je hebt in de vorige les met de klas een zogenaamd **ACTIEPLAN** gemaakt. Jullie hebben dat met elkaar besproken. Misschien heeft dat je nieuwe ideeën gegeven. Pas je actieplan dan aan.

Probeer in de komende weken maar eens te beginnen om de eerste stappen van je actieplan uit te voeren. Waarschijnlijk liggen veel sporten stil in deze tijd, maar je kunt bijvoorbeeld al eens kijken bij welke club je zou willen gaan kijken en of je de hulp kunt regelen die je nodig hebt.

Schrijf hier op wat er eerst moet worden geregeld voordat je kunt beginnen en wanneer je dat gaat doen

Wat ga ik doen.	Wanneer doe ik dat

Schrijf hier op waar je dat dan gaat doen

Schrijf hier op wat je zelf kunt doen

Schrijf hier op of je hulp nodig hebt en van wie dan

Schrijf hier op waar je die hulp kunt vinden

Les 11. (Inhaalles!)

Doel: Inhaalles.

Gebruik deze les:

1) om een oude les in te halen.

2) om een maand ‘rust’ in te bouwen.

3) voor andere activiteiten!

Les 12. Actief?(Klassikaal - ongeveer 1 a 2 maanden na les 10).

Doel: Vaststelling of leerlingen al activiteiten hebben ondernomen op basis van hun actieplan en 'waar de leerlingen nu staan' m.b.t. hun sport en beweegactiviteiten. Vaststellen van redenen van wel of niet slagen in het opstarten van hu activiteit.

Zorg dat je als docent de gemiddelde activiteitenscore van de allereerste les bij de hand hebt.

De leerlingen vullen het activiteitenschema weer in.

1. De scholieren wordt gevraagd om (ieder voor zich) op te schrijven hoe vaak en hoeveel ze fysiek actief zijn. Dit doen ze op bijgevoegd formulier (zie volgende pagina). De leerlingen vinden hiervan twee stuks in hun leerlingenwerkboek (bij les 11). Belangrijk is het dat er een onderscheid wordt gemaakt tussen bewegen in het dagelijkse leven (bijvoorbeeld: naar school fietsen, buiten spelen) en sporten (bijvoorbeeld: wekelijks in team- of clubverband sporten). **Zorg ervoor dat dit duidelijk is voordat de scholier dit invult. Geef ook aan dat ze op beide formulieren hetzelfde invullen, en dat ze één van deze formulieren dadelijk inleveren bij de docent (let op: leerlingen moeten hun gebruikersnaam of naam op het formulier schrijven!)**

2. Nadat de scholieren dit formulier 2x hebben ingevuld, leveren ze één exemplaar in bij de docent (de andere voegen ze toe aan hun werkboek). De docent zal een lijst maken waarin alle scores zijn opgenomen door de scores in de bijgeleverde Excel - sheet in te voeren. **Zorg ervoor dat de scholieren de totaalscores ook voor zichzelf ergens opschrijven. Het is niet de bedoeling de individuele scores klassikaal te bespreken.**

3. In de tussentijd krijgen de leerlingen de opdracht een lijstje te maken met redenen waarom ze al dan niet zijn begonnen me de uitvoering van hun actieplan uit les 10).

   (mogelijk tot invullen in het werkboek).

   1. Ben je al begonnen met het uitvoeren van je actieplan? Als dat nog helemaal niet is gelukt, schrijf dan eens alle redenen op die er bij jou voor hebben gezorgd dat dat wel of niet is gelukt. Dat kunnen redenen zijn van jou zelf, maar het kan ook komen door dingen die buiten jou zelf liggen.

   2. Als je naar je lijstje kijkt zijn er dan redenen die je noemt waar je zelf iets aan zou kunnen doen? Zet daar dan achter **'kan ik zelf iets aan doen'.** En zijn er redenen waarvan je denkt dat je daar juist helemaal niks aan kunt veranderen? Zet daar dan achter **'kan ik helemaal niks aan doen'.**

4. Koppel **het gemiddeld aantal uren** dat de klas sport en beweegt (zowel qua sporten als qua 'overige activiteiten') terug. Vergelijk dit gemiddelde met de score uit de eerste les. Is dit meer, minder of hetzelfde. Laat dit aan de klas zien. Hierover kan een open discussie worden gevoerd uitgaande van de vraag 'Wat valt jullie hier aan op?' Probeer hierbij een gesprek te voeren over de redenen waarom de score nu is zoals die is in vergelijking met het begin. Wat hadden de kinderen verwacht m.b.t. de score t.o.v. het begin. Wat maakt dat het meer, minder of hetzelfde is? Het moet dus eigenlijk een klassikaal gesprek worden over wat kinderen de afgelopen weken hebben gedaan en dan ook ingaan op wat ze met hun actieplan hebben gedaan – eerste gesprek over mogelijke barrières die ze tegen zijn gekomen. Waarom is het sommigen wel gelukt en anderen niet? **Hiervoor kun je het lijstje met redenen dat ze zojuist hebben gemaakt gebruiken.**

5. **Geef leerlingen de opdracht mee om in de komende twee maanden eens een sport te gaan proberen waarvan zij zelf denken dat die bij hen past!**

6. **Laat iedereen alvast opschrijven wat hij/zij wil gaan proberen, of laat leerlingen in tweetallen afspraken maken zodat het concreter wordt.**

HOEVEEL SPORT IK?

Schrijf hieronder op hoeveel je sport **buiten de gymlessen op school**. Dit kan bijvoorbeeld een sport zijn bij een in club (bijvoorbeeld voetbal, tennis, breakdance enz.) of op een sportschool.

	Welke sport	Hoe vaak per week?	Hoeveel minuten per keer?	Totaal aantal minuten per week
Sport 1				
Sport 2				
Sport 3				
Sport 4				
Sport 5				
BEREKEN JOUW TOTAAL AANTAL MINUTEN PER WEEK				

HOE ACTIEF BEN IK NAAST HET SPORTEN?

Denk hierbij aan beweegactiviteiten die je doet naast het sporten, zoals bijvoorbeeld lopen of fietsen naar school, potje voetbal, rolschaatsen, voor je plezier zwemmen en zo.

	Welke activiteit	Hoe vaak per week?	Hoeveel minuten per keer?	Totaal aantal minuten per week
Activiteit 1				
Activiteit 2				
Activiteit 3				
Activiteit 4				
Activiteit 5				
BEREKEN JOUW TOTAAL AANTAL MINUTEN PER WEEK				

Les 13. (Inhaalles!)

Doel: Inhaalles.

<u>VRAAG/HERINNER LEERLINGEN AAN DE OPDRACHT 5 EN 6 VAN LES 12.</u>

Gebruik deze les daarnaast:

1) om een oude les in te halen.

2) om een maand 'rust' in te bouwen.

3) voor andere activiteiten!

Les 14. Ervaring en Actie. (Klassikaal - ongeveer 2 mnd na les 11).

Doel: Ervaringen van de leerlingen gebruiken om hen te helpen bij het actie ondernemen om een sport te gaan doen.

In les 12 is de opdracht meegegeven om een sport te gaan proberen die bij de leerling past. Er is een grote kans dat leerlingen geen extra actie ondernomen hebben.

1. We gaan een debat voeren over de stelling: **"Iedereen moet sporten".** De klas wordt in twee groepen verdeeld (Groep A is absoluut vóór deze stelling; Groep B is absoluut tégen deze stelling).

Beide groepen krijgen 10 minuten de tijd om argumenten te bedenken voor hun eigen stelling. Moedig leerlingen van groep B aan om vooral na te denken over **barrières** (tegenargument) en leerlingen van groep A om ook na te denken over **oplossingen** voor mogelijke barrières (argument voor) te bedenken.

Daarna gaat het debat beginnen. Het debat duurt maximaal 10 minuten, tenzij het erg goed loopt, dan mag het langer duren. Als gespreksleider houd je het goede verloop van het gesprek in de gaten: Geen aanvallen op de persoon, argumenten moeten aangrijpen op de inhoud. Breng eventueel wat nieuwe voeding voor het debat in als het vast dreigt te lopen. Vat regelmatig samen en verduidelijk standpunten als dat nodig is. Nodig ook wat stillere kinderen uit om iets te zeggen, maar dwing ze niet. Bewaak vooral het proces.

Vat aan het einde het debat kort samen en wat er uit is gekomen. Er is geen goede of foute uitkomst. Je mag de kinderen zeker ook complimenteren als ze goed hebben gedebatteerd.

2. Vervolgens doen we het nog een keer, maar nu moeten de groepen de volgende stellingen verdedigen:

Groep 1 verdedigt de stelling: "**Lid worden van een sportclub is nergens goed voor**"

Groep 2 verdedigt de stelling: "**Lid worden van een sportclub is het beste wat je kunt doen**"

De groep die in ronde 1 voorstanders van sporten waren, zijn nu tegenstanders en andersom.

[Ga door op de volgende pagina]

Beide groepen krijgen weer 10 minuten de tijd om argumenten te bedenken. In dit tweede debat hopen we vooral dat mogelijke barrières om bij een sportclub te gaan boven tafel komen. Ook focussen we direct op de oplossingen. Mochten er aan het einde van het debat barrières blijven bestaan, probeer dan samen met de leerlingen oplossingen te bedenken voor deze barrière.

3. Vraag aan de kinderen of ze hier nog iets aan hebben voor de uitvoering van hun eigen plan. Wat zouden ze verder nog nodig hebben voor de stappen die ze nog moeten maken. Waar zouden ze dat dan vandaan kunnen halen of wie zou ze daarbij kunnen helpen?

LES 15. Implementatie Intenties (Klassikaal).

Doel: **Bestendiging, voorkomen van terugval**

De focus van deze les ligt op de kinderen die zijn begonnen met sporten. Hoe gaan zij ervoor zorgen dat ze dat ook blijven doen. Daar kunnen *implementatie intenties* bij helpen. Een *implementatie intentie* is een eenvoudige strategie die helpt bij het omgaan met moeilijke situaties, omdat je voor die situatie een oplossing bedenkt en formuleert.

De kinderen gaan dus eerst bedenken in welke situaties ze het moeilijk vinden om te gaan sporten. Dat kan bijvoorbeeld zijn omdat het slecht weer is, of omdat een vriendje heeft gevraagd om te gamen op die tijd.

Laat ze in hun werkboek de onderstaande vragen beantwoorden. Bespreek dit vervolgens plenair.

Lukt het je om steeds te gaan sporten (bv iedere keer naar de training gaan)?

☐ Ja

☐ Nee

Wat maakt dat het soms niet lukt of dat je het soms erg moeilijk vindt om te gaan sporten (noem 3 redenen)

……

……

……

Bedenk nu een oplossing of enkele oplossingen voor jezelf, waardoor je toch gaat sporten als je dat lastig vindt

……

……

……

Maak nu een zin of enkele zinnen waarin je de moeilijke situatie en de oplossing aan elkaar knoopt. Hieronder staat een voorbeeld:

Je blijft bijvoorbeeld liever thuis als het regent terwijl je moet gaan sporten, omdat het anders door en door nat wordt. De zin zou dan kunnen zijn:

ALS het regent terwijl ik moet gaan sporten,

DAN zorg ik ervoor dat ik droge kleren mee neem naar de training.

Maak nu zelf eens voor al jou moeilijke situaties zo'n zin:

ALS………

……

DAN……

……

ALS………

……

DAN……

……

ALS………

……

DAN……

……

ALS………

……

DAN……

……

Extra Notitie blaadje

Notities

Kracht
Kracht

Extra Notitie blaadje

Notities

Extra Notitie blaadje

Notities

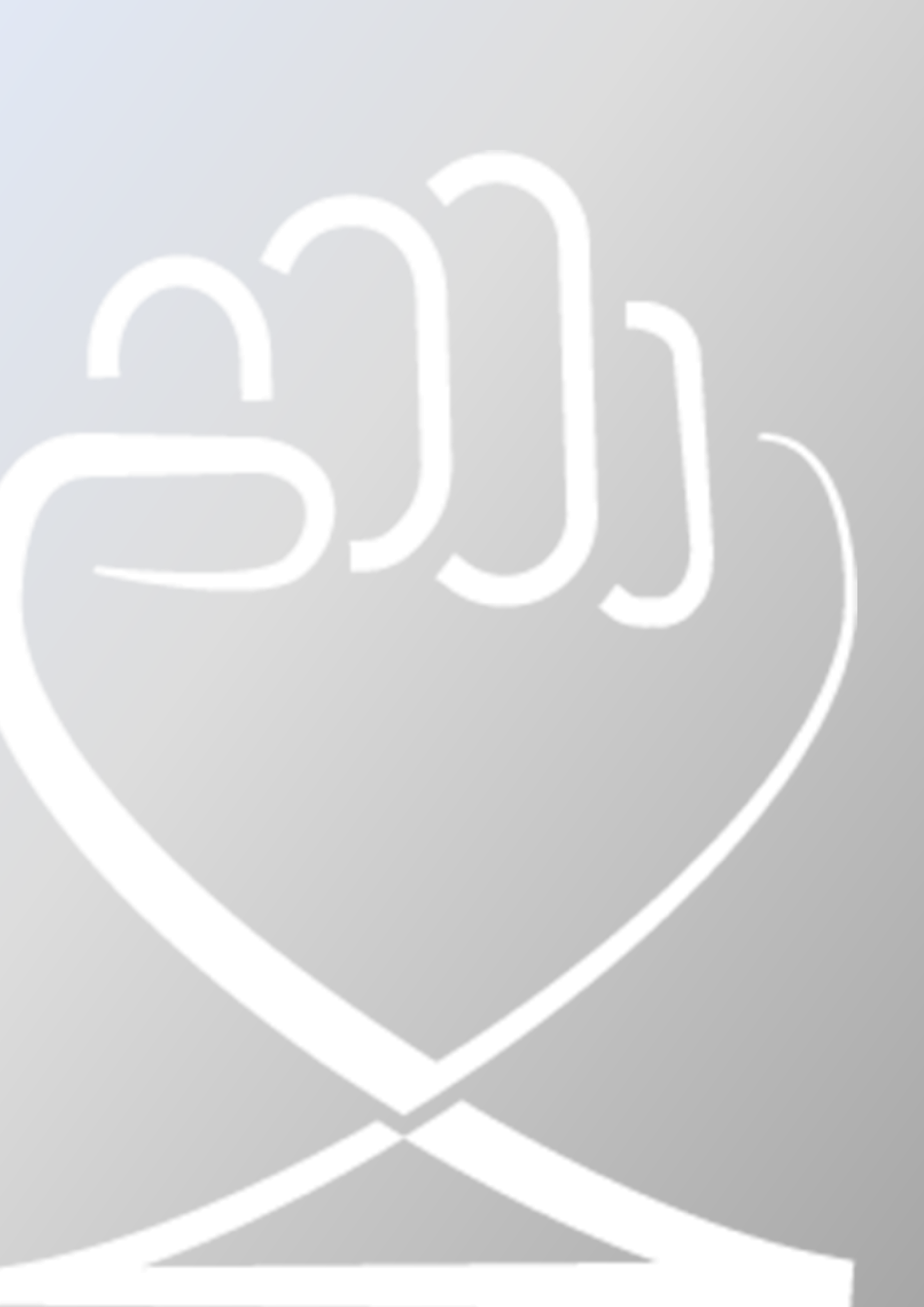

Supplement: Additional file 2 — Content of the motivational lessons (Dutch). (PDF 1002 kb) [file 12889_2016_3150_MOESM2_ESM.pdf]
